# Collaborative Care for Opioid Use Disorder and Mental Illness: The CLARO Randomized Clinical Trial

**DOI:** 10.1001/jamainternmed.2025.7036

**Published:** 2025-12-29

**Authors:** Katherine E. Watkins, Karen Chan Osilla, Colleen M. McCullough, Beth Ann Griffin, Alex R. Dopp, Kirsten Becker, Lisa S. Meredith, Valerie Carrejo, Grace M. Hindmarch, Sapna Mendon-Plasek, Jasen Christensen, Rebecca Weir, Lauren Kelly, Lia Pak, Cristina Murray-Krezan, Lina Tarhuni, Christina Crowley, Alexandra Bilder, Mariah M. Kalmin, Michael Schoenbaum, Miriam Komaromy

**Affiliations:** 1 RAND, Santa Monica, California; 2 Stanford University School of Medicine, Palo Alto, California; 3 RAND, Arlington, Virginia; 4 The University of New Mexico Health Sciences Center, Department of Family & Community Medicine, Albuquerque; 5 RAND, Boston, Massachusetts; 6 The University of New Mexico School of Medicine, Albuquerque; 7 National Institute of Mental Health, Bethesda, Maryland; 8 Grayken Center for Addiction, Boston Medical Center, Boston University, Boston, Massachusetts; 9 University of Pittsburgh School of Medicine, Pittsburgh, Pennsylvania; 10 The University of New Mexico Health Sciences Center, Albuquerque; 11 Department of Health Policy and Management, Fielding School of Public Health, University of California Los Angeles

## Abstract

**Importance:**

Adults with opioid use disorder (OUD), co-occurring with depression and/or posttraumatic stress disorder (PTSD), may benefit from collaborative care. Although collaborative care is an evidence-based model to treat behavioral health conditions in primary care, it has not been widely tested for OUD with co-occurring mental illness.

**Objective:**

To determine whether collaborative care, tailored for low-resource settings, improves outcomes for patients with OUD and co-occurring depression and/or PTSD more so than enhanced usual care (EUC).

**Design, Setting, and Participants:**

This 2-group single-masked pragmatic randomized clinical trial was conducted in 18 primary care clinics in California and New Mexico from January 8, 2021, to December 5, 2023, and included adult participants with probable OUD as well as major depression and/or PTSD. Data analysis was performed August 2024 to May 2025.

**Interventions:**

Six months of a care manager and addiction psychiatrist working with a primary care practitioner to deliver evidence-based treatments for OUD, major depression, and PTSD; care managers were community health workers who monitored and addressed biopsychosocial symptoms and referred patients for psychotherapy.

**Main Outcomes and Measures:**

Primary outcomes at 6 months were time to first filled buprenorphine prescription for participants entering the study not taking medication for OUD; cumulative days of prescribed buprenorphine for participants entering the study while not taking methadone, obtained from the state Prescription Drug Program; and Patient Health Questionnaire-9 and PTSD Checklist for *DSM-5* scores from participant surveys. Secondary outcomes included days of opioid and other drug use, the Patient-Reported Outcomes Measurement Information System’s substance use short form, and the Veterans RAND 12-item health survey.

**Results:**

Of 2396 participants assessed for eligibility, 797 were randomized (397 to EUC; 400 to intervention); 433 (54.3%) were female and 364 (45.7%) male, with a mean (SD) age of 40.2 (11.9) years. Six-month survey retention was 68% for EUC and 64% for collaborative care. Adjusted mean (SD) differences on primary outcomes for collaborative care vs EUC were: 7.0 (95% CI, −3.4 to 17.4; *P* = .19) for days until first buprenorphine prescription; 4.3 (95% CI, −7.4 to 16.0; *P* = .47) for cumulative days of prescribed buprenorphine; −1.0 (95% CI, −2.3 to 0.3;* P* = .13) for Patient Health Questionnaire-9 score; and −0.9 (95% CI, −4.6 to 2.8; *P* = .63) for PTSD Checklist for the *DSM-5* score; none were statistically significant. There were also no observed differences between groups for any secondary or exploratory outcomes; both groups improved over baseline. As-treated exploratory analyses showed evidence for improvements in OUD outcomes in the as-treated population.

**Conclusions and Relevance:**

This randomized clinical trial found that among participants with OUD co-occurring with depression and/or PTSD, collaborative care did not demonstrate evidence of greater improvements in outcomes compared with EUC. Explanations include a possible spillover effect, spontaneous improvement, or the fit of collaborative care for clinically complex patients and settings with few behavioral health practitioners.

**Trial Registration:**

ClinicalTrials.gov Identifier: NCT04559893 and NCT04634279

## Key Points

**Question:**

Does a 6-month collaborative care intervention, tailored for low-resource settings, improve clinical outcomes for adults with opioid use disorder and co-occurring depression and/or posttraumatic stress disorder more so than enhanced usual care?

**Findings:**

In this randomized clinical trial of 797 participants, collaborative care showed no evidence of improved outcomes compared to enhanced usual care. Both groups improved over the 6-month follow-up.

**Meaning:**

The findings suggest that for clinically complex patients, collaborative care may not be more effective than enhanced usual care; explanations include the potential for spillover, spontaneous improvement, and the fit of collaborative care for this population and setting.

## Teaser

This randomized clinical trial investigates whether collaborative care, tailored for patients with opioid use disorder and co-occurring depression and/or posttraumatic stress disorder, improves outcomes more so than enhanced usual care.

## Introduction

Opioid use disorder (OUD) is a public health crisis affecting from 6 to 9 million adults in the US and is associated with high rates of morbidity, mortality, and health care costs.^1,2^ OUD frequently co-occurs with mental disorders; patients with both types of disorders are less likely to be retained in treatment and have higher rates of mortality than those with a single disorder.^3,4,5,6^ Co-occurring mental illnesses contribute to increased risk of suicide and overdose compared to OUD alone, with depression and posttraumatic stress disorder (PTSD) being specific risk factors.^7,8,9,10,11^ Depression and/or PTSD affect from 18% to 52% of adults with OUD.^4^

Effective treatments exist, yet fewer than 20% of adults with co-occurring substance use and mental illness report receiving treatment for both conditions.^12^ Among individuals with OUD, only half report receiving either OUD or mental health treatment,^12^ and only 25% report receiving medications for OUD.^13^ This is important because untreated OUD, depression, and PTSD are each associated with morbidity and mortality, and individuals with co-occurring disorders are more likely to experience adverse social conditions than those with 1 disorder alone.^14,15^

Primary care may be an important setting in which to provide treatment for all 3 disorders, especially in areas with a shortage of behavioral health practitioners.^16,17^ Patients also may experience less stigma accessing primary care.^18^ Collaborative care is an evidence-based model to treat behavioral health conditions in primary care; however, it has not been widely tested for OUD with co-occurring mental illness. The objective of this study was to determine whether collaborative care is more effective than enhanced usual care (EUC) for primary care patients with OUD and co-occurring depression and/or PTSD.

## Methods

This study was approved by the RAND Institutional Review Board prior to the start of recruitment. All participants provided oral or written informed consent, and trial progress was regularly reviewed by an independent data and safety monitoring board. We report this study in accordance with the Consolidated Standards of Reporting Trials (CONSORT) reporting guideline. The trial protocol (Supplement 1) and statistical analysis plan (Supplement 2) were completed prior to study completion.^19^ Some planned secondary analyses were not included because of poor data quality.

### Study Design

The Collaboration Leading to Addiction Treatment and Recovery From Other Stresses (CLARO) study was a pragmatic randomized clinical trial of collaborative care in 18 primary care clinics in New Mexico and California. We randomized at the individual level based on a systematic review finding significant effects with individual randomization in 69 of 90 included studies (79%), along with positive effects in our previous study, which also used individual randomization.^20,21^ After August 29, 2022, 34 participants were randomized to receive an enhanced version of the intervention that included overdose and suicide prevention counseling; they were analyzed as part of the main trial.

### Study Population and Setting

The study was conducted within 4 health systems, including 14 primary care clinics in New Mexico and 4 clinics in California all serving low-income populations; 16 clinics were in locations identified as mental health professional shortage areas (MHPSAs).^22^ Clinics were not using collaborative care prior to study participation. Participants were screened and enrolled from January 8, 2021, to December 5, 2023. The final study visit was completed on June 12, 2024.

Participants were identified through universal prescreening in clinic waiting rooms, clinician referrals, and outreach based on diagnoses in clinic medical records. Potential participants were screened by study interviewers for eligibility and offered enrollment.

Participants were eligible for inclusion if they (1) were 18 years or older; (2) had probable OUD (defined by using medication for OUD [MOUD] for problems with opioids in the previous 90 days or scores of 1 or greater on the National Institute on Drug Abuse Tobacco, Alcohol, Prescription Medication and other Substance Use [myTAPS] screening tool, restricted to questions about opioid use)^23,24^; (3) had probable PTSD, defined as having a score of 3 or greater on the Primary Care PTSD Screen for *DSM-5*,^25^ and/or probable major depression, defined as a score of 10 greater on the Patient Health Questionnaire-8^26^; (4) considered the clinic to be their usual source of care; (5) and spoke English or Spanish.

### Study Randomization and Masking

At enrollment, study interviewers conducted a detailed baseline assessment and assigned participants to receive either collaborative care or EUC based on a 1:1 randomization protocol. A stratified randomization design was used, with the strata determined by site and an indicator for lifetime exposure to buprenorphine, methadone, or naltrexone, and included randomly permuted block sizes of 2 and 4. Research staff, except for interviewers at the point of randomization, were masked until database lock. Clinic staff were masked to patients receiving EUC.

### EUC

EUC group participants received usual primary care, which could include access to a nonstudy trained community health worker. We trained all interested behavioral health practitioners in problem-solving therapy (PST)^27,28^ for depression and written exposure therapy (WET) for PTSD,^29,30^ and offered training in the management of co-occurring disorders to primary care practitioners. Practitioners could apply training information to participants in both treatment groups.

### Intervention (Collaborative Care)

**Figure 1.**
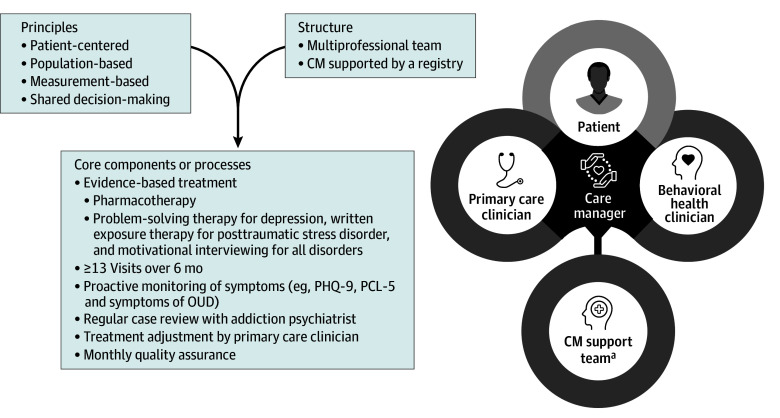
The CLARO Collaborative Care Model CM indicates care manager; PCL-5, Post-Traumatic Stress Disorder Checklist for *DSM-5*; PHQ-9, Patient Health Questionnaire-9. ^a^Support team includes an addiction psychiatrist, a supervising psychologist, and a supervising community health worker.

Figure 1 shows the CLARO collaborative care intervention, described in detail elsewhere.^31^ We implemented CLARO using a community health worker as the care manager because of the shortage of medical professionals.^32^ Care managers received 40 hours of training and met with participants for 13 visits over 6 months; activities included patient education, symptom monitoring, outreach and engagement, addressing the social determinants of health, active referrals for psychotherapy, and motivational interviewing. Patients were offered psychotherapy through referral to either a co-located or offsite study-trained behavioral health practitioner. Care managers entered encounters, contact attempts, regular assessments of symptoms and adverse effects (ie, measurement-based care),^33^ and information about social determinants of health into a caseload tracking tool.^34^ This tool was used to prioritize patients for discussion in a weekly caseload review with the addiction psychiatrist, and monthly case conferences. The psychiatrist made pharmacotherapy and psychosocial treatment recommendations to the primary care practitioner and care manager and provided guidance to the care manager on engagement approaches and motivational interviewing techniques.

Prior to enrollment, implementation facilitators (A.D. and S.M.P.) from the research team engaged with clinic leadership to form implementation teams and engage in readiness planning activities.^35^ Once enrollment began, facilitators held monthly meetings with the implementation teams to monitor intervention delivery by sharing aggregate patient data on treatment utilization, to engage in quality improvement activities, and to address sustainment planning. We have reported on fidelity elsewhere.^36^

### Data Collection and Follow-Up

Participants received a baseline assessment prior to randomization, and follow-up assessments at 3 and 6 months. Most assessments were conducted by telephone and used a web-based data capture system. All assessments included the Pain, Enjoyment and General Activity (PEG) scale to measure pain ^37^; the Addiction Severity Index legal questions^38^; and other assessments used to derive study outcomes. The baseline assessment also included participant self-reported demographic characteristics, housing status, the Alcohol Use Disorders Identification Test (AUDIT),^39^ receipt of MOUD, and mental health treatment utilization in the 30 days before enrollment. We derived a variable to describe type of trauma (none, trauma without interpersonal violence, trauma with interpersonal violence) by coding the worst traumatic event anchoring the PTSD Checklist for the *DSM-5* (PCL-5).^40^ To comply with requirements of the US National Institutes of Health Helping to End Addiction Long-term Initiative program, we collected participant self-reported race and ethnicity. Participants were invited to select all applicable identities from the following: American Indian or Alaska Native; Asian; Black or African American; Hispanic, Latino, or of Spanish origin; Native Hawaiian or Pacific Islander; White; or other (participants were instructed to self-identify as other race if the provided categories did not match their identity; those endorsing more than 1 race category were assigned a value of more than 1 race). We obtained data on buprenorphine prescriptions from the New Mexico and the California Prescription Drug Monitoring Programs.^41,42^

Data on care manager encounters came from the caseload tracking tool. Any collaborative care was defined as having an intake encounter. Receiving the key elements was defined as having at least 2 care manager encounters, 2 assessments of OUD and mental health symptom severity, and a case review with the psychiatric consultant.^43^

### Primary, Secondary, and Exploratory Outcomes

The primary outcomes at 6 months were: (1) buprenorphine access, defined as the time to first buprenorphine prescription for patients with no MOUD in the 30 days before baseline; (2) buprenorphine continuity, defined as the cumulative days of prescribed buprenorphine after enrollment for participants not entering the study while taking methadone; (3) depression symptom severity for participants with probable major depression at baseline, measured using the PHQ-9^26^; and (4) PTSD symptom severity for participants with probable PTSD at baseline using the PCL-5.^44^ We originally proposed to analyze MOUD access and continuity rates; however, because of missing methadone data, this was changed and approved prior to trial completion.

The secondary outcomes included: binary measures of depression remission and response, defined by a score of less than 5 on the PHQ-9 and a score less than 50% of the baseline score, respectively; binary measures of PTSD remission and response, defined by a PCL-5 score less than 33 and a score less than 50% of the baseline score, respectively; a binary measure of suicidal ideation in the past 30 days using the Columbia-Suicide Severity Rating Scale^45^; days with any opioid use in the past 30 days using an item from the US National Survey on Drug Use and Health^46^; number of opioid overdose events in the past 90 days using the Overdose Baseline Questionnaire^47^; and mental and physical health functioning, measured using the component scores from the 12-item Veterans RAND Health Survey.^48^

Exploratory outcomes included: days of any drug use and days of stimulant use in the past 30 days using the National Survey on Drug Use and Health^46^; opioid overdose risk behaviors in the past 30 days using the Opioid Overdose Risk Assessment^49^; OUD severity in the past 30 days using the Patient-Reported Outcomes Measurement Information System (PROMIS) severity of substance use disorder (adapted for OUD) T score^50^; and alcohol use severity using the AUDIT-Consumption (AUDIT-C).^51^

### Power and Sample Size

Original power and sample size estimates can be found in the trial protocol (Supplement 1).^19^ Post-hoc power calculations for the primary outcomes were for 80% power at a type I error rate of 1.25% to account for multiple outcomes. Calculations used the observed loss to follow-up rate (0%-43%) and distributions for our outcome variables from our sample. We had 80% power to detect: (1) a hazard ratio of at least 0.45 for our buprenorphine access measure; (2) 18.3 additional days of cumulative buprenorphine treatment within the first 180 days; (3) a 2.2-point reduction in depression symptoms (PHQ-9); and (4) a 6.2-point reduction in PTSD symptoms (PCL-5).

### Statistical Analysis

Missingness in our baseline data was less than 5%. We performed logical imputations and calculated scaled sums for composite measures. For all other missing items, we used mean imputation within treatment group and health system. We conducted descriptive analyses comparing characteristics by treatment group at baseline using a χ^2^ test (categorical) or a 2-sample *t* test (continuous). Data management and imputations were done in SAS, version 9.4 (SAS Institute). Bivariate comparisons, regression analyses and tables were done using Stata, version 17 (Stata Corp).

Analyses were performed in the intention-to-treat population, which consisted of all participants who were eligible for the outcome of interest. Response rates at 6 months ranged from 56% to 100%. For each outcome, we assessed representativeness of responders and determined if nonresponse weights were warranted (eTables 1 and 2 in Supplement 3). Due to imbalances on baseline covariates among responders, we used propensity score weights to ensure comparability, using the R twang package and including: health system; age; sex; race and ethnicity; education; marital status; prior MOUD; scores on baseline PHQ-9, PCL-5, PEG, and AUDIT; PROMIS t scores; mental and physical components from the 12-item Veterans RAND Health Survey; suicidal ideation; days of drug and stimulant use; recent overdose; housing status; legal trouble; and history of trauma.

Six-month outcomes were analyzed using cross-sectional weighted logistic, linear, and Cox proportional hazard models depending on the outcome distribution. Sensitivity to using different distributional assumptions was assessed for cases where normality did not appear to hold. Each model included propensity score and nonresponse weights, health system, prior MOUD exposure, and treatment assignment. Estimated effects were converted to adjusted means using recycled predictions for interpretation.

We also reported prespecified moderation analyses considering sex, ethnicity, stimulant use, trauma, housing, and pain across our 4 primary outcomes using models for each subsample of each moderator. All intention-to-treat and moderation hypotheses used 2-sided tests, with adjustment for multiple testing using the Benjamini-Hochberg procedure.^52^ Considering the null effects, we used instrumental variables to estimate the as-treated effects for participants who received an intake visit as well as for participants who received the key elements of collaborative care. In the instrumental-variable model, we controlled for health system, prior MOUD, age, sex, race and ethnicity, education, and marital status, and fit the models using ivreg in R, version 4.5.1 (R Foundation for Statistical Computing). Statistical significance was set to maintain a family-wise type I error rate of .05 with the given set of tests (eg, primary outcomes as a single class). Data analyses were performed from August 2024 to May 2025.

## Results

Of the 2396 individuals invited to complete the eligibility screener, 911 (38.0%) were eligible. We enrolled and analyzed 797 (87.5%) participants; 397 were assigned to EUC and 400 to collaborative care. The mean (SD) age of participants was 40.2 (11.9) years; 433 were female (54.3%) and 364 (45.7%) were male (Table 1). Regarding self-reported race, 41 were American Indian or Alaska Native (5.1%), 3 Asian (0.4%), 23 Black or African American (2.9%), 1 Native Hawaiian or Pacific Islander (0.1%), 514 White (64.5%), 148 other race (18.6%), and 67 more than 1 race (8.4%). Regarding ethnicity and other characteristics, 543 were Hispanic, Latino, or of Spanish origin (68.1%); 243 had less than a high school education (30.5%); 693 were housed (87.0%); and 154 were experiencing legal trouble (19.3%). The EUC and intervention groups were comparable at baseline (Table 1).

**Table 1.** Baseline Sociodemographic and Clinical Characteristics

Characteristic	Participants, No. (%)		
	Total	Enhanced usual care	Collaborative care
Participants, No.	797	397	400
**Sociodemographic characteristics**			
Age, mean (SD), y	40.2 (11.9)	40.3 (11.5)	40.0 (12.2)
Sex, per current birth certificate			
Female	433 (54.3)	213 (53.7)	220 (55.0)
Male	364 (45.7)	184 (46.3)	180 (45.0)
Race, self-reported^a^			
American Indian or Alaska Native	41 (5.1)	24 (6.0)	17 (4.2)
Asian	3 (0.4)	2 (0.5)	1 (0.2)
Black or African American	23 (2.9)	12 (3.0)	11 (2.8)
Native Hawaiian or Pacific Islander	1 (0.1)	1 (0.3)	0
White	514 (64.5)	250 (63.0)	264 (66.0)
Other race	148 (18.6)	71 (17.9)	77 (19.2)
More than 1 race	67 (8.4)	37 (9.3)	30 (7.5)
Hispanic, Latino, or Spanish origin, self-reported			
Yes	543 (68.1)	269 (67.8)	274 (68.5)
No	254 (31.9)	128 (32.2)	126 (31.5)
Education			
Less than high school	243 (30.5)	118 (29.7)	125 (31.2)
High school or equivalent	229 (28.7)	117 (29.5)	112 (28.0)
Some college or more	325 (40.8)	162 (40.8)	163 (40.8)
Marital status			
Never married	288 (36.1)	144 (36.3)	144 (36.0)
Married/living with partner	294 (36.9)	147 (37.0)	147 (36.8)
Widowed/divorced/separated	215 (27.0)	106 (26.7)	109 (27.2)
Living in stable housing (3 mo)	693 (87.0)	350 (88.2)	343 (85.8)
Any current legal trouble	154 (19.3)	69 (17.4)	85 (21.2)
**Clinical characteristics**			
Days with opioid use in past 30 d, mean (SD)	7.1 (11.5)	7.1 (11.4)	7.1 (11.6)
PROMIS OUD severity T score in past 30 d, mean (SD)^b^	53.2 (845)	53.1 (8.7)	53.2 (8.0)
Any opioid overdose events in past 3 mo	40 (5.0)	20 (5.0)	20 (5.0)
Days with stimulant use in past 30 d, mean (SD)	5.7 (10.1)	5.7 (10.3)	5.6 (10.0)
Days with any drug use in past 30 d, mean (SD)	9.4 (12.3)	9.5 (12.3)	9.3 (12.3)
AUDIT sum in past 3 mo, mean (SD)^c^	4.0 (7.6)	4.0 (7.7)	4.1 (7.5)
MOUD history in past 30 d			
No MOUD	152 (19.1)	73 (18.4)	79 (19.8)
Methadone as directed	147 (18.4)	72 (18.1)	75 (18.8)
Buprenorphine as prescribed	442 (55.5)	226 (56.9)	216 (54.0)
Taking MOUD, never/sometimes prescribed	56 (7.0)	26 (6.5)	30 (7.5)
PHQ-9 score, mean (SD)^d^	13.8 (5.73)	13.9 (5.82)	13.7 (5.65)
Any history of trauma^e^			
No trauma	64 (8.0)	27 (6.8)	37 (9.2)
Trauma, no interpersonal violence	432 (54.2)	214 (53.9)	218 (54.5)
Trauma with interpersonal violence	301 (37.8)	156 (39.3)	145 (36.2)
PCL-5 score, mean (SD)^f^	38.1 (16.9)	37.9 (16.4)	38.4 (17.3)
Suicidal ideation in past 30 d	241 (30.2)	127 (32.0)	114 (28.5)
VR-12 mental health component score, mean (SD)^g^	33.3 (12.7)	34.0 (12.5)	32.7 (12.9)
VR-12 physical health component score, mean (SD)^g^	37.4 (13.5)	38.1 (13.4)	36.7 (13.6)
PEG score, mean (SD)^h^	5.3 (3.0)	5.1 (3.0)	5.4 (2.9)
Health system identifier			
1	381 (47.8)	188 (47.4)	193 (48.2)
2	35 (4.4)	18 (4.5)	17 (4.2)
3	321 (40.3)	161 (40.6)	160 (40.0)
4	60 (7.5)	30 (7.6)	30 (7.5)
Probable diagnosis			
Depression only	150 (18.8)	68 (17.1)	82 (20.5)
PTSD only	176 (22.1)	90 (22.7)	86 (21.5)
Both	471 (59.1)	239 (60.2)	232 (58.0)

Abbreviations: AUDIT, Alcohol Use Disorders Identification Test; MOUD, medications for opioid use disorder; OUD, opioid use disorder; PCL-5, Post-Traumatic Stress Disorder Checklist for *DSM-5*; PEG, Pain, Enjoyment, General Activity tool; PHQ-9, Patient Health Questionnaire-9; PROMIS, Patient-Reported Outcomes Measurement Information System; PTSD, posttraumatic stress disorder; VR-12, Veterans RAND 12-Item Health Survey.

^a^ Participants were instructed to self-identify as other race if categories provided did not match their identity. Individuals endorsing more than 1 race category were assigned a value of more than 1 race.

^b^ PROMIS T score measures severity of opioid use disorder, scale: 0 (best) to 100 (worst), centered at a mean (SD) of 50 (10).

^c^ AUDIT sum measures severity of alcohol use, scale: 0 (best) to 40 (worst).

^d^ PHQ-9 measures severity of depression symptoms, scale: 0 (best) to 27 (worst).

^e^ History of trauma is coded using the worst traumatic event reported as part of the PCL-5 assessment. Events are categorized as trauma with interpersonal violence if they include harm intentionally inflicted on the participant by another person.

^f^ PCL-5 measures severity of PTSD symptoms, scale: 0 (best) to 80 (worst).

^g^ Mental health and physical health component scores measured with VR-12, scale: 0 (worst) to 100 (best), centered at a mean (SD) of 50 (10).

^h^ PEG measures severity of pain, scale: 0 (best) to 10 (worst).

**Figure 2.**
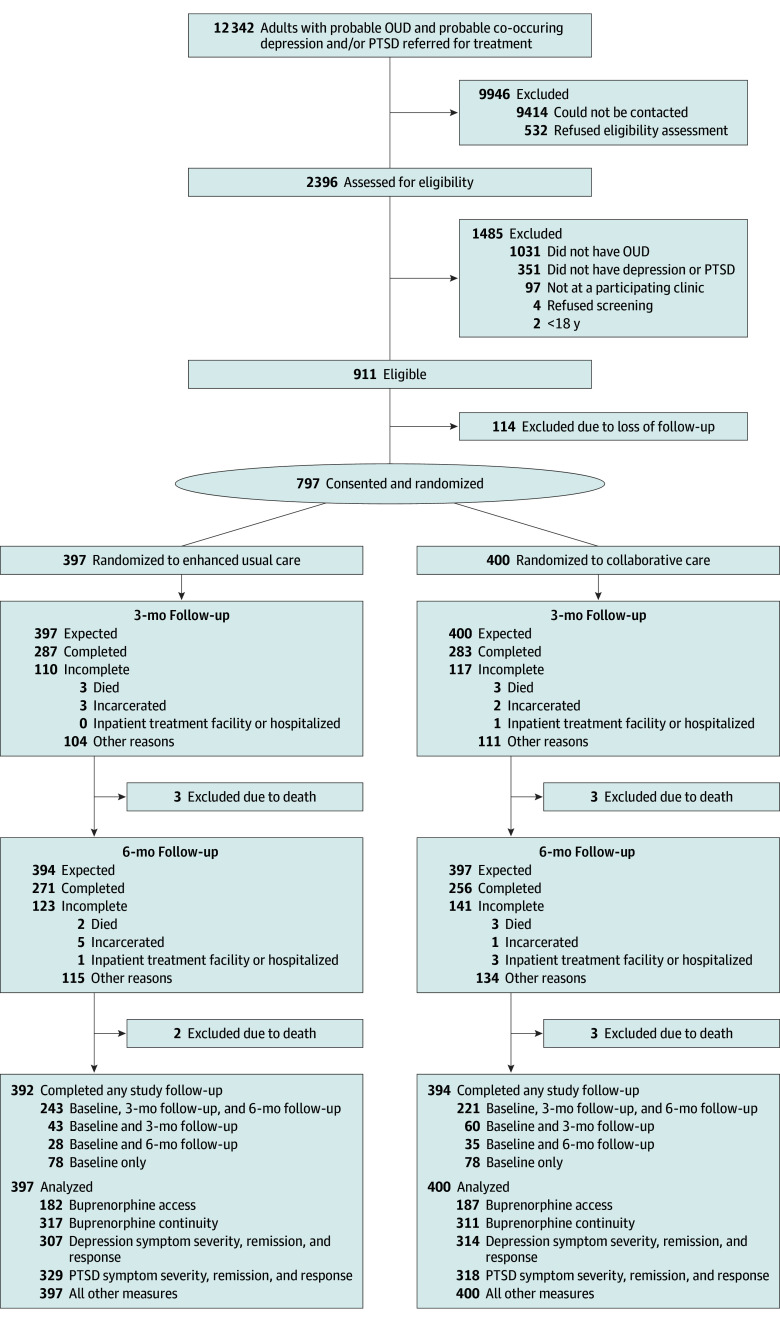
CLARO CONSORT Flow Diagram of Participants OUD indicates opioid use disorder; PTSD, posttraumatic stress disorder.

The 6-month follow-up survey was completed by 271 participants (68.2%) in the EUC group and 256 (64.0%) in the intervention group (Figure 2). Mean (SD) days of illicit opioid use in 30 days before enrollment was 7.1 (11.5) days; 40 participants (5.0%) reported an overdose in the past 3 months; 152 (19.1%) reported no MOUD in the 30 days before enrollment; mean (SD) PHQ-9 score was 13.8 (5.7) and mean (SD) PCL-5 score was 38.1 (16.9). Of the 797 total participants, 470 (59.0%) had all 3 disorders—OUD, major depression, and PTSD.

### Delivery of Collaborative Care Intervention

The intervention was delivered by 12 care managers. Of the 400 intervention participants 325 (81%) received an intake visit. Care managers made a mean (range) of 4 (0-38) unsuccessful contact attempts before the intake visit, which occurred a mean (SD) of 20 (29.5) days after randomization. During the 6-month period, 226 participants (56.5%) received the key elements of collaborative care, including a mean (median [range]) of 5.8 (5 [0-19]) total encounters with a mean (SD) session duration of 25.0 (14.4) minutes; they had a mean (SD) of 12 (9.5) unsuccessful contact attempts.

### Main Effects

Table 2 showcases the baseline means and follow-up adjusted means and treatment group differences for our primary, secondary, and exploratory outcomes, along with 95% CIs. Across all primary outcomes, there were no significant differences between study groups at 6 months, with the following adjusted mean (95% CI) differences of collaborative care vs EUC: 7.0 (95% CI, −3.4 to 17.4; *P* = .19) days until first buprenorphine prescription; 4.3 (95% CI, −7.4 to 16.0; *P* = .47) cumulative days of prescribed buprenorphine; −1.0 (95% CI, −2.3 to 0.3; *P* = .13) points on PHQ-9 score; and −0.9 (95% CI, −4.6 to 2.8; *P* = .63) points on PCL-5 score. We also did not observe any evidence of difference across study groups on any secondary or exploratory outcomes.

**Table 2.** Intent-to-Treat Model Results of Primary, Secondary, and Exploratory Outcomes at 6-Month Follow-Up, Along With Unadjusted Outcome Values at Baseline, By Treatment Group

Outcome	Participants, No.^a^		Baseline				6-mo Follow-up			
	Baseline	6 mo	Mean (95% CI)		Mean difference (95% CI)	*P* value	Adjusted mean (95% CI)			*P* value
			EUC	CC			EUC	CC	Adjusted mean difference (95% CI)	
**Primary outcomes**										
Days until first buprenorphine prescription^b^	369	369	NA	NA	NA	NA	20.2 (14.3 to 26.0)	27.1 (18.5 to 35.8)	7.0 (−3.4 to 17.4)	.19
Days of prescribed buprenorphine	628	604	NA	NA	NA	NA	89.0 (80.6 to 97.3)	93.3 (85.1 to 101.4)	4.3 (−7.4 to 16.0)	.47
PHQ-9 score^c^	621	405	15.8 (15.3 to 16.4)	15.7 (15.2 to 16.2)	−0.1 (−0.6 to 0.9)	.74	12.0 (11.1 to 12.9)	11.0 (10.1 to 11.9)	−1.0 (−2.3 to 0.3)	.13
PCL-5 score^d^	647	368	39.3 (37.5 to 41.1)	40.4 (38.6 to 42.2)	1.1 (−3.6 to 1.5)	.40	32.6 (30.1 to 35.1)	31.7 (29.0 to 34.4)	−0.9 (−4.6 to 2.8)	.63
**Secondary outcomes**										
MDD remission, %	621	405	NA	NA	NA	NA	12.8 (8.2 to 17.4)	18.3 (12.9 to 23.8)	5.5 (−1.7 to 12.7)	.13
MDD response, %	621	405	NA	NA	NA	NA	24.5 (18.5 to 30.5)	27.7 (21.4 to 34.0)	3.2 (−5.5 to 11.9)	.48
PTSD remission, %	647	368	NA	NA	NA	NA	57.6 (50.4 to 64.8)	56.1 (48.6 to 63.5)	−1.5 (−12.0 to 8.9)	.77
PTSD response, %	647	365	NA	NA	NA	NA	20.4 (14.5 to 26.3)	22.4 (16.2 to 28.6)	2.0 (−6.6 to 10.5)	.65
Suicidal ideation in past 30 d	797	523	32.0 (27.4 to 36.6)	28.5 (24.1 to 32.9)	−3.5 (−2.9 to 9.9)	.28	21.9 (16.7 to 27.1)	18.1 (13.3 to 22.9)	−3.8 (−10.9 to 3.3)	.29
Days with opioid use in past 30 d	797	523	7.1 (5.9 to 8.2)	7.1 (6.0 to 8.3)	0 (−1.7 to 1.5)	.92	6.1 (4.9 to 7.4)	6.3 (4.9 to 7.7)	0.2 (−1.8 to 2.0)	.89
Any opioid overdose events in past 3 mo	797	525	5.0 (2.9 to 7.2)	5.0 (2.9 to 7.1)	0 (0-0)	.98	3.7 (1.3 to 6.1)	5.1 (2.0 to 8.2)	1.4 (−2.6 to 5.3)	.49
VR-12 physical health component score^e^	797	527	38.1 (36.8 to 39.4)	36.7 (35.4 to 38.1)	−1.4 (−0.5 to 3.2)	.15	37.8 (36.3 to 39.4)	37.3 (35.7 to 38.9)	−0.5 (−2.8 to 1.7)	.62
VR-12 mental health component score^e^	797	527	34.0 (32.8 to 35.2)	32.7 (31.4 to 34.0)	−1.3 (−0.5 to 3.0)	.16	33.2 (31.7 to 34.7)	33.3 (31.8 to 34.9)	0.1 (−2.1 to 2.2)	.93
**Exploratory outcomes**										
Days with any drug use in past 30 d	797	525	9.5 (8.3 to 10.7)	9.3 (8.0 to 10.5)	−0.3 (−1.4 to 2.0)	.76	6.7 (5.4 to 8.1)	5.8 (4.4 to 7.1)	−0.9 (−2.8 to 1.0)	.34
Days with stimulant use in past 30 d	797	525	5.7 (4.7 to 6.7)	5.6 (4.7 to 6.6)	−0.1 (−1.3 to 1.5)	.91	4.2 (3.1 to 5.3)	3.9 (2.8 to 5.0)	−0.3 (−1.9 to 1.2)	.67
Opioid overdose risk behaviors score in past 30 d^f^	797	525	4.4 (3.8 to 5.0)	4.5 (3.9 to 5.1)	0.1 (−1.0 to 0.7)	.78	3.4 (2.7 to 4.1)	2.8 (2.2 to 3.5)	−0.6 (−1.5 to 0.4)	.23
PROMIS T score in past 30 d^g^	797	525	53.1 (52.2 to 54.0)	53.2 (52.4 to 54.0)	0.1 (−1.3 to 1.0)	.83	50.8 (49.8 to 51.7)	49.5 (48.5 to 50.5)	−1.3 (−2.6 to 0.1)	.07
AUDIT-C sum in past 3 mo^h^	797	522	NA	NA	NA	NA	1.7 (1.4 to 2.1)	1.4 (1.1 to 1.7)	−0.3 (−0.8 to 0.1)	.18

Abbreviations: AUDIT-C, Alcohol Use Disorders Identification Test-Consumption; CC, collaborative care; EUC, enhanced usual care; MDD, major depressive disorder; NA, not applicable; PCL-5, Post-Traumatic Stress Disorder Checklist for *DSM 5*; PHQ-9, Patient Health Questionnaire-9; PROMIS, Patient-Reported Outcomes Measurement Information System; PTSD, posttraumatic stress disorder; VR-12, Veteran’s RAND 12-Item Health Survey.

^a^ We report 2 sample sizes for each outcome since the eligibility for having difference outcomes varied by outcomes. First, we report the eligible group of potential participants. Then, we report the final number of responders who had 6-month outcomes among the eligible population. Follow-up rates for each outcome can be computed directly dividing eligible participants by respondents.

^b^ Results shown are from linear model; results from Cox proportional hazard model: EUC cumulative hazard (180 days), 0.59 (0.47 to 0.74); CC cumulative hazard (180 days), 0.70 (0.57 to 0.86); hazard ratio, 1.15 (0.85 to 1.55); *P* = .40. Unweighted medians were 12 and 13 for EUC vs CC, respectively.

^c^ PHQ-9 measures severity of depression symptoms, scale: 0 (best) to 27 (worst).

^d^ PCL-5 measures severity of PTSD symptoms, scale: 0 (best) to 80 (worst).

^e^ Mental health and physical health component scores measured with VR-12, scale: 0 (worst) to 100 (best), with mean (SD) of 50 (10).

^f^ Opioid overdose risk behaviors score measures behaviors associated with risk of opioid overdose, scale: 0 (best) to 34 (worst).

^g^ PROMIS T score measures severity of opioid use disorder, scale: 0 (best) to 100 (worst), with mean (SD) of 50 (10).

^h^ AUDIT-C sum measures severity of alcohol use, scale: 0 (best) to 12 (worst).

eTable 3 in Supplement 3 shows that the as-treated analyses found statistically significant evidence of effectiveness for only 2 exploratory outcomes: opioid overdose risk behaviors and OUD severity; although statistically significant, these effects correspond to small Cohen *d* effect sizes of −0.18 (95% CI, −0.35 to −0.01) to −0.28 (95% CI, −0.52 to −0.04), respectively.

### Moderation Analyses

**Figure 3.**
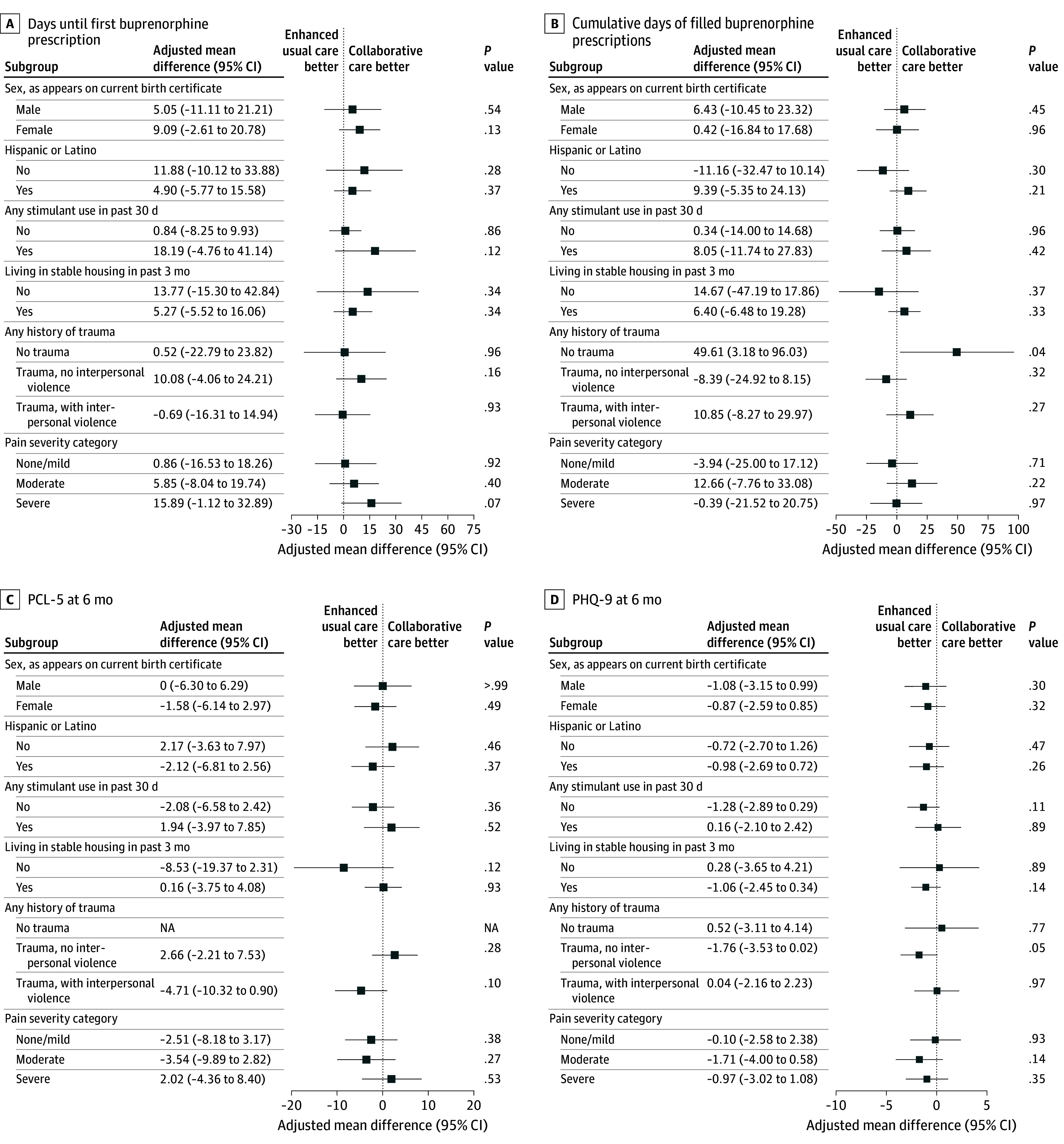
Moderation Analysis Results of the Effect of Collaborative Care (CC) vs Enhanced Usual Care (EUC) on the Primary Outcomes Time to event is reported in days. All models are weighted for nonresponse and group imbalance and control for health system. The no trauma group is not applicable for the PCL-5 outcome because the PCL-5 is not administered to participants with no traumatic event. For ease of interpretation, we present results of linear models for days until first buprenorphine prescription. We also ran Cox proportional hazard models for each moderator, and no results were statistically significant. The Pain, Enjoyment and General Activity^37^ scale was used to measure pain. NA indicates not applicable; PCL-5, Post-Traumatic Stress Disorder Checklist for *DSM-5; *PHQ-9, Patient Health Questionnaire-9.

Figure 3 shows findings from the moderation analysis. After adjusting for multiple testing, there was no evidence of moderation between the factors considered and our study’s primary outcomes.

## Discussion

In this randomized clinical trial, the primary analysis found no evidence of an impact of being assigned to a multicomponent collaborative care intervention for participants with OUD and co-occurring depression and/or PTSD on any of the primary, secondary, or exploratory outcomes; both groups improved. While the as-treated analyses showed statistically significant effects on reduced opioid overdose risk behaviors and OUD severity, these findings are of uncertain clinical significance given the small effect sizes observed. Moderator analyses also did not show any effect. With careful consideration, these findings can still inform the management of OUD with co-occurring mental illness in primary care settings with few behavioral health resources.

Our findings are consistent with null findings from 2 other studies of collaborative care for people with drug use disorders.^21,53^ First, the AHEAD trial^53^ showed no improvement in self-reported abstinence from opioids, stimulants, or heavy drinking. Second, the SUMMIT trial^21^ for individuals with either alcohol or OUD showed improvement in abstinence from opioids and/or alcohol, but the results were associated primarily with reductions in alcohol use.^54^ More recently, the CHAMP trial,^55^ using a cluster randomized design, showed statistically significant improvement in days of opioid use for participants assigned to collaborative care for both OUD and mental illness compared to participants who received only collaborative care for mental illness. While this is promising, the patient population enrolled in the trial had a less severe clinical profile. Thus, collaborative care for drug use disorders may not be effective for more patients with more clinically complex issues or may be more difficult to implement.

The lack of evidence of an impact on mental health outcomes is more surprising. The effectiveness of collaborative care for mental health disorders is well established (although lower for Medicaid-insured populations),^56,57,58^ and care managers were able to engage most patients.^36^ It is possible that the training and implementation support provided benefitted participants in both groups of the trial, representing spillover effects that impacted both mental health and OUD outcomes for all patients. Some individuals in the EUC group may have received care coordination from community health workers not associated with the study or psychotherapy from study-trained behavioral health practitioners. The magnitude of improvement in depression scores was similar to other trials of collaborative care,^59,60,61,62^ and both groups also significantly decreased days of drug use and OUD symptom severity between baseline and month 6. Qualitative data indicate that during the course of the study, practitioners became more comfortable treating co-occurring disorders.^63^

There are other possible explanations that warrant consideration. First, the primary care population enrolled in the trial was clinically complex, with higher levels of symptom severity and adverse social determinants of health than is typical for collaborative care trials; 59% met criteria for all 3 disorders. Compared to other studies of multimorbid conditions showing positive outcomes, baseline depression scores in our study were as high or higher, and some patients were co-using stimulants, unhoused, or justice-involved, which are all associated with worse outcomes.^59,60,61,62,64,65^ Second, most clinics were in location identified as MHPSAs and did not have on-site behavioral health practitioners, which likely posed a barrier to psychotherapy. Third, while our trial tailored collaborative care to address workforce shortages by using community health workers as care managers, the role of community health workers in the US has been primarily to provide auxiliary support to mental health treatment.^66^ As such, the care managers did not provide psychotherapy, which may have limited clinical impact. Previous research suggests psychotherapy may be a critical component of collaborative care for depression.^67^ These factors could have limited the ability of collaborative care to produce benefits beyond those of EUC.

Lastly, implementation challenges may have limited impact. The trial was launched at the beginning of the COVID-19 pandemic; most care managers worked remotely, which may have impacted their ability to support integrated care. By design, care managers addressed social determinants of health; for some patients and care managers this may have taken priority over behavioral health treatment. Moreover, none of the clinics provided collaborative care before the study. Despite the substantial level of support provided, it is possible implementation of some components of the model was suboptimal.

### Strengths and Limitations

Strengths of our study include the randomized design, a high proportion of Hispanic participants, standardized enrollment criteria with few exclusions, masked outcomes assessment, and the use of Prescription Drug Monitoring Program data as the source of buprenorphine outcomes. The study trained community health workers without previous clinical experience as a way of addressing practitioner shortages. In other research we have reported that both patients and practitioners found collaborative care acceptable and feasible.^63,68^

This study has several limitations. First, we did not rely on a psychiatric interview to determine diagnoses, and we excluded individuals who were actively suicidal or psychotic. Many of the participants entered the study already taking MOUD, which limited power for assessing 1 of our primary outcomes. Although we used a propensity−weighted analysis to address loss to follow-up, almost one-third of study participants did not have a 6-month follow-up assessment. The study was conducted primarily in clinics in MHPSAs; results may not generalize to clinics in communities with more behavioral health resources. We do not know whether participants in both groups of the trial received psychiatric medication or psychotherapy. Because practitioners cared for participants in both groups, spillover could have occurred, although we mitigated this by clearly defining the care manager role, masking patient assignment to EUC for practitioners, and ensuring that psychiatric consultants did not care for EUC patients. While a cluster randomized trial could have minimized spillover, cluster trials are associated with recruitment bias and need larger samples.^69^ There was no follow-up beyond month 6; a longer intervention period may have shown benefit.

## Conclusions

In this randomized clinical trial, adults with OUD co-occurring with depression and/or PTSD who received a multicomponent collaborative care intervention did not show evidence of improved OUD or mental health outcomes compared with EUC; both groups improved. Before drawing conclusions, future research is needed to assess whether the lack of impact was due to the way collaborative care was tailored for patients with clinically complex issues and/or settings with few behavioral health resources, problems with implementation, and/or spillover or spontaneous improvement. In addition, how to provide psychotherapy in settings with few behavioral health practitioners remains an unanswered question.

## Notes

Supplement 1. Trial Protocol

Supplement 2. Statistical Analysis Plan

Supplement 3. eTable 1. Baseline Characteristics by Response Status and Weighting for MDD Symptom Severity Outcome eTable 2. Baseline Characteristics by Response Status and Weighting for PTSD Symptom Severity Outcome eTable 3. As-Treated Adjusted Effect Estimates for Primary, Secondary, and Exploratory Outcomes

Supplement 4. Data Sharing Statement
