# Supplementary material for: Collaborative Care for Opioid Use Disorder and Mental Illness: The CLARO Randomized Clinical Trial
Source: JAMA Intern Med. 2025 Dec 29;186(2):168–80. doi: 10.1001/jamainternmed.2025.7036 (PMC12750335; doi:10.1001/jamainternmed.2025.7036)
Supplement: Supplement 1. — Trial Protocol [file jamainternmed-e257036-s001.pdf]

Protocol for Randomized Controlled Trial Comparing Collaborative Care and Enhanced Usual Care for Patients with Co-occurring Opioid Use Disorders and Depression and/or Posttraumatic Stress Disorder

National Clinical Trial (NCT) Identified Number: NCT04559893

Principal Investigators: Katherine E. Watkins and Miriam Komaromy (Multiple Principal Investigators)

RAND Corporation and Boston Medical Center

Grant Title: Improving Access and Treatment for Co-occurring Opioid Use Disorders and Mental Illness

Grant Number: U01 MH 121954

Funded by: NIMH

Version Number: 13.1

November 15, 2024

Summary of Changes from Previous Version:

**Affected Section(s)**	**Summary of Revisions Made**	**Rationale**
**Throughout Protocol**	Revised analysis and outcomes to correspond with Statistical Analysis Plan updates from November 2024, which was submitted and approved by our IRB. This included changing the operationalization of the primary outcomes, and combining the parent study and CR populations for all analyses.	Ensure all design and analysis descriptions reflect latest knowledge and assumptions (e.g., actual sample size, best practices, data limitations, resource constraints, etc.) prior to unblinding

CONFIDENTIALITY STATEMENT

This document is confidential communication. Acceptance of this document constitutes agreement by the recipient that no unpublished information contained herein will be published or disclosed without prior approval of the Principal Investigator or other participating study leadership and as consistent with the NIH terms of award.

Table of Contents

STATEMENT OF COMPLIANCE 1
INVESTIGATOR'S SIGNATURE 2
1 PROTOCOL SUMMARY 3
1.1 Synopsis 3
1.2 Schema 7
1.3 Schedule of Activities 9
2 INTRODUCTION 11
2.1 Study Rationale 11
2.2 Background 12
2.3 Risk/Benefit Assessment 17
2.3.1 Known Potential Risks 17
2.3.2 Known Potential Benefits 19
2.3.3 Assessment of Potential Risks and Benefits 19
3 OBJECTIVES AND ENDPOINTS 19
4 STUDY DESIGN 22
4.1 Overall Design 23
4.2 Scientific Rationale for Study Design 25
4.3 Justification for Intervention 25
4.4 End-of-Study Definition 27
5 STUDY POPULATION 27
5.1 Inclusion Criteria 5
5.2 EXclusion Criteria 5
5.3 Screen Failures 5
5.4 Strategies for Recruitment and Retention 5
6 STUDY INTERVENTION 14
6.1 Study InterventionAdministration 14
6.1.1 Study Intervention Description 15
6.1.2 Administration 18
6.2 Fidelity 18
6.2.1 Interventionist Training and Tracking 18
6.3 Measures to Minimize Bias: Randomization and Blinding 20
6.4 Study Intervention Adherence 21
7 STUDY INTERVENTION DISCONTINUATION AND PARTICIPANT DISCONTINUATION/WITHDRAWAL 21
7.1 Discontinuation of Study Intervention 21
7.2 Participant Discontinuation/Withdrawal from the Study 22
7.3 Lost to Follow-Up 22
8 STUDY ASSESSMENTS AND PROCEDURES 23
8.1 Endpoint and Other Non-Safety Assessments 23
8.2 Safety Assessments 27
8.3 Adverse Events and Serious Adverse Events 27
8.3.1 Definition of Adverse Events 27
8.3.2 Definition of Serious Adverse Events 28
8.3.3 Classification of an Adverse Event 28

8.3.4 Time Period and Frequency for Event Assessment and Follow-Up......................30
8.3.5 Adverse Event Reporting 30
8.3.6 Serious Adverse Event Reporting 31
8.3.7 Reporting of Pregnancy 33
8.4 Unanticipated Problems..33
8.4.1 Definition of Unanticipated Problems 33
8.4.2 Unanticipated Problems Reporting..33
8.4.3 Reporting Unanticipated Problems to Participants 34
9 STATISTICAL CONSIDERATIONS 34
9.1 Statistical Hypotheses..34
9.2 Sample Size Determination..35
9.3 Populations for Analyses 37
9.4 Statistical Analyses..37
9.4.1 General Approach..37
9.4.2 Analysis of the Primary Endpoint(s) 37
9.4.3 Analysis of the Secondary Endpoint(s)..38
9.4.4 Baseline Descriptive Statistics 39
9.4.5 Sub-Group Analyses 39
9.4.6 Exploratory Analyses..39
10 SUPPORTING DOCUMENTATION AND OPERATIONAL CONSIDERATIONS 39
10.1 Regulatory, Ethical, and Study Oversight Considerations..39
10.1.1 Informed Consent Process 39
10.1.2 Study Discontinuation and Closure 40
10.1.3 Confidentiality and Privacy 41
10.1.4 Future Use of Stored Specimens and Data 44
10.1.5 Key Roles and Study Governance 44
10.1.6 Safety Oversight..46
10.1.7 Clinical Monitoring..46
10.1.8 Clinical monitoring will be performed by the independent NIMH Data Safety and Monitoring Board (DSMB) and the RAND site. Quality Assurance and Quality Control 46
10.1.9 Data Handling and Record Keeping..47
10.1.10 Protocol Deviations..48
10.1.11 Publication and Data Sharing Policy 48
10.1.12 Conflict of Interest Policy 49
10.2 Abbreviations and Special Terms 49
10.3 Protocol Amendment History 52
11 REFERENCES 56

STATEMENT OF COMPLIANCE

The trial will be carried out in accordance with International Council on Harmonization Good Clinical Practice (ICH GCP) and the following:

- United States (US) Code of Federal Regulations (CFR) applicable to clinical studies (45 CFR Part 46, 21 CFR Part 50, 21 CFR Part 56, 21 CFR Part 312, and/or 21 CFR Part 812).

National Institutes of Health (NIH)-funded investigators and clinical trial site staff who are responsible for the conduct, management, or oversight of NIH-funded clinical trials have completed Human Subjects Protection and ICH GCP Training.

The protocol, informed consent form(s), recruitment materials, and all participant materials will be submitted to the IRB for review and approval. Approval of both the protocol and the consent form(s) must be obtained before any participant is consented. Any amendment to the protocol will require review and approval by the RAND IRB – the Human Subjects Protection Committee (HPSC) -- before the changes are implemented to the study. All changes to the consent form(s) will be IRB approved; a determination will be made regarding whether a new consent needs to be obtained from participants who provided consent, using a previously approved consent form.

INVESTIGATOR'S SIGNATURE

The signature below constitutes the approval of this protocol and provides the necessary assurances that this study will be conducted according to all stipulations of the protocol, including all statements regarding confidentiality, and according to local legal and regulatory requirements and applicable US federal regulations and ICH guidelines.

Principal Investigator or Clinical Site Investigator:

Signed: ______________________________ Date: ______________

Name: Katherine E. Watkins (MPI)

Title: Senior Physician Policy Researcher

Investigator Contact Information
Affiliation: RAND Corporation
Address: 1776 Main Street, Santa Monica, CA 90407-2138
Telephone: 310.393.0411, x6509
Email: kwatkins@rand.org

[For multi-site studies, the protocol should be signed by the clinical site investigator who is responsible for the day to day study implementation at his/her specific clinical site.]

Signed: ______________________________ Date: ______________

Name: Miriam Komaromy, MPI

Title: Medical Director, Grayken Center for Addiction

Affiliation: Boston Medical Center

Signed: ______________________________ Date: ______________

Name: Valerie Carrejo MD

Title: Co-Investigator

Affiliation: University of New Mexico Health Sciences Center

1 PROTOCOL SUMMARY

SYNOPSIS

Title: Improving Access and Treatment for Co-occurring Opioid Use Disorders and Mental Illness

Grant Number: U01 MH 121594

Study Description: This is a multi-site, randomized pragmatic trial in rural and urban primary care clinics located in New Mexico and California to adapt, harmonize and then test whether CC-COD improves access, quality and outcomes for primary care patients with co-morbid OUD and depression and/or PTSD. We will randomize patients with co-occurring OUD and depression and/or PTSD to receive either CC-COD or enhanced usual care (EUC).

Summary of Competitive Revision (CR): The CR develops and then tests an enhanced version of the parent study's collaborative care intervention for co-occurring disorders (CC-COD) to reduce the risk of suicide and opioid-related overdose among individuals with opioid use disorder (OUD) in combination with PTSD/depression. Within each clinic, once enrollment into the parent study has been completed (N=900), clinics will transition to delivering CC-COD+ and patients (N=300) will be randomized to either CC-COD+ or EUC. All study procedures and measures are identical for both the parent study and the CR. Because the CR did not enroll sufficient individuals to allow for separate analyses, it was decided to combine patients enrolled into the parent study intervention arm, with patients enrolled into the CLARO+ study intervention arm for all analyses. This was discussed and approved by our Research Advisory Board.

Hypothesis 1: Patients randomly assigned to CC-COD/CC-COD+ will have significantly increased buprenorphine access and quality of care, and depression and PTSD outcomes compared to patients assigned to EUC.

Hypothesis 2: Patient experiences will mediate the effect of CC-COD on quality and outcomes compared to patients assigned to EUC.

**Objectives:**	Primary Objective: To evaluate the effectiveness of CC-COD/CC-COD+ on patient initiation of MOUD, quality of care for OUD; and depression and PTSD patient outcomes relative to EUC.<br><br>Secondary Objectives: To test mediators (patient experiences with care and working alliance with the Care Coordinator) of treatment quality and patient-reported outcomes and, in exploratory analyses, test moderators of access, quality, and outcomes compared with patients assigned to EUC.
**Endpoints:**	Primary Endpoints: Our primary outcomes are buprenorphine continuity of care, buprenorphine access, MDD symptom severity, and PTSD symptom severity.<br>Secondary Endpoints: Secondary outcomes include opioid use frequency, opioid overdose events, suicidality, access to MDD and/or PTSD treatment, quality of care for MDD, quality of care for PTSD, MDD remission, MDD response, PTSD remission, PTSD response, physical health functioning, and mental health functioning.
**Study Population:**	The parent study will recruit 900 women and men adult patients with OUD and either depression or PTSD from 14 clinics in three large healthcare organizations that provide care throughout the state of New Mexico, and 7 clinics in two large healthcare organizations that provide care throughout Los Angeles County in California. The 7 clinics in California were collapsed into four sites because of geographical proximity and shared providers.<br><br>Patients in these settings are mostly low-income, predominantly Hispanics in HPSAs for primary care, mental healthcare, or both.<br><br>The CR will recruit 300 women and men adult patients with OUD and either depression or PTSD from 10 clinics in three large healthcare organizations that provide care throughout the state of New Mexico and 7 clinics in two large healthcare systems that provide care throughout Los Angeles County in California.<br>Because the CR did not enroll sufficient individuals to allow for separate analyses, in consultation with our Research Advisory Board, the NIMH scientific officer and the NIMH project officer, it was decided to combine patients enrolled into the parent study intervention arm, with patients enrolled into the CLARO+ study intervention arm for all analyses.

**Phase:**	N/A; This trial and the CR tests the effectiveness of a service delivery intervention. The service delivery intervention includes the organized delivery of multiple evidence-supported medications, psychosocial treatments, and care management approaches that are or could be furnished as part of routine care for the target population.
**Description of Sites/Facilities Enrolling Participants:**	For both the parent trial and the CR, RAND will collaborate with the University of New Mexico Health Sciences Center (UNM HSC), and leverage relationships with sites from First Choice Health Care (FCCH) system in New Mexico; UNM's Southeast (SE) Heights Health Center, Southwest Mesa and North Valley and Sandoval Regional Medical Center (SRMC); Hidalgo Medical Services (HMS) in New Mexico; Providence Health & Services – Southern California; and the Los Angeles County Department of Health Services under the auspices of the Olive View Education and Research Institute, also in Los Angeles, California. There are multiple sites that are covered under our agreements with the Olive View Education and Research Institute, including the Hubert Humphrey Comprehensive Health Center, Mid Valley Comprehensive Health Center, and San Fernando Health Center (which is comprised of Glendale Health Center, West Valley Health Center, and San Fernando Health Center).

**Description of Study Intervention/Experimental Manipulation:**	The intervention is an adaptation of Collaborative Care (CC) for use in resource-poor communities and for patients that have co-occurring disorders (CC-COD). It is a service delivery approach that uses multi-faceted interventions to improve access and quality of care. Essential members of the CC-COD team include the Care Coordinator, primary care provider (PCP), Behavioral Health Provider (BHP) and Behavioral Health Consultant (BHC). Essential activities of the CC-COD model include population-based care supported by a clinical registry, the use of measurement-based care and treat-to-target practices, shared decision-making and expert consultation by a team of BHCs with complementary expertise. All of these activities are facilitated by the Care Coordinator.<br><br>CC-COD will also include access to supported treatments: MOUD, medication treatment for depressive disorders and/or PTSD, motivational interviewing, problem-solving therapy (PST) for depression, and Written Exposure Therapy (WET) for PTSD. The intervention will run for 36 months across patients but for six months per individual patient. The initial 45-minute patient contact with the intervention is intended to be face-to-face in the clinic (but could be virtual depending on the level of restrictions related to COVID-19); subsequent intervention contacts will be 15-minute telephone calls or in-person contacts, and, as needed, through community outreach visits.<br><br>CC-COD+ will add three additional components to CC-COD. Care coordinators will (1) educate family members about MOUD with the goal of increasing patient retention in treatment; (2) train a support persons (e.g., a family member, friend, or partner) and the patient to administer naloxone and on how to reduce opioid-overdose risk behaviors; and (3) implement Caring Contacts, a suicide prevention intervention that sends compassionate mailed or text messages to individuals to decrease social isolation and reduce suicide risk.

**Study Duration:**	The study intends to enroll its first patient approximately 13 months from the study open (month 14, in November 2020). Recruitment is planned for 24 months with an additional six months to account for any recruitment problems, with baseline data collection completed by the middle of year 4 (May 2023) and both 3- and 6-month data collection completed by early year 5 (December 2023). The study will begin with recruitment at the New Mexico clinics in early year 2 (late 2020), as described above. The study intends to begin recruitment at the California clinics at the end of year 3 (summer of 2022). Mid-Valley Comprehensive Health Center and San Fernando Health Center clinics will be added in the fall/winter of 2022. We anticipate concluding recruitment in December 2023 with both 3- and 6-month data collection completed by June 2024.<br><br>The CR intends to enroll its first patients in month 26 (September 2022) and is expected to continue data collection activities until June 2024.
**Participant Duration:**	Clinic staff will participate in data collection prior to the intervention (during months 10-15) and after the intervention is complete (months 29-31). Patient participants for both the parent and the CR will be involved in the study for six months.

SCHEMA

Estimated Flow Diagram for Randomized Controlled Trial: Collaborative Care for Co-occurring Opioid Use Disorders and Depression and/or PTSD

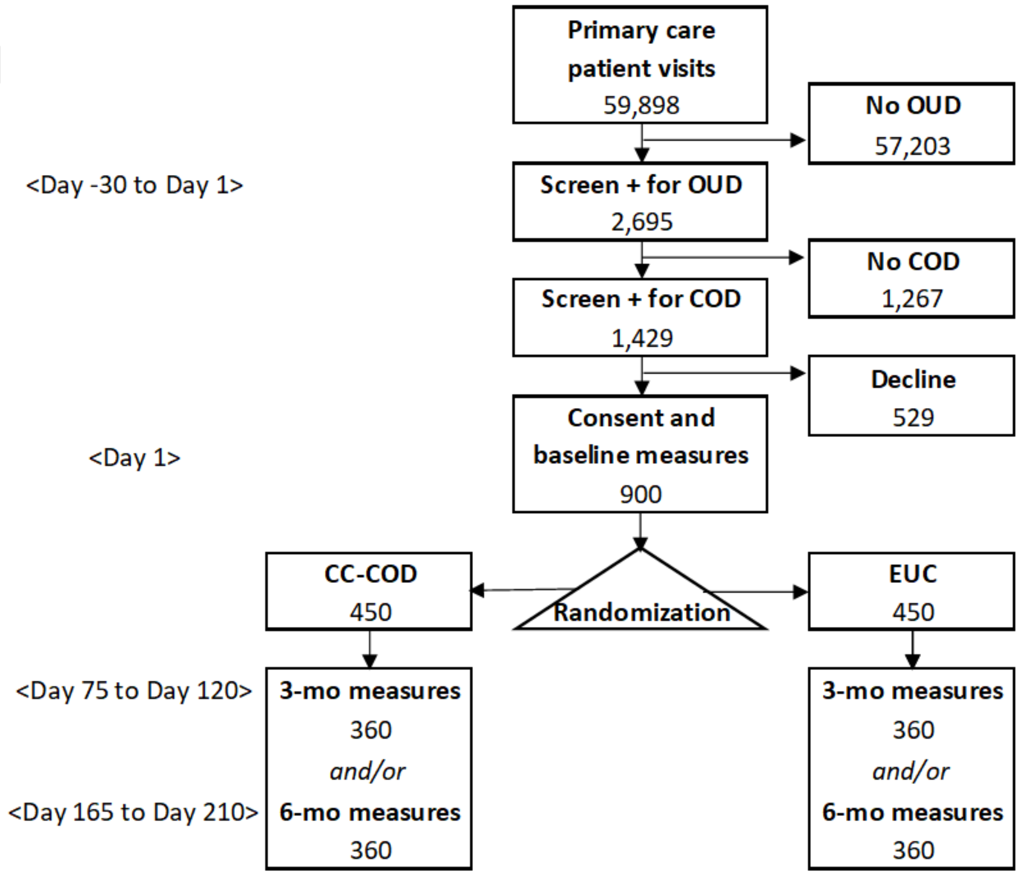

Note. OUD = opioid use disorder. COD = co-occurring disorder (Major Depressive Disorder and/or Post-Traumatic Stress Disorder). CC-COD = Collaborative Care for COD. EUC = Enhanced usual care. Baseline, 3-month, and 6-month measures, data analyses, and CC-COD intervention activities are detailed in **1.3. Schedule of Activities**.

SCHEDULE OF ACTIVITIES

	Eligibility screening (Pre-consent)	Baseline Day 1	3-Mo Follow-up Day 90 -14/+30	6-Mo Follow-up Day 180 -14/ +30
Probable OUD diagnosis (NIDA myTAPS[1] or past 90 day receipt of MOUD)	X			
Patient health questionnaire (PHQ-9) for depression[2]	X			
Probable PTSD (PC-PTSD-5)[3]	X			
Age 18 or older	X			
Patient at participating clinic	X			
Current receipt of both MOUD and psychotropic medication from provider(s) outside of the participating primary care health system (exclusion criterion)	X			
Speak English or Spanish	X			
Informed consent	X			
Randomization		X		
Adverse event reporting		X	X	X
Outcome Evaluation				
MOUD continuity of care[4] (max number of continuous (i.e., no breaks of more than 7 days) days on MOUD; EHR review)			X	X
MOUD access (receipt of MOUD prescription within 30 days of diagnosis; EHR review)[5]		X	X	X
MDD symptoms (PHQ-9[2] raw score change)		X	X	X
PTSD symptoms (PCL-5 raw score change)		X	X	X
Drug use frequency (NSDUH[6] items)		X	X	X
Opioid use severity (PROMIS Substance Use Short Form[7])		X	X	X
Alcohol use (AUDIT-C[8])*			X	X
Opioid overdose risk behaviors (Opioid Overdose Risk Assessment[9])		X	X	X
Opioid overdose events (Naloxone Overdose Baseline Questionnaire[10])		X	X	X
MDD remission (PHQ-9[2] score < 10)		X	X	X
MDD response (PHQ-9[2] score < 50% of baseline)		X	X	X
PTSD remission (PCL-5 score < 34)		X	X	X
PTSD response (PCL-5 score < 50% of baseline)		X	X	X
Suicidality (Columbia Suicide Severity Rating Scales[11])		X	X	X
All-cause mortality (death records)			X	X
MOUD initiation[12] (receipt of MOUD prescription within 14 days of diagnosis; EHR review)			X	X
MOUD engagement[12] (receipt of two or more MOUD prescriptions within 34 days of diagnosis; EHR review)			X	X
Access to MDD and/or PTSD treatment (receipt of medication and/or behavioral treatment associated with diagnosis; EHR review)			X	X
Quality of care for MDD[13] (4 psychotherapy visits in the first 8 weeks or an adequate (12-week) medication trial)			X	X
Quality of care for PTSD[13] (4 psychotherapy visits in the first 8 weeks or an adequate (60-day) medication trial)			X	X

Veterans RAND 12-item General health functioning (VR-12[14])		X	X	X
Demographics (sex, race, ethnicity, education level, marital status)		X		
Alcohol use severity (AUDIT[8])		X		
Pain levels (PEG Pain Monitor[15])		X	X	X
History of MOUD treatment (items developed by research team)		X		
Current MDD/PTSD treatment (NSDUH[6] items)		X		
Prior experiences with Care Coordinator (items developed by research team)		X		
Interpersonal support (presence of a support person who does not have problematic opioid use)		X		
Homelessness (Homelessness Screening Clinical Reminder Tool;[16] GPRA[17]** item)		X	X	X
Legal involvement (NSDUH items; Addiction Severity Index[18] items)		X	X	X
Disability and impairment (Sheehan Disability Scale[19])		X		
Rurality (Rural-Urban Commuting Area code[20] associated with 5-digit ZIP code)		X		
Care Coordinator communication (AHRQ Consumer Assessment of Healthcare Providers and Systems[21])			X	
Ability to access treatment quickly (AHRQ CAHPS[21])			X	
Satisfaction with treatment (AHRQ CAHPS[21])			X	
Patient-Care Coordinator working alliance (Modified form of Working Alliance Inventory – General Practice[22])			X	

*The full AUDIT will be administered at baseline.
**Government Performance Results and Modernization Act of 2010.

2 INTRODUCTION

STUDY RATIONALE

Problem/question. Untreated mental illness and substance use disorders are common and can have devastating consequences for the individual, their families and the community. Co-occurring opioid use disorders (OUD) with either depressive disorders and/or posttraumatic stress disorder (PTSD) are particularly concerning because of their prevalence in people with OUD. Co-occurring mental illness is also linked to an increased risk for opioid misuse and overdose, and because of the high prevalence of the chronic use of prescription opioids in individuals with mental illness, a risk factor for heroin use and the development of an OUD. Primary care is an important and underutilized setting in which to provide treatment for all three disorders, because OUD, depression and PTSD are frequently co-morbid with medical conditions. However, despite the effectiveness of treatments for all three disorders, many individuals never receive treatment; and, when treatment is provided, quality is low. With the rising number of opioid-related fatalities, this is a critical treatment and quality gap in a vulnerable and stigmatized population.

Rationale for clinical trial and intervention choice. Collaborative care (CC) has potential to address these problems by improving access, quality and outcomes in primary care patients with common mental health (MH) conditions. However, CC has never been tested with co-occurring disorders (COD), despite research by our team suggesting it may be effective. This study will compare CC for COD (CC-COD) with enhanced usual care (EUC) on study outcomes (described elsewhere). In addition to examining the effectiveness of the CC-COD intervention, this study will test whether intervention targets (patient experiences with care and working alliance with the Care Coordinator) mediate the effect of CC-COD on quality and outcomes compared to participants randomized to EUC.

Problem/question for CR. Deaths from suicide and overdose related to opioid use are a rapidly growing and major public health crisis.[23] In 2017, more than 40% of suicide and overdose deaths were linked to opioid use, and opioid use disorders (OUD) have a strong relationship to both suicide and overdose.[24-30] Similarly, mental health conditions are also linked to suicide and unintentional overdose.[31,32] It is no surprise that individuals with these co-occurring disorders (COD) are at higher risk of attempting suicide and dying from suicide or overdose[31-34] than individuals with OUD or mental illness alone. In one study, 67% of those with co-morbid OUD and major depression had attempted suicide in the past year, along with 47% of those with comorbid OUD and PTSD.[35] OUD, depression, and PTSD commonly occur together with devastating consequences.[36-42]

Medication for OUD (MOUD) reduces the risk of suicide attempts and unintentional overdose, and buprenorphine specifically is associated with a decrease in severe suicidal ideation among individuals with comorbid OUD and major depression.[43,44] Yet despite the importance of MOUD, many people with OUD and COD never receive treatment, and 50–80% of those who do initiate MOUD discontinue treatment, often within weeks or months of initiation,[45-48] putting them at increased risk of relapse and overdose. Individuals are particularly vulnerable to suicide and overdose immediately after discontinuing MOUD, with comorbidity adding further risk.[48] Rates of overdose are approximately 2-3 times higher after MOUD is discontinued than rates among individuals who never received treatment

for OUD at all. Treatment for depression and PTSD is also associated with less suicidality and mortality, but access is low, particularly for the COD population.[49-51]

Rationale for CR. The parent study is among the first to adapt CC for co-occurring OUD, depression, and/or PTSD (CC-COD) and then test it in a randomized controlled trial with the goal of improving access, quality and patient outcomes. However, while the parent study includes measures of suicide and overdose as outcomes, the evidence-based interventions that our CC-COD model supports do not specifically address suicide risk, overdose prevention or naloxone training. In addition, CC-COD does not include outreach to support persons (e.g., family, friends, or partners), even though increasing evidence from our team and others suggests that support persons (SPs) can influence access to and retention in substance use treatment.[52-61] In our proposed revision, we expand the scope of the parent grant to address these limitations and the public health crisis. The CR will develop and then test an enhanced version of the parent study's collaborative care intervention for co-occurring disorders (CC-COD+) to reduce the risk of suicide and opioid-related overdose among individuals with opioid use disorder (OUD) in combination with PTSD/depression.

BACKGROUND

Opioid use disorders, co-occurring with either depression or PTSD, are prevalent, burdensome, and too often left untreated. In 2015-16, there were over two million adults with a current OUD; 62% had a co-occurring mental illness and 24% had a co-occurring serious mental illness.[34] While individuals with COD are more likely to receive MH treatment than OUD treatment, only 16-25% report receiving treatment for both conditions.[34,62] Depression and PTSD are two of the most common MH co-morbidities in people with OUD, and when present, are associated with poorer outcomes.[38,39,41,63-68] Both depression and PTSD are leading causes of disability, and mortality from OUD continues to rise.[69,70]

Primary care is an important and underutilized setting in which to identify and provide treatment, but utilization and quality of OUD and behavioral health care is low.[71-73] Although specialty OUD care plays a critical role for individuals with severe dependence, limited availability, the stigma associated with using specialty care, and a host of other barriers means that specialty care alone is unlikely to be able to address the unmet need for treatment.[74,75] Recent federal legislation[76,77] increased coverage for OUD treatment in primary care, and the prevalence of OUD is high among primary care patients.[78,79] Primary care is also an important source of behavioral health care.[80] Community health centers are of particular importance because they are the largest source of primary health care for underserved individuals and 1 in 12 people in the U.S. receives primary care in a community health center.[81]

New Mexico is projected to have <u>the</u> highest death rate from drugs, alcohol and suicide by 2025 and is in the top quintile for age-adjusted opioid overdose death rates.[82] New Mexico ranks 47/50 in rates of suicide death, 50/50 in rates of alcohol-induced death, 45/50 in homicide death and 43/50 in drug overdose death.[82,83] A primarily rural state, it is majority Hispanic.[84] Twenty percent of New Mexico's population live below the poverty level, 9.2% are unemployed and nearly 16% do not have a high school diploma,[84] contributing to high levels of need. Almost every county in New Mexico is designated as a

HPSA, and the current workforce is estimated to meet only 23% of the healthcare need, making interventions to improve access and increase provider effectiveness crucial.[85,86] The overdose death rate increased 80% between 1999 and 2014, with heroin accounting for 34% of the unintentional overdose deaths and prescription opioids for 48%.[87,88] Statewide, the rate of OUD among Medicaid recipients is nearly three times the national rate.[89] In 2018, eight of New Mexico's counties had no buprenorphine prescribers, and fewer than 20% of specialty treatment providers offered buprenorphine maintenance.[90] New Mexico's high rates of OUD, overdose death rates and shortage of health care professionals make it a high-need state where a CC model could have particular relevance, and its mixed rural and urban setting and diverse population give it the potential to demonstrate the impact of effective interventions across a wide range of populations and settings.

We are adding additional sites in Los Angeles because of recruitment challenges. In addition, adding these additional Los Angeles sites expands our sample beyond a single state which will strengthen generalizeability. The new sites will also provide a broader context for understanding variation in implementation.

Increasing access and quality in primary care could improve outcomes. OUD, depressive disorders and PTSD can be treated effectively with FDA-approved medications and/or brief psychotherapies, making them ideal for treatment within primary care.[91-93] However, despite the potentially promising role of primary care in increasing access, only a small percentage of people who need treatment receive it.[73,94,95] And, even when patients receive care, the quality of behavioral health care provided is low, contributing to poor outcomes.[34,46,47,62,96,97]

There is a strong scientific premise for testing the effectiveness of CC in COD patients. Substantial evidence supports the use of CC for behavioral health disorders,[98] and for behavioral health conditions co-morbid with medical illnesses.[99-102] Significantly, CC programs are highly effective for safety net patients and can reduce health disparities in treatment utilization (which we define as access).[103-108] Work by our team demonstrated the effectiveness of CC for patients with opioid and alcohol use disorders,[109-113] depressive disorders,[114-117] depressive disorders co-morbid with substance misuse,[118,119] and PTSD,[120] but CC has never been tested for COD.

In our prior work, we identified patient engagement with individual treatment components as a key challenge.[109,110,120-122] Our prior work indicated two problems with access and quality: many patients were not ready or motivated for treatment, and, when they were, getting timely access to a PCP or BHP appointment was challenging. In our current conceptualization of CC-COD we address patient treatment readiness by having the Care Coordinator use MI to elicit change talk and motivation and make home/community visits. We address problems with timeliness by programming the registry to facilitate coordination between the Care Coordinator and the PCP and BHP. We will advance the science of CC effectiveness by testing whether patient engagement mediates CC outcomes in Aim 3, and by examining effectiveness across diverse settings.

It is unknown what mediates the effect of CC on treatment quality and outcomes.[22,98,120,123-127] Based on our prior work,[109,110,120-122] we propose to test mediators that are strong proxies for engagement:

working alliance[22,123] and patient experiences[21] of care. A central element of CC is the relationship or alliance between the patient and the Care Coordinator, and there is evidence that this relationship is critical to successful outcomes.[22,98,120,123-127] Studies show that the patient-provider working alliance is positively related to improved patient satisfaction, increased compliance with medications, and greater service efficiency.[125] We also hypothesize that positive patient-centered experiences (e.g., good communication with providers, getting treatment quickly, favorable ratings of treatment) will mediate the relationship between the CC-OUD intervention and outcomes, because better patient care experiences are associated with higher levels of adherence to treatment.[128] We will also explore whether the treatment of MH conditions improves OUD outcomes, such as abstinence.

This project has potential for high impact because it both improves public health and advances science. Our study simultaneously addresses critical public health problems—the opioid crisis and the undertreatment of OUD and co-morbid behavioral health problems—and advances science. The undertreatment of OUD is arguably the most important public health problem related to the opioid crisis. In 2015, 11.5 million individuals reported misusing opioids and 1.9 million reported being addicted to opioids,[129] yet fewer than 20% receive any treatment.[130,131] Individuals with COD face similar problems, with fewer than 25% reporting receiving treatment for both conditions. By experimentally testing a new approach—the CC-COD—and assessing implementation factors such as provider acceptability and feasibility, this study could improve public health by identifying an efficient and generalizable model to increase COD treatment delivery and decrease the downstream effects of untreated addiction and mental illness. Our research will advance science by examining whether patient engagement mediates improved outcomes, and whether approaches to improve patient engagement work—important advances in the science of CC.

Background for CR:

Suicide and overdose are prevalent among those with OUD and of heightened concern among those with co-occurring mental illness (COD). Suicidal ideation/behavior[27,132] and overdoses[27,132] are common in individuals with OUD, leading to high rates of morbidity and mortality.[24] About 20-45% of those with OUD also experience either co-occurring depressive disorders[39] and/or post-traumatic stress disorder (PTSD),[38,41,42] and COD contributes to increased risk of death due to suicide (OR = 2.13-4.83)[27] and overdose (OR = 4.34-7.06)[133] compared to OUD alone.[24] MOUD and treatment for mental illness are both associated with reduced mortality, yet access to and retention in treatment is low.[45-48,134,135] Individuals are particularly vulnerable to suicide and overdose immediately after MOUD discontinuation, with comorbidity adding further risk.[48,136-139] Rates of overdose are 2-3 times higher after MOUD discontinuation than among individuals with untreated OUD.[48]

Acronym list	
CC	collaborative care
CC-COD	collaborative care for COD
CC-COD+	Additions to CC-COD to reduce suicide and overdose risk
CLARO	Collaboration Leading to Addiction Treatment and Recovery from Other Stresses
COD	co-occurring disorder
EUC	enhanced usual care
HH	Hubert Humphrey
INSPIRE	Integrating Support Persons Into Recovery
LACDHS	Los Angeles County Department of Health Services
MAP	Map of the Adaptation Process
MDD	major depressive disorder
MI	motivational interviewing
MOUD	medications for opioid use disorder
MV	Mid-Valley Comrehensive Health Center
OUD	opioid use disorder
PCP	primary care provider
Providence	Providence Health & Services – Southern California
PST	Problem Solving Therapy
PTSD	posttraumatic stress disorder
SFC	San Fernando Health Center
SP	support person
UNM	University of New Mexico
WET	Written Exposure Therapy

Reducing mortality in patients with COD is arguably the most important public health problem related to the opioid crisis, and New Mexico is particularly hard hit. New Mexico is in the top quintile for age-adjusted opioid overdose death rates and is projected to have the highest death rate from drugs, alcohol and suicide by 2025.[82] New Mexico's population is also distinct from other hard-hit states because it is majority Hispanic. The overdose death rate increased 80% between 1999 and 2014, with heroin accounting for 34% of the unintentional overdose deaths and prescription opioids for 48%.[87,88] Statewide, the rate of OUD among Medicaid recipients is nearly three times the national rate.[89] Despite the need for MOUD treatment, eight of New Mexico's counties had no buprenorphine prescribers in 2018, and fewer than 20% of OUD providers offered buprenorphine maintenance.[90] Our parent study takes place in 11 rural and urban primary care clinics located in Health Professional Shortage Areas (HPSAs). Understanding whether an enhanced version of CC can reduce suicide and overdose risk in a majority Hispanic population may have implications for the treatment of Hispanics in other states.

Traditional CC models do not do enough to reduce suicide and overdose risk. In traditional models of CC, care coordinators typically measure symptoms and intervene if an individual endorses suicidal ideation. One study, Prevention of Suicide in Primary Care Elderly: Collaborative Trial (PROSPECT), focused on CC to prevent suicide, but prevention was defined as having Care Coordinators follow depression treatment guidelines and informing physicians by letter if a patient was identified as having suicidal ideation, with no proactive component or direct intervention with the patient. PROSPECT showed decreases in suicidal ideation in the CC condition,[140,141] but the effect on suicide attempts and related mortality remains unclear.[140] Mortality was highest among patients with medical comorbidities,[142] which suggests complex patients may need more proactive supports. Our team conducted the first successful trial of CC for OUD in primary care,[91,92,109-112,121,122,143-149] and, like the traditional CC model, suicide risk was only addressed when participants stated they were planning on self-harm. There was no proactive role for Care Coordinators to address suicide or overdose risk. Further, at the time of the study, evidence for naloxone was not widely established. Currently, naloxone is the primary antidote among first responders and in the community to reverse opioid overdose.[150,151] Although New Mexico law mandates that patients being treated with MOUD be given

naloxone, our provider advisory board tells us that overdose risk education is not consistently provided to patients and only rarely to family members.

Reducing suicide and overdose risk in the COD population may require more than what CC-COD offers. In the parent study, we hypothesize that CC-COD will result in increased access to MOUD, depression, and PTSD treatment, and we expect that better treatment will result in better patient outcomes. We understand that treatment retention is likely to be a significant issue, and that poor MOUD retention increases the risk of relapse, overdose and death.[45-48,134,135] The parent study addresses retention by having the CM monitor patient progress via a clinical registry, connect with the patient through routine calls, and refer when additional care is needed. However, the parent study does not include outreach to SPs, despite evidence that SPs can influence whether a patient remains in treatment. CC-COD also does not include proactive content specifically targeting suicide and overdose prevention. Given the high rates of both in this population, more interventions are warranted. In this CR we propose to strengthen CC-COD in three ways to reduce suicide and opioid-overdose risk: (1) intervening with SPs to increase patient treatment retention—particularly MOUD treatment, (2) adding naloxone training and overdose prevention education for both SPs and the patient, and (3) adding the Caring Contacts intervention for patients to decrease social isolation, a risk factor for suicide.

There is a strong scientific premise that involvement of SPs may help strengthen MOUD retention and decrease mortality. The effectiveness of MOUD on overdose and suicide prevention hinges on finding innovative ways to increase retention.[52-61] Family members and other SPs can be an important catalyst for engaging patients in substance use treatment because family members are more likely to recognize warning signs of substance misuse than the affected individual, who may not recognize or admit to symptoms.[56,152-154] Family members tend to be highly motivated and typically want to help their loved one reduce their substance use, improve their relationship, and also alleviate their own difficulties associated with the person's substance use.[155-157] On the other hand, family members can perpetuate the patient's use when they fail to recognize or reinforce the patient's steps toward recovery, act as a barrier to the patient accessing effective treatments, or otherwise unknowingly facilitate the patient's substance use. Family members often have concerns about using pharmacotherapies long term and the safety of opioid agonist therapies. In fact, a common barrier to MOUD retention is the assumption by both patients and families that MOUD is an addictive drug substitute.[158]

Studies of SPs' perspectives are uncommon. *There are few studies of SPs' perspectives on MOUD treatment and naloxone use. When asked, family members report lack of knowledge about administering naloxone and fears of legal consequences that might result from being associated with illegal drug use.*[159-161] Conflict between buprenorphine treatment and obligations to family members is a common reason for treatment drop out.[159-162] Our stakeholders tell us that it would be helpful if SPs knew more about MOUD because they often think buprenorphine is "another form of dope", frequently ask patients when they can stop taking it, and judge them to the point that patients stop telling family members about their buprenorphine use (K. Saffier, personal communication, September 5, 2018). This feedback is consistent with our qualitative work with SPs who are eager for information but feel "there are no classes out there that help loved ones of people on heroin."[163] Thus, engaging family members or other support persons in qualitative research to adapt family interventions

is essential and can ensure messaging is impactful, useful, and acceptable. This may be particularly important in Hispanic communities where family "familismo" and collectivism play a central role in influencing behavior.[164]

The scientific premise for adding naloxone training for patients and SPs as an overdose prevention intervention is strong.[165-168] An increasingly common prevention strategy is to provide laypersons with naloxone and related training,[165,166,169] with most efforts (>80%) targeting persons with OUD.[167,169] Studies show that when family members are trained to use naloxone, their knowledge of overdose risks and the likelihood they will administer naloxone increases,[159,168] and naloxone training of laypersons, including SPs, significantly decreases mortality.[151,170,171] Training increases the odds of administration by 1.35 times, and the likelihood of recovery from overdose is 8.58 times higher when naloxone is administered.[169] The exact impact of SP training on mortality remains unclear, because studies have not isolated the effect of training SPs from other laypersons.

By adding a suicide prevention intervention, CMs may be able to decrease suicide risk. Caring Contacts is a straightforward intervention that seeks to increase social connection (reducing social isolation, a risk factor for suicide) to providers without placing additional demands on distressed patients. The scientific premise is strong, and it is one of the only suicide prevention interventions to show decreased suicide deaths and attempts in randomized trials (up to 2 years post-intervention).[172-175] It is effective in high-acuity populations such as active military and veterans,[176,177] and as an adjunct to ongoing mental health treatment.[178] It has not been tested in primary care, or with individuals with substance use disorders or COD.

The proposed study will have high public health impact. The United States is in the middle of two intertwined epidemics. Suicide and overdose deaths are at record levels. OUD and mental illness are major contributors to both, and individuals with COD are a complex population at high risk. In this population, universal prevention makes good public health sense. Medical and behavioral health providers are overburdened with OUD care and lack the time to proactively address suicide and overdose risk with all patients. In this study, we equip the CM to deliver opioid-overdose and suicide prevention to all COD patients and provide much-needed resources to SPs who are underserved. In doing so, this study could improve public health by increasing MOUD retention and decreasing suicide and overdose risk. Our research will advance science by examining whether educating patients and SPs and using Caring Contacts supported by CC accomplishes these goals. Given that more than 40% of suicide and overdose deaths in 2017 were linked to opioid use, the time is right for tackling these twin epidemics.

RISK/BENEFIT ASSESSMENT

2..1 KNOWN POTENTIAL RISKS

Participation poses the following direct risks to patients:

(1) Disclosure of Sensitive Information: Patients who are referred to the study coordinators, undergo eligibility screening, or participate in the study may be at risk of disclosure of sensitive information on their health including the presence of an addiction or mental health disorder, both stigmatizing conditions. We will protect against unauthorized disclosures of information through a robust data safeguarding plan. Site PIs will be responsible for serving as local data safeguarding officers, assuring adherence to the study data safeguarding plan and training in human subjects protections. Standard procedures will include storing data on secure institutional password-protected servers, using password protection and encryption when transmitting any data (including within a hospital), limiting access to patient identifiers to the smallest number of individuals possible, ensuring that datasets do not have sensitive information in them unless necessary, assigning patients study ID numbers at random to enable removal of other direct Health Insurance Portability and Accountability Act (HIPAA) identifiers from datasets, and ensuring that all individuals who handle study data and study staff are trained in human subjects protections, HIPAA, and study procedures. Individuals who handle data on potential or actual study subjects will be required to avoid any unplanned disclosures of information beyond the study team, and will be required to report any unplanned disclosures.

(2) Psychological Distress: Patients who undergo screening and assessment for OUD, PTSD, or MDD may experience psychological distress. In addition, the questions about suicidality on the Columbia Suicide Severity Rating Scale may identify patients who need immediate attention. We will minimize any potential risk of distress by using a private space to interact with the patients. In completing survey items, we will also use a private space and clearly communicate to all prospective participants that participation is voluntary and explain to participants that they have the option of refusing to answer any of the survey questions, and that they are free to leave the study or end the survey at any time. We will train interviewers to be clinically sensitive to signs of distress and how to respond. At the conclusion of each interview, the interviewer will spend some time debriefing the participant by making 'small talk' and allowing the participant the opportunity to ask questions. If a participant remains distressed at the end of the interview, then the interviewer will be instructed to inform the appropriate member of the study staff and will seek permission to contact their PCP. We will have a protocol in place to address distress and suicidality which will aim to connect patients with a mental health provider either at the clinic or facilitated by the PCP on the research team (Dr. Komaromy) or psychiatrist on the research team (Dr. Watkins) if no mental health support is available at the site immediately upon discovery and within 24 hours.

(3) Adverse Effects of Treatments: Adverse effects of receiving standard-of-care treatment may include adverse drug reactions, side effects, interactions with other medications, medication errors, and other issues. These risks are similar to those of untreated OUD and/or co-occurring PTSD/depression, but the risks of treatment are lower. Decisions about treatment for our study participants will be made as in usual clinical practice: CC-COD consultants will make recommendations to primary treating physicians and patients, who may decline any suggestions to initiate MOUD, or treatment for PTSD or depression.
(4) Financial Costs to Patients: Because patients will be receiving care at a FQHC, all treatments will be available to patients as covered through their insurance (e.g., either covered by Medicaid or on a sliding fee schedule). They will incur no additional financial costs as a result of this intervention.

Participation in the CR will add no additional potential risks to participants.

2..2 KNOWN POTENTIAL BENEFITS

Patients in both trial arms may benefit from the receipt of evidence-based treatment for their substance use and mental health conditions.

2..3 ASSESSMENT OF POTENTIAL RISKS AND BENEFITS

Although there are some risks of temporary psychological distress and untreated disorder, the benefits of having greater access to evidence-based treatments are likely to outweigh the risks.

3 OBJECTIVES AND ENDPOINTS

OBJECTIVES	ENDPOINTS	JUSTIFICATION FOR ENDPOINTS	PUTATIVE MECHANISMS OF ACTION
Primary			
To evaluate the effectiveness of CC-COD compared to EUC.	**Original primary endpoints:**<br>MOUD access:[5] Patients with a new episode of OUD care (i.e., no care for at least 60 days prior) receiving an MOUD prescription within the first 30 days of that care episode<br>MOUD continuity of care:[4] Number of continuous treatment days (i.e., no breaks of more than 7 days) the patient receives MOUD within 180 days of study enrollment<br><br>MDD symptoms: PHQ-9[2] raw score change<br><br>PTSD symptoms: PCL-5[179] raw score change<br>**Revised primary endpoints**:**<br>Buprenorphine access:[5] Number of days until first buprenorphine prescription after study enrollment for patients with a new episode of OUD care (i.e., no care for at least 30 days prior).<br>Buprenorphine continuity of care:[4] Number of cumulative treatment days the patient receives buprenorphine within 180 days of study enrollment<br>MDD symptom severity: PHQ-9[2] at 6 months<br>PTSD symptom severity: PCL-5[179] at 6 months	Patients randomly assigned to CC-COD will have significantly increased access and quality outcomes compared with EUC.	We will test mediators that are strong proxies for engagement: working alliance and patient experiences of care. The relationship or alliance between the patient and the Care Coordinator is a central element of CC, and there is evidence that this relationship is critical to successful outcomes. We also hypothesize that positive patient-centered experiences (e.g., good communication with providers, getting treatment quickly, favorable ratings of treatment) will mediate the relationship between the CC-OUD intervention and outcomes, because better patient care experiences are

OBJECTIVES	ENDPOINTS	JUSTIFICATION FOR ENDPOINTS	PUTATIVE MECHANISMS OF ACTION
			associated with higher levels of adherence to treatment.
Secondary			
To evaluate the effectiveness of CC-COD compared to EUC.	***Mental Health***<br><br>Access to MDD and/or PTSD treatment: Receipt of medication and/or behavioral treatment associated with diagnosis for participants who did not have any visits (behavioral health treatment or medication) for MDD and/or PTSD in 30 days prior to study enrollment.<br><br>Quality of care for MDD:[13] 4 psychotherapy visits in the first 6 months or an adequate (60 day) medication trial for a new episode of MDD care (no MDD care for at least 30 days prior to enrollment)<br><br>Quality of care for PTSD:[13] 4 psychotherapy visits in the first six months or an adequate (60-day) medication trial for a new episode of PTSD care (no PTSD care for at least 30 days prior to enrollment)<br>MDD remission: PHQ-9 < 5 at 6 months, among study participants with probable MDD at baseline (PHQ-8 ≥ 10)<br>MDD response: PHQ-9 score at 6 months less than 50% of baseline score, among study participants with probable MDD at baseline (PHQ-8 ≥ 10)<br>PTSD remission: PCL-5 < 34 at 6 months, among study participants with probable PTSD at baseline (PC-PTSD-5 ≥ 3)<br>PTSD response: PCL-5 score at 6 months less than 50% of baseline score, among study participants with probable PTSD at baseline (PC-PTSD-5 ≥ 3)<br>Active suicidal ideation: Dichotomized Columbia Suicide Severity Rating Scale[11] at 6 months: answer YES to Question 3, 4, and/or 5 and/or YES to Question 7.		

OBJECTIVES	ENDPOINTS	JUSTIFICATION FOR ENDPOINTS	PUTATIVE MECHANISMS OF ACTION
	Substance Use<br><br>Opioid use frequency: Days of opioid use in the past 30 days, measured using SAMHSA National Survey on Drug Use and Health (NSDUH[6]) items<br><br>Opioid overdose events: Opioid overdose events in the previous three months, from Naloxone Overdose Baseline Questionnaire[10]<br><br>**Overall Health**<br>Physical health functioning: Veterans RAND 12-item Health Survey (VR-12)[14] – physical health subscale<br>Mental health functioning: Veterans RAND 12-item Health Survey (VR-12)[14] – mental health subscale		
Tertiary/Exploratory			
To evaluate the effectiveness of CC-COD compared to EUC.	Drug use frequency: Days of use in the past 30 days for five drug categories (prescription opioids, heroin, cocaine/crack, methamphetamine/ other stimulants, and tranquilizers/sedatives), measured using SAMHSA National Survey on Drug Use and Health (NSDUH)[6] items<br>Stimulant use frequency: Days of stimulant use in the past 30 days from NSDUH)[6]<br>Alcohol use: Alcohol Use Disorder Identification Test – Consumption (AUDIT-C)[8]<br>Opioid overdose risk behaviors: Opioid Overdose Risk Assessment[9]<br>Opioid use severity: Patient- Reported Outcomes Measurement Information System (PROMIS) Substance Use Short Form[7]		
To test moderators of access, quality	Buprenorphine access:[5] Number of days until first buprenorphine prescription after study enrollment for patients with a new episode of OUD care (i.e., no care for at least 30 days prior).	Explore what factors moderate the effect of CC-	- Demographics (sex and ethnicity)<br>- Pain levels (PEG Pain Monitor[15])

OBJECTIVES	ENDPOINTS	JUSTIFICATION FOR ENDPOINTS	PUTATIVE MECHANISMS OF ACTION
and<br>outcomes.	Buprenorphine continuity of care:[4] Number of cumulative treatment days the patient receives buprenorphine within 180 days of study enrollment<br>MDD symptoms: PHQ-9[2] at 6 months<br>PTSD symptoms: PCL-5[179] at 6 months	COD	- Housing status (Homelessness Screening Clinical Reminder Tool[16])<br>- Disability and impairment (Sheehan Disability Scale[19])<br>- Trauma/Interpersonal Violence (PTSD checklist for DSM-5, description of worst event[179])<br>- Stimulant use frequency (Days of stimulant use in the past 30 days from NSDUH[6])<br>- Number of care coordinator visits
To test mediators of outcomes.	**Revised primary endpoints:**<br>Buprenorphine access:[5] Number of days until first buprenorphine prescription after study enrollment for patients with a new episode of OUD care (i.e., no care for at least 30 days prior).<br>Buprenorphine continuity of care:[4] Number of cumulative treatment days the patient receives buprenorphine within 180 days of study enrollment<br>MDD symptoms: PHQ-9[2] at 6 months<br>PTSD symptoms: PCL-5[179] at 6 months	Patient experiences will mediate the effect of CC-COD on quality and outcomes compared to participants randomized to EUC.	Clinician communication (AHRQ Consumer Assessment of Healthcare Providers (CAHPS) and Systems[21])<br><br>Ability to access treatment quickly (AHRQ CAHPS[21])<br><br>Satisfaction with treatment (AHRQ CAHPS[21])<br><br>Patient and Care Coordinator working alliance (modified Working Alliance Inventory-General Practitioner[22])

**primary outcomes were revised prior to breaking the study blind. These changes and their justifications are further detailed in section 8.1.

4 STUDY DESIGN

OVERALL DESIGN

Hypotheses. For the intervention effectiveness analysis, we hypothesize that patients randomly assigned to CC-COD/CC-COD+ will have significantly increased MOUD access and quality of care, and depression and PTSD outcomes compared to patients assigned to EUC. To test mediators (patient experiences with care and working alliance with the Care Coordinator) of treatment quality and patient-reported outcomes in exploratory analyses, we hypothesize that patient experiences will mediate the effect of CC-COD/CC-COD+ on quality and outcomes compared to participants randomized to EUC.

Phase. This is a Phase 4 clinical trial to test the effectiveness of collaborative care for a population of patients with OUD and depression and/or PTSD in real world primary care settings.

Design. This study uses a pragmatic, randomized controlled trial (RCT) design implemented in 18 primary care clinics to evaluate whether patients with COD who are randomized to CC-COD/CC-COD+ have improved access, quality and outcomes, as compared with those randomized to EUC. We will adapt our CC model to the New Mexico and California settings and a COD patient population, and use findings on organizational readiness[180] to inform implementation (Aim 1). We will then recruit and randomize 900 individuals with COD to receive either CC-COD/CC-COD+ or EUC, and test its impact on access, quality and patient outcomes (Aim 2). Primary outcomes include initiation of MOUD; quality of care for OUD (MOUD continuity), PTSD and/or depression; mental and physical health and wellbeing; and abstinence. We will also assess whether patient experiences of care and working alliance mediate the impact of CC-COD/CC-COD+ on quality and outcomes, and explore whether mental health treatment at 3 months improves OUD outcomes (Aim 3); exploratory analyses will look at what factors moderate the effect of CC-COD/CC-COD+. Aim 4 assesses contextual factors and implementation outcomes to inform future dissemination, should the model be effective.

Under a classification proposed by Curran et al.,[181] our study is a Type 1 effectiveness-implementation hybrid design because we will simultaneously conduct a multi-site trial to determine the effectiveness of CC-COD/CC-COD+ while also assessing context and implementation. Our trial design is pragmatic,[182] and is designed to improve usual practice and inform clinical and policy decisions. These design characteristics include: collaborations with the health care system to adapt the intervention to local conditions, multiple heterogeneous settings, broad patient eligibility criteria that reflect how the intervention will be used in clinical practice, usual care practitioners, multiple outcomes important to decision-makers, intent-to-treat data analysis, and prospective controls.[182-185] We will randomize at the level of the individual patient. We recognize that with individual-level randomization, contamination may occur and lead to Type II error. We mitigate this concern by powering to detect an effect size smaller than found in prior studies.[186] We note a recent Cochrane review of CC found significant effects, despite the use of individual level randomization in 69/90 (79%) of the studies,[98] and in our previous CC study for addiction treatment with individual level randomization, we found positive effects.[110]

Method. After enrollment and baseline assessment, individual subjects will be randomized 1:1 into CC-COD or EUC. A stratified randomized block design will be used, with the strata determined by site and prior MOUD exposure. A randomization list will be generated for each stratum and include randomly permuted block sizes of 2 and 4. Staff will access the randomization module in the Research Electronic Data Capture system (Redcap). Staff will enter which stratum the patient is in and the intervention arm

assignment will be generated. Enrollment will be continuous with the goal of reaching the desired sample size.

Design considerations for CR:

The parent study is a randomized controlled trial of CC-COD versus EUC with randomization at the level of the individual patient. The CR maintains enrollment of 900 patients into the parent study but adds 4 clinics to the parent trial, allowing us to maintain the same timeline while recruiting 300 additional patients. Within a clinic, recruitment for the parent study (**phase 1**; N=900) will be completed prior to the implementation of CC-COD+ (**phase 2;** N=300), at which point individuals will be randomized to CC-COD+ versus EUC. Thus, the timing of transition from CC-COD to CC-COD+ will vary for each site with each site following the permutated randomized blocks first generated for assigning individuals within their site to CC-COD versus EUC. All data collection procedures for the CR will follow the parent study's protocols. While SPs will be invited to participate in the CC-COD+ clinical intervention, we will not enroll SPs into Aim 2 of the CR because we will not collect any trial data from SPs. We will collect the same baseline and follow-up data on the 300 additional patients who are being enrolled into phase 2.

Study Design and Randomization. We will extend the randomized trial from the parent study to allow for a comparison of CC-COD+ to CC-COD. All participating clinics will start the study with enrolled patients being randomized between CC-COD and EUC (Phase 1). At specific milestones, each clinic will move to randomizing between CC-COD+ and EUC (Phase 2). Milestones will be determined once we start enrollment at the California health care systems and can more accurately assess flow rates. We have begun recruitment into phase 2 of the study for one health care system (FCCH) because they plan to end enrolling patients into the study at the end of 2022. We plan to enroll 900 patients to the parent study using a stratified individual-level randomization, with strata defined by the participating clinics and prior MOUD use. Recruitment will continue until 300 patients are enrolled in the CR using the same stratified individual-level randomization design as the parent study. This plan allows us to complete recruitment of both projects within the parent timeline and minimizes the risk to the parent study since each clinic enrollment and randomization for phase 1 is completed prior to the clinic implementing CC-COD+. Details on dates for clinic transitions to CC-COD+ can be found in the study SAP.

SCIENTIFIC RATIONALE FOR STUDY DESIGN

We carefully considered the comparison condition in conversations with our clinical partners and determined that all patients and providers must have access to the basic elements necessary to provide the continuum of care for COD, including training in the supported treatments. A cluster RCT was not feasible because of the large sample size, and with patient-level randomization it is not ethical to withhold these basic features of care. Additionally, our clinic partners told us that all treatments must be available to all patients at a given clinic. We are aware that in some clinics, Care Coordinator services are already available for some patients creating heterogeneity in the EUC condition. We will document this carefully and consider it if we find differences in outcomes across sites. Care Coordinators assigned to CC-COD will have resources not available to regular Care Coordinators: a patient registry to provide population-based care; training and materials to do measurement-based care; expert consultation with BHCs, case-based learning with other Care Coordinators, and training and supervision in MI and shared decision making.

Rationale for CR study design:

Original Design Choice: We carefully considered our Phase 1-Phase 2 design choice. While it is efficient and maximizes external validity by collecting data from patients at all 16 clinics, we recognize it poses a potential threat to internal validity. During the first 6 months that the CM provides the CC-COD+ intervention, they will continue to have CC-COD patients on their caseload (patients receive CM services for 6 months after enrollment) and it is possible for the CM to deliver the enhanced CC-COD+ intervention to these patients. This “contamination” could impact our ability to determine whether standard CC-COD is effective on its own and could affect the internal validity of the CR since CC-COD+ is being compared to CC-COD (See Figure 4.2 below). However, we believe contamination is unlikely and take steps to minimize the risk. The 3 components supported by CC-COD+ are discrete and independent of CC-COD (Overdose/suicide risk education, Naloxone training and Caring Contacts). The clinical registry will contain a field to help the CM keep the two populations separate, and fields related to SP contact and the suicide prevention intervention would be programmed to exclude CC-COD patients. Similarly, the system used to generate Caring Contacts postcards would be programmed to not include CC-COD patients. We would reinforce the scientific importance of not exposing CC-COD patients to the CC-COD+ intervention during CM training and clinical supervision, doing regular audits, eliciting any potential difficulties and problem solving.

*Revised CR design***:** Because the CR did not enroll sufficient individuals to allow for separate analyses, in consultation with our Research Advisory Board, the NIMH scientific officer and the NIMH project officer, it was decided to combine patients enrolled into the parent study intervention arm, with patients enrolled into the CLARO+ study intervention arm for all analyses. The justification for this was that the objective of the trial was to test the Collaborative Care Model for individuals with co-occurring disorders, and the core model was identical across both the CLARO and CLARO+ trials.

JUSTIFICATION FOR INTERVENTION

We chose CC because it is a multi-faceted service delivery model proven to improve access and quality of care. We also chose to modify traditional CC to maximize its uptake in primary care settings in resource-poor communities and use with patients with multiple co-morbidities (called CC-COD). Our adaptation process is using a collaborative planning and development process to optimize CC-COD to the local setting and for this population, building upon tools and resources we developed for the Substance Use Motivation and Medication Integrated Treatment (SUMMIT) and Violence and Stress Assessment (ViStA) trials. The supported treatments include MOUD, medication treatment for depressive disorders and/or PTSD, motivational interviewing (MI), problem-solving therapy (PST) and written exposure therapy (WET). We chose these psychosocial treatments because they are flexible, easy to implement, applicable to a broad range of clients (including those with subthreshold disorders), and extremely safe. Essential members of the CC-COD team include the Care Coordinator, PCP, BHP (psychotherapist) and BHC. Essential activities of the CC-COD model include population-based care supported by a clinical registry, the use of measurement-based care and treat-to-target practices, shared decision-making and expert consultation by a team of BHCs with complementary expertise.

There are several key differences between CC and CC-COD. We modified CC because of the unique characteristics of the setting (resource-poor, Health Professional Shortage Area, long distances); patients (highly stigmatized, multiple complex conditions, ambivalent about treatment) and organization (community health workers used as care extenders, not nurses).

We have adapted the traditional CC model in several key ways. First, rather than having only a single disorder, patients in this study will have co-occurring disorders making it difficult for a single Care Coordinator to provide behavioral health treatment for all three conditions. To address this difficulty, we will add a psychotherapist to the CC-COD team to address patient complexity and deliver evidence-based therapies. A second challenge is that in these types of settings, having a nurse in the Care Coordinator role is expensive and nurses are scarce. For this study, we will train community health workers to address these challenges. This better leverages scarce professional resources and may also address the prevalent perceptions of stigma in this population. The third challenge is the scarcity of psychiatric expertise and geographic remoteness of several settings which limits the opportunity for psychiatrists to work with the Care Coordinator in person. We will address this challenge by using the ECHO model (Extension for Community Healthcare Outcomes) and multi-disciplinary case conferences that include both pharmacotherapy and psychotherapy experts in addiction and mental illness. (ECHO is remote video-conferencing, for case-based learning, group supervision and mentoring). We will train community health workers to play this role and to address these challenges.

Justification for CC-COD+

Individuals with COD are at higher risk of dying from suicide or overdose than individuals with mental illness or OUD alone.[31-34] The parent study will provide a definitive answer as to whether collaborative care (CC) improves access, quality, and outcomes of care for individuals with COD. While a primary goal is to improve access to and retention in medication treatment for OUD (MOUD) (which is linked to decreased mortality and overdose risk), the interventions CC-COD supports do not proactively address suicide or overdose risk, an important limitation. In addition, CC-COD does not include SPs in the patient’s care. We address these limitations and tackle the public health crisis of increasing deaths from

suicide and overdose with the proposed CR. Because SPs can play an important role in a patient's decision to engage with treatment and in overdose and suicide prevention, we investigate SPs' views and use this information to strengthen CC-COD. This revision will develop and then test the incremental effectiveness of three additional CC-COD components. Care coordinators will (1) educate SPs about MOUD with the goal of increasing patient retention in treatment; (2) train SPs and the patient to administer naloxone and on how to reduce opioid-overdose risk behaviors; and (3) implement Caring Contacts, a suicide prevention intervention that sends compassionate mailed or text messages to individuals to decrease social isolation and reduce suicide risk.

END-OF-STUDY DEFINITION

A participant is considered to have completed the study if he or she has completed the screening and baseline assessment, a 6-month course of the intervention (for up to 13 contacts with the Care Coordinator), and the 3-month and 6-month follow-up assessments.

The end of the study is defined as completion of the 6-month follow-up assessment shown in the Schedule of Activities (SoA).

5 STUDY POPULATION

New Mexico: We conduct our study in New Mexico because it is a state with extremely high need and has diverse populations and settings. New Mexico is projected to have the highest death rate from drugs, alcohol and suicide by 2025 and is in the top quintile for age-adjusted opioid overdose death rates.[82] New Mexico ranks 47/50 in rates of suicide death, 50/50 in rates of alcohol-induced death, 45/50 in homicide death and 43/50 in drug overdose death.[82] Primarily a rural state, it also has urban and suburban counties; nearly every county is a HPSA for either behavioral health or medical care.[86]

California: We identified the new systems in this state through colleagues to boost recruitment given our loss of one of the New Mexico systems. By focusing in Los Angeles County, the RAND team may have local access to the sites which will minimize costs and make the recruitment process more feasible. This is particularly important given the late stage of study entry.

Clinical Settings: We leverage relationships developed by the UNM team, and partner with 18 clinics in five large healthcare organizations that provide care throughout the state of New Mexico and California. We include a map in the Facilities and Resources section. We selected the organizations in New Mexico because we have relationships with them and they provide care in the regions of New Mexico with the highest rates of opioid overdose (northeast and central) or are primarily rural (Southwest),[187] and have varying capacity to deliver treatment, ranging from clinics with 0 to 18 buprenorphine prescribers. We also leverage relationships developed by the RAND team, and will partner with two additional health systems in California for ease of recruitment. Most clinics provide primary care for mostly low-income,

predominantly Hispanic patients, and are in HPSAs for primary care, mental healthcare, or both. Providence Health & Services – Southern California serves a different population of primarily insured and non-Hispanic patients, which adds diversity to the subject pool. Clinic and patient characteristics are shown in Table 5.1 below.

Table 5.1. Characteristics of Collaborating Organizations/Clinics

Clinic Characteristics	First Choice Community Health Care (FCCH)								University of New Mexico (UNM)				Hidalgo Medical Services (HMS)			Providence	LA County DHS		
	Alameda**	Alamosa	Belen	Edgewood	Los Lunas	N. Valley **	S. Broadway	S. Valley	SE Heights	North Valley	SW Mesa	SRMC	CHC	Lordsburg	Med Square	Santa Monica	HHH	Mid Valley	San Fernando Health Center
Health region of NM/CA	central	central	central	NE	central	central	central	central	central	central	central	X	SW	SW	SW	West	South Central	San Fernando Valley	San Fernando Valley
Rural per HRSA/HPSA	no	no	part	part	part	no	no	no	no	no	no	X	yes	yes	yes	no	no	no	no
County	Bernalillo	Bernalillo	Valencia	Santa Fe	Valencia	Bernalillo	Bernalillo	Bernalillo	Bernalillo	Bernalillo	Bernalillo	Sandoval	Hidalgo	Grant	Grant	LA	LA	LA	LA
HPSA primary care	no	no	yes	yes	yes	no	no	no	no	no	no	X	yes	yes	yes	no	yes (score 13)	no	yes
HPSA mental health	yes	yes	yes	no	yes	yes	yes	yes	yes	yes	yes	X	yes	yes	yes	no	yes (score 18)	no	no
FQHC	yes	yes	yes	yes	yes	yes	yes	yes	no	no	no	X	yes	yes	yes	no	no	no	no
# of PCPs (FTEs)	TBD	5	3	5	5	5	6	11	6	3.5	3.25	X	2	4	7	43	17	16	6
# of onsite BHPs*	TBD	2	1	2	1	1	3	4	2	0.7	2	X	3*	1	4*	7	2	3	2
Access to staff psychiatrist	TBD	limited	limited	limited	limited	limited	limited	yes	yes	yes	limited	X	limited	limited	limited	no	no	no	no
# waivered providers*	TBD	5	2	3	0	3	3	7	18	6	5	X	1	2	2	2	12	6	0
# of Care Coordinators*	TBD	2	2	2	2	2	1	4	3	1	2	X	0	0	0	1	1	1	TBD

Screen for OUD	Yes	yes	yes	yes	yes	yes	yes	yes	yes	yes	yes	X	yes	yes	yes	no	no	no	no
Screen for depression	Yes	yes	yes	yes	yes	yes	yes	yes	yes	yes	yes	X	yes	yes	yes	yes	yes	yes	yes
**Patient Demographics**																			
% Hispanic	TBD	87	68	32	75	67	75	89	43	52	67	X	53	53	53	11	16	12	12
% African American	TBD	3	2	<1	<1	3	4	2	5	<1	<1	X	1	1	1	5	25	7	7
% Asian	TBD	<1	<1	<1	<1	2	1	<1	23	<1	<1	X	<1	<1	.<1	8	5	5	5
% Native American/ AK native	TBD	1	2	<1	1	1	1	1	2	<1	<1	X	<1	<1	<1	<1	<1	<1	<1
% Women	TBD	57	57	57	63	55	59	58	63	63	63	X	55	55	55	60	45	50	50
% Uninsured	TBD	15	13	6	13	11	15.5	18	3	5	6	X	8	8	8	1	16	15	15
% Medicaid	TBD	69	>50	40	66	66	63	65	72	29	46	X	28	28	28	1	81	80	80
% Monolingual Spanish	TBD	40	19	4	26	16	33	39	19	8	24	X	3	7	3	2	30	25	25

HRSA=Health Resources and Services; HPSA=Health Professional Shortage Area; NE=north east; SW=south west; AK=Alaskan. *May not be full-time as providers work across sites. limited=available by phone for consultation.**Alameda was added to replace North Valley due to low recruitment. For analytic purposes they are grouped together as one clinic.

INCLUSION CRITERIA

Patient participants will be eligible to participate in the study if they meet the following inclusion criteria:

- Consider this clinic to be their usual source of care
- Age 18 or older
- Probable OUD diagnosis
- Probable PTSD or major depression
- Speak and understand English or Spanish
- Have capacity to give informed consent
- Provide a signed and dated informed consent form.

Pregnant women will not be excluded.
The CR uses the same inclusion criteria

EXCLUSION CRITERIA

Patient participants will not be eligible to participate in the study if they meet the following exclusion criterion:

- The patient requires immediate medical (emergency procedure needed) or psychiatric intervention (i.e., self-injured, active psychosis).
- The patient is receiving both MOUD and psychotropic medication from a provider/s outside of the primary care health system at which the patient is enrolled.
- The CR uses the same exclusion criteria

SCREEN FAILURES

Screen failures are defined as participants who:

1. Do not meet inclusion/exclusion criteria

Screen failures will be given a screening ID. They may be eligible to be rescreened but will be assigned a new screening ID.

STRATEGIES FOR RECRUITMENT AND RETENTION

Recruitment. Across both the parent study and the CR, we will recruit and enroll 1200 adults who are patients in one of 18 clinics from five organizations that provide primary care throughout the states of New Mexico and California. Some patients may be recruited through referral from primary care providers at UNM who are not part of the three clinics participating in the trial. Seven Federally Qualified Health Centers (FQHCs) from First Choice Community Health Care, three large UNM primary care clinics (Southeast Heights, UNM North Valley, and UNM Southwest Mesa) and SRMC, three clinics from Hidalgo Medical Services (also an FQHC), three clinics/sites from the Los Angeles County Department of Health Services, (the Hubert Humphrey Comprehensive Health Center, Mid Valley Comprehensive Health Center, and San Fernando Health Center [which is comprised of Glendale Health Center, West Valley Health Center, and San Fernando Health Center]), and Providence Health & Services – Southern California (Providence Health & Services – Southern California have agreed to partner with the research team on the proposed study. We will recruit patients from these clinics and healthcare systems who screen positive for probable opioid use disorder (OUD) and for co-occurring major depression (MDD) and/or post-traumatic stress disorder (PTSD)).

Because this is a disenfranchised population that may be difficult to recruit, we will use a number of strategies to boost recruitment and retention~~.~~

To identify potential participants, we will use several recruitment strategies across sites. These include anonymous, population-based screening in clinic waiting rooms, referral from clinic staff, direct outreach to potential participants via mailings sent from the clinics to potential participants, plus posters in waiting rooms and exam rooms, and Electronic Health Record Review. We plan to take an adaptive approach in determining the optimal recruitment and retention strategies at each of these diverse clinic sites. We plan a one-month run-in phase at the start of our actual enrollment period in order to evaluate the most effective strategy/ies in each setting and to build these into our ongoing protocol. An additional reason for using a variety of methods is because some of the rural clinics are small, and it would not be cost efficient to station study personnel at them 5 days a week. It is important to note, due to the SARS-CoV-2 pandemic, there have been changes to service and patient flow at clinics, therefore we anticipate recruitment to vary by site. Adaptations will be made on an as needed basis, based on our observations, preliminary recruitment success, and the UNM Office of Research guidance and in consultation with our clinic partners.

Eligibility Screening and Informed Consent. Screening of patients in the clinic waiting rooms will take place via a multi-step process. First, patients will be approached by a trained research assistant (RA) who is employed by the RAND/UNM research team. The RA will inform patients of the existence of the study and ask them if they would be willing to complete an initial <u>anonymous</u> screening to determine eligibility. We conduct eligibility screening anonymously because of research that suggest that patients are more likely to report risky drug use in the context of anonymous screening. Those who agree, will be provided with a tablet on which to complete the eligibility screen. Audio computer assisted self-interview screening (ACASI) will be available to patients with low literacy.

Patients who are eligible (see eligibility criteria in Section C) will be offered enrollment in the study. If they wish to proceed, then they will participate in a detailed informed consent process. The RA (employed by the RAND/UNM research team) will complete a baseline interview in private with the enrolled participant to gather further information, such as overdose history, OUD severity and co-morbid pain. After the baseline interview is completed they will be randomized to either the Collaborative Care (CC-COD) or the Enhanced Usual Care (EUC) arm of the study. Participants who are consented and engage in the interview will be remunerated with $100 for their time and effort. Participants will also be offered a $25 bonus incentive if they either: A) complete the baseline interview the same day as screening eligible, OR B) call the research team back later to complete the baseline interview. For those with only a single probable disorder (either OUD or depression/PTSD) or who are not eligible for the study, we will ask if they would like us to inform their PCP of their screening results, and will inform their PCP if they consent. All incentive payments may be paid in form of cash if allowed by the participant’s clinic. Otherwise, incentives will be paid in the form of a merchandise card. For Providence Health & Services – Southern California, all incentives will be paid with merchandise cards.

Referral for clinic providers. Clinicians are uniquely positioned to aid in recruitment since they know their population and are already aware of potentially eligible patients. In order to increase recruitment, we will train clinic staff about study eligibility criteria and how to refer clients to the study. Clinical providers and staff may refer patients who have a diagnosis of PTSD, depression, or probable OUD (whether or not they are being treated with medications for this condition (buprenorphine or naltrexone)). We allow patients currently in treatment for PTSD or depressive disorders to enroll in the study as long as they continue to report symptoms, because of research that suggests that quality of care for behavioral health disorders is low in primary care settings, although we expect few people to be receiving treatment for either PTSD or depression, based on our conversations with the clinics. These patients will be approached by clinic or research staff, informed of the opportunity to participate in the study by completing an initial anonymous screening to help determine eligibility. They will then be assessed according to the above algorithm (Section 5.1.).

Direct outreach to potential subjects. The study staff (employed by the RAND/UNM research team) will prepare letters of study invitation to participate in the study. These IRB approved materials (stamped, sealed letters) will be addressed and sent out by the clinics to all potential participants and will include contact phone number and email/social media contact information. We will develop posters and post them in the waiting room, along with study contact information. RAs will respond to queries from potential study subjects, and will screen and offer enrollment as described above. This procedure is designed to avoid giving the study staff access to the personal health information for these patients until they elect to participate in the study.

Electronic Health Record (EHR) review. The UNM Clinical and Translational Science Center (CTSC) offers a UNM IRB-approved Patient Recruitment Service (PRS) to identify potential participants for research studies using the UNM EHR. Their procedures include a review of the EHR using ICD codes at 3 month time intervals, then using an “honest broker” approach wherein, they contact these people and ask

them if they are interested in being contacted about participation in a study. If the patient agrees, their contact information will be provided to the Collaboration Leading to Addiction Treatment and Recovery from Other Stresses (CLARO) study staff who will reach out to assess interest, screening and eligibility. We plan to review these procedures with other non-UNM clinics to assess a similar and suitable approach with these clinics. The script that is delivered to the patients regarding the study will be developed by the research staff and approved by the UNM HSC IRB. Please note that the study will not conduct an EHR review for recruitment at Providence.

Recruitment rate and feasibility. We collected preliminary data from each of the original 13 sites (not including the California sites) site to determine potential flow rates. The 2-week pilot identified 1,145 out of 1478 people (77%) who were eligible (over 18 and a patient at the participating clinic) and consented to participate in the pilot. The overall mean age of all participants was 48 years (standard deviation 17.3); 65.2% of participants identified as female, the survey was taken in Spanish by 10.7%, and 3.1% and 1.8% of respondents reported use of prescription pain pills or heroin, respectively in the past 3 months. In total, 51 (4.5%) patients surveyed had probable opioid use disorder (OUD), 214 (18.7%) had probable depression; and 218 (19%) had probable PTSD. Based on responses to screening questions about probable OUD with co-occurring depression and/or PTSD, a total of 27 (2.4%) of participants would be eligible for participation in the CLARO study. Among these 27, 70.4% were female and 11.1% took the survey in Spanish. Prescription pain pill use in the past three months was reported by 66.7% of those eligible, and heroin use (past 3 months) was reported by 44.4%. Of these 27 eligible patients: 46.2% reported not getting any treatment for their substance use or mental health in the past 30 days; 23.1% reported counseling only, 11.5% reported medication only, and 19.2% reported receiving both medication and counseling. Among those receiving counseling or therapy, 54.6% were receiving it at the same clinic or health system we interviewed them at. The majority (85.7%) of those who reported receiving medication for OUD or mental health were receiving that at the same clinic. While clinics reported that about 18.5% of patients were monolingual Spanish speakers, the pilot study only interviewed 142 patients (12.4%) in Spanish. Compared with the number of patients that clinic staff estimated they would identify over a two week period with OUD, depression and PTSD (based on 2018-2019 clinic flow estimates), the pilot study identified a slightly higher number of patients than expected (See Table 5.2). These numbers support the feasibility of meeting recruitment goals for the study. The pilot also suggests that a significant proportion (46.2%) who are eligible are not getting treatment.

These assumptions yield a total of 675 patients enrolled per year and 56 patients enrolled per month. Over 16 months, this will yield 1200 enrolled patients. We recognize that these are estimates, and have allowed for an additional four months of study recruitment in case these estimates are incorrect. Once again, we would like to note, that we could experience a delay in achieving our recruitment goals due to public health directives associated with the effects of SARS-CoV-2. We will make adjustments and modifications to our research and recruitment goals on an as needed basis.

Table 5.2. Summary of pilot results compared to data estimated from EMR in 2018-2019 for two week period.

	All Sites			
	Clinic Flow Estimates (2018-2019)		Pilot, Consented	
	n	%	n	%
**Total patients**	740	---	1,145	---
**Total patients with OUD**	25	3.4%	51	4.5%
**Total patients with Depression**	94	12.7%	214	18.7%
**Total patients with PTSD**	27	3.6%	218	19.0%
**Total patients with OUD + Depression and/or PTSD**	6	0.8%	27	2.4%
**Total Number Monolingual Spanish/Took Survey in Spanish**	137	18.5%	142	12.4%

Study staff will keep logs of all patient enrollments as well as of problems and solutions that arise in recruitment. Staff will also track the number of screening and enrollment refusals, characteristics from the eligibility survey of people who refuse, and reasons for refusal. We will also identify those who drop out of the sample before the last follow-up assessment. These tracking data will be shared with our patient engagement and recruitment board and help us refine recruitment/retention procedures and inform our understanding of potential biases. In addition, we will conduct weekly meetings with the field staff to monitor recruitment and retention progress, troubleshoot and address implementation barriers.

Table 5.3 shows the patient flow rates and based on those data, shows estimated recruitment rates and sample sizes.

Table 5.3 (Part 1)– 10/31/2022 through 2/20/2023

	10/31/2022	11/7/2022	11/14/2022	11/21/2022	11/28/2022	12/5/2022	12/12/2022	12/19/2022	12/26/2022	1/2/2023	1/9/2023	1/16/2023	1/23/2023	1/30/2023	2/6/2023	2/13/2023	2/20/2023
HHH+MidValley Sites																	
Parent	6	6	6	12	12	12	12	12	0	12	12	12	12	12	12	12	12
Supplement	0	0	0	0	0	0	0	0	0	0	0	0	0	0	0	0	0
UNM																	
Parent	2	2	2	2	2	2	2	2	0	2	0	0	0	0	0	0	0
Supplement	0	0	0	0	0	0	0	0	0	0	2	2	2	2	2	2	2
FCCH																	
Parent	0	0	0	0	0	0	0	0	0	0	0	0	0	0	0	0	0
Supplement	3	3	3	3	3	3	3	0	0	0	0	0	0	0	0	0	0
PSJ																	
Parent	1	1	1	1	1	1	1	1	0	1	1	0	0	0	0	0	0
Supplement	0	0	0	0	0	0	0	0	0	0	0	1	1	1	1	1	1
HMS																	
Parent																	
	12	12	12	18	18	18	18	15	0	15	15	15	15	15	15	15	15
TOTAL PARENT																	
TOTAL SUPPLEMENT																	
TOTAL ENROLLED																	

Table 5.3 (Part 2) – 2/27/2023 through 5/22/2023

	2/27/2023	3/6/2023	3/13/2023	3/20/2023	3/27/2023	4/3/2023	4/10/2023	4/17/2023	4/24/2023	5/1/2023	5/8/2023	5/15/2023	5/22/2023	Total New	Total Previous	Combined Total	3 Mo Fu Complete (70%)	6 Mo FU Complete (60%)
HHH+MidValley Sites																		
Parent	12	12	12	12	12	0	0	0	0	0	0	0	0	234	5	239	167.3	143.4
Supplement	0	0	0	0	0	12	12	12	12	12	12	12	12	96	0	96	67.2	57.6
														330	5	335	234.5	201
UNM																		
Parent	0	0	0	0	0	0	0	0	0	0	0	0	0	18	251	269	188.3	161.4
Supplement	2	2	2	2	2	2	2	2	2	2	2	2	2	40	0	40	28	24
														58	251	309	216.3	185.4
FCCH																		
Parent	0	0	0	0	0	0	0	0	0	0	0	0	0	0	329	329	230.3	197.4
Supplement	0	0	0	0	0	0	0	0	0	0	0	0	0	21	25	46	32.2	27.6
														21	354	375	262.5	225
PSJ																		
Parent	0	0	0	0	0	0	0	0	0	0	0	0	0	10	0	10	7	6
Supplement	1	1	1	1	1	1	1	1	1	1	1	1	1	19	0	19	13.3	11.4
														29	0	29	20.3	17.4
HMS																		
Parent														0	35	35	24.5	21
	15	15	15	15	15	15	15	15	15	15	15	15	15	438				
TOTAL PARENT															620	882	617.4	529.2
TOTAL SUPPLEMENT															25	201	140.7	120.6
TOTAL ENROLLED															645	1083	758.1	649.8

In the SUMMIT trial we successfully recruited and enrolled 396 primary care patients with either alcohol or opioid use disorder; in the ViStA trial, we successfully recruited 400 patients from six FQHCs over the course of 18 months. We did not have referrals in either study. In a recent study of which UNM is one of 8 sites (led by Dr. Page; HPC-1503-28122) providing hepatitis C virus (HCV) treatment in patients with active ongoing injection drug use and attending either outpatient community clinics or the UNM Alcohol and Substance Abuse (ASAP) clinic for methadone maintenance therapy, we screened 420 patients and enrolled 82 to be randomized to Patient Navigation or modified Directly Observed Treatment (for HCV treatment). We successfully followed 82% of those randomized. This population (currently injecting) is at significant risk for loss to follow up. With the 11 clinics, three of which are very large, and by including a referral strategy, we are confident we can meet our recruitment targets. Nevertheless, we include a 4 month recruitment cushion if recruitment rates are lower than expected, or which could arise in association with the SARS-CoV-2 pandemic.

Retention. Study subjects will be re-interviewed by telephone 3 and 6 months following enrollment. They will be offered $40 to complete the 3 month interview and $40 to complete the final interview at 6 months, and they will be informed of these incentives at the time of initial enrollment. Incentive payments will be given in the form of a merchandise card. We will contact patients via phone, text or email (as they prefer) to schedule follow up interviews. We will make up to 20 attempts over the course of two weeks to contact the participant for each of the two follow-up interviews, and will follow up with hard copy letters to their address, asking them to contact us. We estimate we may lose up to 20% of enrolled subjects from our outcome assessment, due to lost to follow up, incarceration, and mortality, leaving an analytic N=720. A study being conducted by Dr. Page at UNM treating patients who are actively injecting substances has a cumulative 23% loss at 24 weeks (unpublished data). We discuss how we will minimize loss to follow up below. If all of these methods are unsuccessful, we will ask for help from the clinic staff to contact the patient, and if necessary will contact the patient at the time of one of their scheduled clinic visits to offer them a chance to complete the 3 or 6 month assessment.

We recognize that the hardest subjects to reach in a target population might provide fundamentally different responses than members of the group who are relatively easier to find, introducing non-response bias and threatening the quality of survey statistics and the validity and generalizability of research findings. We will use two categories of methods: strategies to stay in touch over the course of the study, and strategies for locating “lost” respondents. Research suggests that in addition to collecting extensive subject contact information, for substance users, online searching of public records, including jails/prisons and death records, and contacts with friends and family are important strategies when field tracking is not an option.

Strategies to stay in touch with respondents. We will use the following strategies to stay in touch with respondents. (1) Collecting extensive contact and re-contact information at study enrollment, including contact information for two or more family members or friends who may know where the subject is. Contact information includes full name, date of birth, phone, cell, email, work address and phone; and use of social networking sites. (2) Incentives. We will provide a $40 merchandise card incentive for completing the 3-month follow up survey, and $40 merchandise card for the 6-month survey. (3)

Reminder gift item such as a cup or water bottle that contains study contact information with study branding to help respondents remember the study; (4) Social Media. We will document all tracking efforts in a tracking system. DataStat will also maintain a dedicated project telephone line.

Strategies for locating respondents. For subjects who cannot be reached by telephone, email or through third party contacts, we will employ the following methods divided among the groups involved. (1) Look for change of addressing using online free record searches, querying the national address server and the United States Postal Service National Change of Address database (NCOA) (DataStat); (2) Paid online searches using Lexis Nexis and INtelius; directory assistance (SRG); (3) Jail and prison searches using the Federal Bureau of Prisons, Vinelink.com; County Sheriff's websites, State Department of Corrections websites and inmate locators (SRG); (4) Death records and obituaries using the social security death index, and other vital records using state department of vital statistics websites (UNM); (5) Administrative records such as the Department of Motor Vehicles or court records (DataStat).

Strategies that will be used to ensure a diverse, representative sample. First Choice Community Health Center (FCCH) clinics, Southeast Heights Clinic at the University of New Mexico, Hidalgo Medical Services in New Mexico; and Los Angeles County Department of Health Services in California all serve primarily low income, underserved, Hispanic patients. As such, patients enrolled for this study will reflect the ethnic and racial background of patients served in the participating sites. This population as a whole is very diverse. A total of 33% of patients receiving care across sites is non-Hispanic white, with 63% Hispanic, 2% Black, 1-2% Native American and 2% Asian. We recognize that the number of Native American subjects is lower than expected, as 11% of New Mexico's population is Native American. We know that many Native Americans get care through the Indian Health Service, however it is also possible that the clinics are under-identifying Native Americans. We anticipate identifying and enrolling a similar sample for this study. The population will also be diverse in representing both genders with the percent female ranging from 51-63% across sites.

Potential recruitment/enrollment challenges and strategies that can be implemented in the event of enrollment shortfalls (e.g., additional outreach procedures, alternate/back-up referral sources).

Due to the SARS-CoV-2/COVID-19 pandemic, there is a possibility that we may encounter potential recruitment and enrollment challenges. As the recommendations evolve, we will also adapt our research guidelines to be in compliance with directives and guidance shared by our clinic partners. We anticipate that patients may no longer feel as comfortable visiting a clinic for an unnecessary appointment or a study visit. We will remedy this by providing as many IRB approved remote opportunities as possible to continue study participation. If we cannot recruit participants from clinic lobbies or MAT sessions we will broaden our recruitment strategies and heavily target online platforms and advertisements, as well as referral opportunities from the clinics and community partners. We will monitor recruitment and target enrollment numbers closely in order to make timely modifications to our strategies. Additionally, the CLARO research team will work closely with clinic staff and the care management team to monitor barriers to participation in the intervention due to the pandemic (or other reasons) and mediate these challenges for participants. Examples include, but are not limited to provision of transportation assistance, virtual appointments, or flexible scheduling.

Another potential recruitment/enrollment challenge is the stigma associated with substance use disorder and behavioral health diagnoses. We have attempted to address this by providing patients in the waiting room with tablet computers to use for completing screening so that they do not have to have a conversation that is potentially audible to others, and by conducting anonymous eligibility screening. If patients still seem hesitant to complete the screener in the waiting room, we will also arrange a private area for people to complete the computerized screening questionnaires.

Lastly, a possible challenge is identifying an adequate number of potential participants in the target clinics. If recruitment is slower than anticipated, one option would be to expand to other primary care clinic sites at the University of New Mexico. Another would be to work with additional organizations that offer primary care in NM, some of whom have signaled an interest in participating (e.g., Southwest Care organization, and Casa de Salud.) Lastly, we may implement an additional recruitment strategy such as: patient chain referral, also known as snowball sampling or respondent driven sampling. In this recruitment strategy, a participant recruits and refers people he or she knows who may be eligible, and then those new participants recruit people they know, and so on. As the participants share contacts, more participants are added to the study and there is an accumulation of participants over time. This type of recruitment and sampling is a nonrandom sampling technique, similar to convenience sampling, wherein there is not an equal chance for all participants to be chosen. Because snowball sampling allows participants to reach out and find more research subjects, researchers have access to potentially unique, hard-to-reach or marginalized populations. Snowball subject recruitment can be used in both quantitative and qualitative research and relies on the social networks of the participants to gather people for the study. A downside to this technique is the nonrandom nature of participant selection, meaning the results of a snowball sampling study may have limited generalizability to the population at large.

Strategies that will allow for the study team to take advantage of new opportunities (e.g., policy changes or new financing mechanisms that have immediate practice implications). We are aware that UNM or FCCH could potentially begin a contract to provide healthcare in the state prisons and for individuals exiting incarceration. If we anticipated a large influx of recently-incarcerated individuals we could attempt to stratify randomization by recent-incarceration status, and could evaluate for a possible differential impact of the CC intervention on these two strata. The New Mexico Medicaid formulary includes sublingual buprenorphine but not injectable long-acting buprenorphine, and so few patients are receiving this treatment currently. If the injectable form were added to the formulary and the requirement for a prior authorization removed then we could examine whether patients respond differently to the CC intervention if they are treated with the injectable long-acting formulation compared with the sublingual formulation.

6 STUDY INTERVENTION

STUDY INTERVENTION ADMINISTRATION

6..1 STUDY INTERVENTION DESCRIPTION

This is a non-investigational approach utilized with this patient population. The control group of the study will have usual care, the CC-COD group will use collaborative care which is a non-investigational approach already used in this population with a strong evidence basis.

CC-COD intervention. The CC-COD intervention emphasizes the core principles of collaborative care including: patient-centered care (shared decision-making with the patient), population-based care managed through a clinical registry, measurement-based treatment to target (e.g., symptom management questions to assess program and adjust treatment plan as needed), and evidence-based psychotherapy and pharmacotherapy treatments[188] (see Table 6.1 below). The intervention is based on a service delivery approach that uses multi-faceted interventions to improve access and quality of care. It is based on Wagner's Chronic Care Model[189,190] and subsequent modifications.[98,191] We hypothesize that the intervention will improve access to the supported evidence-based treatments including supported treatments include MOUD,[192,193] medication treatment for depressive disorders and/or PTSD, motivational interviewing (MI), problem solving therapy (PST) for MDD, and written exposure therapy (WET) for PTSD.

Table 6.1. Description of how core-principles are integrated in the CC-COD Intervention

**Core-principles**	**CC-COD intervention**
Patient-centered care[194]	• Shared decision-making between the care management team and patients where problems to address are clearly defined, treatment plans are mutually agreed upon (e.g., initiation visit uses a menu of options to do this), and barriers are identified and addressed (e.g., social needs)<br>• Designation of a community health worker as Care Coordinator to engage the patient, actively monitor their progress, make necessary modifications to the treatment plan, and link them to care
Population-based care	• Use of registry to track a caseload of patients<br>• Use of registry flags that inform care coordinators/supervisors/consultants of patients in need of higher-level care (e.g., patients with missed visits, suicidality)<br>• Use of registry to track patient progress by symptoms (e.g., MBCs) and service receipt (e.g., MOUD, WET, PST)
Measurement-based treatment to target	• Routine symptom monitoring through measurement-based care and goal attainment questions to assess progress<br>• Adjusting treatment plans by progress with the goal of symptom remission for all conditions
Evidence-based care	• Offering MOUD, WET, and PST to treat OUD and PTSD/MDD<br>• Using MI to deliver care initiation and monitoring visits<br>• Monitoring initiation, engagement, and retention of MOUD, WET, and PST

Care Coordinators will utilize Motivational Interviewing and meet with patients individually for at least 13 visits over six months. In the first two months, the Care Coordinator will meet weekly with the patient. In month three, they will meet biweekly. In months four through six, they will meet with patients once a month. Visits can be in-person or by phone, and ideally in-person prior to the PCP visit so that the Care Coordinator can relay information to the PCP before their visit (e.g., symptoms, insights regarding barriers to care). Visits can also be conducted more frequently or for longer than six months should the care team decide it is best for the patient. The Care Coordinator first meets the patient in a care initiation visit, where the Care Coordinator builds engagement, assesses various patient domains (e.g., OUD, MDD, PTSD, social needs), discusses the conditions to target, links the patient to care, and coordinates linkages with the PCP and BHP. Assessment includes asking the measurement-based care questions described earlier along with the WellRX to assess social needs, PEG Pain Monitor, and social support measures. The Care Coordinator then collaborates with the patient using MI to assess treatment experiences and barriers to care, provides information about treatment options, and then coordinates next steps with the patient. All but four of the opioid use questions used in measurement-based care, PCL-5, and PHQ-9 are administered monthly. After the care initiation visit, Care Coordinators will then meet with the patient in monitoring visits for the remainder of the six-month intervention period. Care monitoring visits are similar to initiation visits, but abbreviated and also focus on engagement, assessment, and linkages.

The Care Coordinator is supported by a behavioral health consultant (BHC) and a Care Coordinator supervisor with expertise in supervising community health workers. The Care Coordinator meet with BHC and Care Coordinator supervisor, respectively, on a weekly basis to discuss the Care Coordinator's patient caseload. Finally, Care Coordinators enter patient information into a clinical registry on an ongoing basis, which tracks a Care Coordinator's patient caseload and has four main purposes:[195] (1) tracks population-level outcomes and engagement, (2) prompts the Care Coordinator with reminders and alerts to ensure accountable outreach when patients have upcoming appointments or need a higher-level of care, (3) prompts treatment-to-target through ongoing symptom management scores that show trends over time in real-time and flags the Care Coordinator when to consult with the BHC, and (4) facilitates caseload review between the Care Coordinator, BHC, and Care Coordinator supervisor through caseload-level reports that display patient-level ID numbers of those who should be discussed.

Enhanced Usual Care intervention. We carefully considered the comparison condition in conversations with our clinical partners and determined that all patients and providers must have access to the basic elements necessary to provide the continuum of care for COD, including training in the supported treatments. A cluster RCT was not feasible because of the large sample size, and with patient-level randomization, it is not ethical to withhold these basic features of care. Additionally, our clinic partners told us that all treatments must be available to all patients at a given clinic. Thus, EUC includes both the evidenced-based psychotherapy (i.e., Problem Solving Therapy[196,197] for depression and Written Exposure Therapy for PTSD[198-200]) and pharmacotherapy (i.e., MOUD and psychotropic medications for MDD/PTSD) provided in CC-COD, but the primary difference is the absence of a care management team and a clinical registry to coordinate care with the patients in EUC.

CC-COD+ intervention. CC-COD+ adds three components to the existing CC-COD intervention (see Table 6.2). *__Education.__* We draw from materials from our prior work with OUD SPs to provide psychoeducation to reduce suicide and overdose risk.[163,176] In our qualitative work on other related projects, patients and SPs told us they had questions about the process of addiction and buprenorphine (e.g., Is it drug swapping? Is relapse common? How long will they be on buprenorphine?). Often, misinformation led patients to leave treatment because of unsupportive SPs who thought the patient was still using. Thus, our goals for providing education are to explore these common questions about OUD and MOUD and correct any misperceptions. Topics from our other intervention manual includes how addiction is chronic, that buprenorphine is safe and effective, and that MOUD decreases withdrawal symptoms and cravings. CCs will also assess patient's overdose risk using the Overdose Risk Assessment scale and provide personalized feedback on behaviors that escalate risk (e.g., using sedatives within 2 hours before or after using an opioid) and discuss alternate options to lower risk.[9] Finally, CCs will conduct lethal means counseling to decrease suicide risk (e.g., using gun locks or biometric gun safe/boxes, storing ammunition separate from guns; removing guns from the home).[201-203] *__Naloxone training.__* CCs will educate patients and SPs about the importance of naloxone, how to recognize signs of overdose,[204] and will provide patients and SPs with naloxone training.[165,205] *__Caring Contacts.__* We adapt the Caring Contacts intervention to best suit the needs of the target population and setting. Several key aspects of the intervention can be tailored, including the (a) sender, (b) modality of the message, (c) message content, (d) patient population included, and (e) schedule of messages.[206] We anticipate that CCs will mail postcards/send text messages to all CC-COD+ patients with a brief message that does not require a response (see Figure 6.1). Cards will be mailed routinely before the patient's birthday, one week after the first CC visit, and each month thereafter for the six-month intervention period. Each card will be tailored to the purpose (e.g., birthday, after a visit, or "thinking of you"). The patient registry will support the schedule on which CCs should be mailing cards. We will adapt existing protocols used by Dr. Landes for responding to patient replies to Caring Contacts (e.g., messages of thanks, questions, crisis).[176,178] Patients without SPs (~20% based on preliminary data) assigned to CC-COD+ will receive all three components minus the SP education.

Figure 6.1. Example of Caring Contacts message

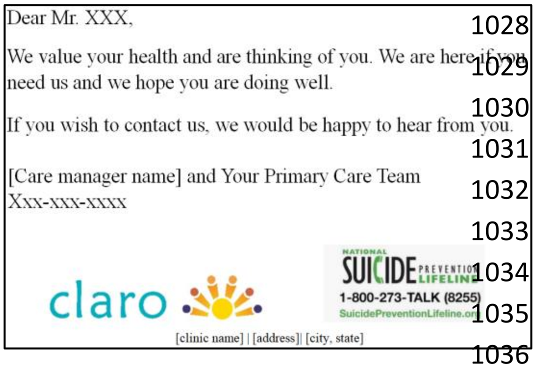

Table 6.2. Description of the new components to the CC-COD+ Intervention

CC-COD+ component	CC-COD+ Activities	Length of time
**(1) Overdose/suicide risk education**	Educate and explore questions about MOUD with SPs<br>Educate SPs about addiction<br>Assess overdose risk and discuss with patient<br>Conduct lethal means counseling with patient	One individual 30-minute session with an SP 18+ nominated by the patient. If the patient wants to nominate more than one adult SP, two can attend this session.<br><br>Overdose risk and lethal means counseling with the patient will be integrated into the patient's care initiation visit with the care coordinator.
**(2) Naloxone training**	Educate patients and SPs on signs of overdose and the importance of naloxone<br>Train patients and SPs on use of naloxone	15 minutes (SPs will receive in their session; patients will receive in their care initiation session)
**(3) Caring Contacts**	Convey concern/compassion via mailed cards<br>Sent to patient weekly/monthly (titrated over six months) and on patient's birthday	Ongoing mailed contacts to patients

6..2 ADMINISTRATION

As stated above, the care coordinators will meet with patients individually for at least 13 visits over six months. In the first two months, the Care Coordinator will meet weekly with the patient. In month three, they will meet biweekly. In months four through six, they will meet with patients once a month. Visits can be in-person or by phone. Care coordinators will be community health workers compared to nurses or behavioral health providers in more traditional collaborative care studies to address the provider shortage in New Mexico and to increase patient buy-in with a peer delivering the intervention. Each patient participant will be assigned one Care Coordinator to interact with during the six-month intervention period. Participants are not expected to interact with other participants in the intervention. Intervention dose will be measured by the number of visits each patient has with the Care Coordinator and these data will be gathered in the clinical registry.

FIDELITY

6..1 INTERVENTIONIST TRAINING AND TRACKING

Care coordinator training: Care Coordinators will receive at least 40 hours of training that correspond to the five core principles of collaborative care. Specifically, Care Coordinators will receive didactics on the symptoms underlying OUD, depression, and PTSD, and what evidence-based approaches will be offered in CC-COD to treat these illnesses. Care Coordinators will also receive MI training and will participate in several interactive exercises and role-plays to practice MI in the context of their rehearsal of CC-COD initiation and monitoring visits. Finally, Care Coordinators will receive training where they will learn to administer the measurement-based care questions, interpret findings to aid treatment planning, track patients in the registry (population-based care), and facilitate communication with the patient and members of the care team. Competency will be assessed while observing practice role-play sessions and

through audio recordings of patient interactions upon starting the clinical trial. Care coordinators will obtain verbal consent to audio record intervention sessions.

Prior to the implementation of the RCT, success of Care Coordinator training will be determined three-fold using role-plays to determine (1) proficiency of motivational interviewing skills, (2) competency in working with the clinical registry, and (3) comprehensiveness of delivering initiation and monitoring visits. As training materials are developed, benchmarks to determine competence in these three domains will be determined. For example, to measure MI proficiency, we may utilize the Motivational Interviewing Treatment Integrity Scale (MITI 4.2.1) and consider a score of 3.5 on the relationship subscale and/or a 3 on the technical subscale as passing criterion.[207] For the registry, we may have several scenarios that Care Coordinators need to accomplish in order to pass (e.g., entering a new patient, resolving a flag, escalating a patient to the BHC, generating a report for the PCP). Finally, for the role-playing a visit, we have a checklist of items to be discussed in each visit and could use a 90% score as a passing metric.

Care coordinator supervision: Care Coordinators receive three hours of weekly supervision. They receive one hour of individual supervision with a behavioral health consultant with an addiction psychiatry background to discuss patients in their caseload. In addition, they will receive group supervision to discuss fidelity to the intervention and other research study concerns. Finally, they will participate in a group Extension for Community Healthcare (ECHO) each week. On alternating weeks, the ECHO will focus on either reflective supervision or case presentation (i.e., a care coordinator presents a case for group discussion). Reflective supervision focuses on reflection between the Care Coordinator and the supervisor to builds on the Care Coordinator’s use of their thoughts, feelings, and values within a service encounter.

CC-COD fidelity: During the RCT, intervention delivery fidelity will be measured through weekly supervision with the BHC and Care Coordinator supervisor who will monitor the Care Coordinator visit attendance including patient visits that were completed or missed in the past week. These meetings will focus on ways to increase delivery fidelity including brainstorming barriers for missed visits and ideas for completing them.

Care coordinator training and supervision for CC-COD+. The training will involve a mix of didactic presentation, demonstration, and role-playing. The training will focus on ways the Care Coordinators can respond to questions about suicide and overdose risk, work with patients who are suicidal or at risk of overdose, coordinate with healthcare providers, and facilitate MOUD retention. In addition, they will learn about harm reduction strategies to reduce overdose risk. Mr. Bernie Lieving, the Statewide Overdose Prevention Education Coordinator for New Mexico’s Office of Substance Abuse Prevention, will provide naloxone training to Care Coordinators. Then the focus will shift to Caring Contacts, including logistics (who, when, how to send messages), ways to respond to patients who reach out, and what resources to use when patients present with risk. Dr. Osilla will provide feedback to each Care Coordinator and practice role-plays with them as needed until the Care Coordinator can cover all intervention content in a MI-consistent manner. As in the parent study, Care Coordinators will meet with a supervisor weekly to discuss patient cases and review intervention content.

CC-COD+ Fidelity: As part of the parent study, we will measure fidelity to the CC-COD intervention during the 3 and 6 month follow-up, with questions asking participants in both the EUC and CC-COD to what extent they interacted with a community health worker that exhibited elements of MI (e.g., collaborative, nonjudgmental), connected them with MOUD and/or behavioral health services and asked them questions about their OUD/PTSD/MDD symptoms. We will add to this measure questions related to whether the participants were exposed to the components of CC-COD+. Specifically, the CC will monitor if the following CC-COD+ activities occurred during a one-month period: (1) SP education session, (2) patient suicide risk session, (3) naloxone session (SP vs. patient vs. both present), and (4) the number of caring contacts cards sent.

MEASURES TO MINIMIZE BIAS: RANDOMIZATION AND BLINDING

Participants will be randomly assigned to a study condition (CC-COD vs. EUC; and then CC-COD+ to EUC) following their baseline assessment, conducted by the research assistant (employed by the RAND/UNM research team). More specifically, this study will use a stratified randomization to assign individuals to CC-COD (or CC-COD+) versus EUC, with strata defined by prior MOUD exposure and site. A randomization list will be generated for each stratum and include randomly permuted block sizes of 2 and 4. We will stratify on prior MOUD to mitigate confounding, as patients who have used MOUD previously are more likely to initiate MOUD subsequently. Research staff will access the randomization module in REDCap. Staff will enter which prior MOUD exposure stratum and site the patient is in, and the intervention arm assignment will be generated. The timing of transition from CC-COD to CC-COD+ will vary for each site with each site following the permutated randomized blocks first generated for assigning individuals with their site to CC-COD versus EUC. Following baseline data collection and randomization, the research assistant (employed by the RAND/UNM research team) will inform the assigned Care Coordinator any time a participant is assigned to the CC-COD or CC-COD+ condition.

Data collection for 3- and 6-month follow-up assessments will be conducted by an independent research firm, DataStat, and the individuals collecting follow-up assessments will also be blind to study condition. DataStat has not and will not be informed of the content of the intervention or the enhanced usual care. Study participants will not be blind to study condition once they have been randomized. To minimize bias, participants will not be told the study hypotheses and be encouraged to provide information that is based on their experiences (e.g., there are no right or wrong answers to the questions). There is minimal risk for potential bias due to the participant telling the DataStat surveyor which treatment group they are in.

In addition to blinding for research procedures, it is worth noting that providers at the primary care clinics will not be blind to participants' assignment to the CC-COD or CC-COD+ study condition because those participants will be assigned a Care Coordinator.

STUDY INTERVENTION ADHERENCE

Intervention participants' adherence with the study procedures will be tracked through the patient registry maintained by the Care Coordinator.

To measure patient fidelity to the CLARO intervention, we will measure whether the following activities occurred in the same month period[208] (see Table 6.2): (1) documentation of a care initiation and monitoring visit in the registry, (2) symptom monitoring defined as documenting scores from the measurement-based care questions in the registry, and (3) documentation of patient progress by printing registry reports for meetings with the behavioral health consultant . Fidelity will be coded in one of three ways: (1) dichotomously (yes/no) if all three activities happened in a one month period (i.e., in prior studies,[208] a "yes" across a two-month period was considered high fidelity; the two-month period could be examined as ever happening during the intervention period or at the early/later stages of the intervention), (2) a count of whether each activity happened each month (e.g., one monthly visit across the six-month intervention period would warrant a score of six, measurement-based care scores entered for only three months in the six-month period would warrant a score of three), and (3) the extent to which each activity happened in the past month (e.g., 0=absent, 1=partially, 2=fully).

Table 6.2. CC-COD Fidelity Definitions

	Within the same month
Care management visits	At least one visit documented in the registry
Symptom monitoring	Scores for OUD and MDD/PTSD symptoms entered once in the registry
Consultation/supervision	One meeting with BHC and one meeting with Care Coordinator supervisor*

*NOTE: BHCs and Care Coordinator supervisors will track attendance. It will be challenging to track whether specific patients were discussed within the month period so we may measure occurrence of consultation/supervision at the case manager-level instead.

Adherence to CC-COD+ will be monitored by the CC supervisor. Specifically, the CC supervisor will monitor if the following activities occurred during a one-month period: (1) SP education session, (2) patient suicide risk session, (3) naloxone session (SP vs. patient vs. both present), and (4) the number of caring contacts cards sent.

7 STUDY INTERVENTION DISCONTINUATION AND PARTICIPANT DISCONTINUATION/WITHDRAWAL

DISCONTINUATION OF STUDY INTERVENTION

A participant will be discontinued from the study intervention when there is clear evidence of harm to patient or staff. The data to be collected at the time of study intervention discontinuation will include the following:

- The reason(s) for discontinuing the participant from the intervention

When a participant discontinues from the study intervention but not from the study, remaining study procedures will be completed as indicated by the study protocol (e.g., the follow-up interviews).

PARTICIPANT DISCONTINUATION/WITHDRAWAL FROM THE STUDY

Patient Requests Study Withdrawal – If a study participant writes to the PI stating that they would like to withdraw from the CLARO study, we will stop collecting survey data and we will not use electronic health record or registry data for evaluation purposes after that date. Patients withdrawing from the CLARO study may continue to receive clinical care and care management for OUD and/or depression/PTSD.

PI Withdraws Patient from Study – A PI may terminate a enrollees participation in the randomized control trial for the following reasons:

- The PI learned that the study participant did not meet inclusion criteria or did meet exclusion criteria at the time of enrollment.
- Other unanticipated reasons

The reason for participant discontinuation or withdrawal from the study will be recorded on the Study Status data collection form. Patients withdrawn from the study by the PI may continue to receive clinical care and care management for OUD and/or depression/PTSD.

LOST TO FOLLOW-UP

Definition of Lost to Follow-Up – Subjects who sign the informed consent form, complete the baseline assessment, and are randomized, but subsequently withdraw, or are discontinued from the study for any reason, will not be replaced. A study participant will be considered to have been lost to follow-for the follow-up wave if it has been more than one month after the target follow-up date. If both the 3-month or 6-month follow-up survey cannot be completed, and follow-up EHR data are unavailable, the patient will receive a lost to follow-up study status.

Retention – Consenting patients will be asked to provide at least two alternative contacts, in addition to their own telephone number, address, and email address, and to agree for us to contact these secondary contacts. In addition, to mailing reminders about completing the follow-up surveys, we will also request permission from patients to text and/or email them reminders to call a toll free number to complete the follow-up phone interview. We will identify ourselves as members of a research project but will not disclose any other details about the purpose for trying to reach the participant. CLARO team members will reach out to clinic staff if contact with the study participant cannot be made at follow-up

in order to retrieve updated contact information. Patients will receive a $40 incentives (in the form a merchandise cards) to complete each telephone follow-up survey.

8 STUDY ASSESSMENTS AND PROCEDURES

ENDPOINT AND OTHER NON-SAFETY ASSESSMENTS

Assessment Procedures. Once a potential participant has indicated interest in the study, trained research staff will screen the individual for eligibility. In the screening assessment, a research assistant (employed by the RAND/UNM research team) will ask the individual's age and whether they are receiving primary care at one of the eleven participating clinics. The research assistant (employed by the RAND/UNM research team) will then administer the NIDA Tobacco, Alcohol, Prescription medication and other Substance use (myTAPS) screener to assess for probable OUD, the 9-item Patient Health Questionnaire (PHQ-9) to assess for probable depression, and the Primary Care PTSD Screen for DSM-5 (PC-PTSD-5) to assess for probable PTSD.

Patients will be eligible to participate in the study if they meet the following criteria: age 18 or older; are receiving primary care at a participating clinic; probable OUD diagnosis, defined by myTAPS[1] scores ≥1 or past 90 day receipt of MOUD; have probable depression (a score ≥10 on the PHQ-9[2]) or probable co-occurring PTSD (having a score ≥3 on the PC-PTSD-5[3]); speak and understand English or Spanish; have capacity to give informed consent; and provide a signed and dated informed consent form. The screening process will be terminated if the patient indicates they do not meet any of the previous criteria. The only other reasons for exclusion from the study are if the patient requires immediate medical (emergency procedure needed) or psychiatric intervention (i.e., self-injured, active psychosis) or if the patient is receiving both MOUD and psychotropic medication from a provider outside of the primary care system at which the patient is enrolled.

Note that we will not administer item 9 from the PHQ-9 ("thoughts that you would be better off dead, or of hurting yourself") during eligibility screening. This avoids asking for information that could require us to break confidentiality before a participant has completed the informed consent process. It does not affect our eligibility criteria because the 8-item and 9-item versions of the PHQ are scored identically.[209] We will obtain a complete initial PHQ-9 score during the baseline interview in one of two ways: (1) if 3 or fewer days pass since the eligibility screener, administer PHQ item 9 during the baseline interview, then combine it with items 1-8 from the eligibility screener for scoring; or (b) if more than 3 days have passed since the eligibility screener, re-administer the full PHQ-9 during the baseline interview and use that full score for all subsequent analyses (this latter option does not affect the eligibility determination).

Patients who screen eligible will be contacted within 4 weeks to participate in the study. The research assistant (RA), who is employed by the RAND/UNM research team, will contact the participant to

schedule a study visit that will consist of a screening questionnaire, the informed consent process and a baseline interview assessment. Due to COVID-19, the consent may or may not be conducted in person. We have created a contingency plan based on recommendations from institutional revisions to research procedures. If the consent process will not be conducted in person, the RA will consent the participant via an IRB approved, virtual method: verbal consent with electronic documentation of the consent process. Regardless of whether or not the consent is obtained via telephone or a video conferencing platform, this should not impact the RA's ability to move directly into the baseline assessment immediately following the consent. The participant will be notified ahead of time that they should plan on spending approximately 1 hour to complete this study visit.

After the subject has consented to participate in the study, they will be given a baseline interview and then randomized to the intervention or control group. Research assistants (employed by the RAND/UNM research team) will administer the baseline assessment in-person, over the phone, or over a video conferencing platform following consent. If the participant is unable to take the baseline assessment immediately following the consent process, the RA will obtain the assessment within two weeks of consenting. If the participant has a time constraint, the RA will stop the assessment after the screening questionnaire and reserve the consent and baseline assessment for another day. After completing the baseline assessment, the participant will be randomized to one of two study conditions: CC-COD (intervention) or EUC (control) (the randomization is described in section 6.3).

DataStat will conduct follow-up interviews by phone 3 and 6 months after study enrollment, with a window of -2 weeks/+4 weeks to conduct each assessment. Follow-up interviewers will be blind to study condition. We will make up to 12 attempts during this window to contact the participant for each of the two follow-up interviews, and will follow up with hard copy letters to their address, asking them to contact us. If all of these methods are unsuccessful, we will ask for help from the clinic staff to contact the patient, and if necessary will contact the patient at the time of one of their scheduled clinic visits to offer them a chance to complete the 3 or 6 month assessment. Research staff will conduct additional follow-up assessments with a review of the electronic health record (EHR).

These procedures are identical for both the parent study and the CR.

Endpoints. Our primary outcomes focus on use of buprenorphine and PTSD and MDD symptom severity. We will also examine a variety of secondary/exploratory outcomes and mediators/moderators of treatment quality and outcomes.

Our original four primary patient outcomes were described as follows: (1) MOUD continuity of care,[4] which refers to the maximum numbers of continuous (i.e., no breaks of more than 7 days) days the patient receives MOUD the 180 days after study enrollment; (2) MOUD access,[5] defined as patients with a new episode of OUD care (i.e., no care for at least 60 days prior) receiving an MOUD prescription within 30 days; (3) MDD symptoms, as measured by the PHQ-9[2]); and (4) PTSD symptoms, as measured by the PCL-5.[179] MOUD continuity and access will be based on prescription (data and frequency) data from the EHR, supplemented by New Mexico Prescription Drug Monitoring Program data if feasible.

During data collection and prior to breaking the study blind, we changed the operationalization of the first two primary outcomes due to data quality issues. Both outcomes were revised to focus on buprenorphine rather than MOUD, due to missing methadone data. Under federal regulations, primary care providers are not permitted to initiate or prescribe methadone for OUD and there is no objective data on methadone initiation from methadone treatment programs equivalent to the data in the PMP. We also do not include injectable naltrexone because in our data fewer than 1% reported receiving it.

We also changed the specification of MOUD continuity of care to be operationalized as cumulative days of care, removing the requirement for no breaks in care of more than seven days. It is important to note that there is no "gold standard" for measuring OUD quality of care.[210-212] While the National Quality Forum measure on which our measure is based has a requirement for no breaks in care, we believe that this requirement introduces measurement bias. We know from clinical experience and the scientific literature, medication nonadherence is common, and that patients will reduce their dose due to cost barriers or in an attempt to wean themselves off, introducing measurement bias.[213,214] Measures of buprenorphine adherence do not include the requirement for no breaks in care[215,216] and patients with percent days covered of >0.80 are classified as adherent. While it is widely agreed upon that longer MOUD care is better, there is no empirical evidence of an ideal length of time, or for how breaks in care are related to long-term outcomes such as mortality.[48,217,218] Recent studies have cautioned against rigidly defining MOUD success and continuity due to patients' nonlinear treatment trajectories and the crucial impact of MOUD even after gaps in care.[211,219,220] Therefore, we believe it is both more meaningful and more valid to assess OUD quality of care as a count of total days of MOUD treatment rather than rely on a contested definition of care discontinuity.

For MOUD access, we changed the specification of the outcome from a binary measure to a time-to-event measure. We specify a time to event measure because 'the number of days until first buprenorphine prescription' is available in the Prescription Monitoring Program (PMP) and allows for more information around the distribution of times until beginning buprenorphine.

We originally proposed to use the EMR to obtain information on patient outcomes but changed the source of data to the participant surveys and PMP. We believe this increases the validity of both primary OUD endpoints the study is measuring. Previous research indicates that the EMR contains missing data at both the variable and observation levels, and that this negatively impacts the validity of quality measures.[221-223] In contrast, all study participants have completed the study screener that contains questions from myTAPS,[1] a clinically validated composite measure to establish probable OUD. In addition, the change in data source for prescription information will result in a more accurate measure for receipt of buprenorphine. In the EMR, providers record prescriptions that have been *written*, and the software does not contain information on whether a patient has ever *filled* the prescription. In contrast, as required by law, the PMP includes information on both the number and duration of prescriptions for controlled substances, including buprenorphine, that have been *filled*. It also includes prescriptions that may have been written by providers outside of the health care system that the patient is assigned to as part of the study. While neither a written prescription nor a filled prescription can be considered a

perfect proxy for medication that has been taken, a prescription fill provides stronger evidence for taking a medication. Where available, we include self-reported data on MOUD use from the participant follow-up surveys.

Our revised outcomes are as follows: (1) Buprenorphine continuity of care,[4] which refers to the numbers of cumulative days the patient receives buprenorphine during the 180 days after study enrollment; (2) Buprenorphine access,[5] defined as number of days until first buprenorphine prescription after study enrollment, among patients with a new episode of OUD care (i.e., no care for at least 30 days prior); (3) MDD symptom severity, as measured by the PHQ-9[2]; and (4) PTSD symptom severity, as measured by the PCL-5.[179] Buprenorphine continuity and access will be based on prescription (data and frequency) data from the follow-up assessments and New Mexico and California Prescription Drug Monitoring Program data. MDD and PTSD symptom severity will be collected in baseline and follow-up assessments, with primary outcome analyses based on final scores across the six-month period.

Given the extensive scope and complexity of the CC-COD/CC-COD+ intervention, we will examine numerous secondary outcomes of interest related to substance use and mental health symptoms, health service utilization and quality, and general patient functioning. Substance use measures include: opioid use frequency, measured as days of opioid use in the past 30 using SAMHSA National Survey on Drug Use and Health (NSDUH[6]) items, and opioid overdose events[10] in the previous three months. Mental health measures include additional analyses of MDD symptom severity at follow-up, based on rates of remission (PHQ-9 < 5) and response (PHQ-9 score less than 50% of baseline score); PTSD symptom severity at follow-up, again including remission (PCL-5 < 34) and response (PCL-5 score less than 50% of baseline score); and active suicidal ideation, measured with the widely used Columbia Suicide Severity Rating Scales[11] and analyzed as a binary measure based on affirmative responses to questions 3, 4, and/or 5 and/or an affirmative response to Question 7.[224] Service utilization and quality measures are all derived from EHR and survey data and include access to MDD and/or PTSD treatment, meaning receipt of medication and/or behavioral treatment associated with a given diagnosis; and for new episodes of MDD or PTSD care (no visits associated with that diagnosis in the previous 30 days), (14) quality of care for MDD,[13] which includes 4 psychotherapy visits in the first six months or an adequate (60 days) medication trial; and (15) quality of care for PTSD,[13] which includes 4 psychotherapy visits in the first six months or an adequate (60 days) medication trial. Finally, we will assess physical and mental health functioning outcomes using the Veterans RAND 12-item Health Survey (VR-12[14]).

A number of patient characteristics could moderate CC-COD/CC-COD+ outcomes. Some characteristics will be measured at baseline only; others will be measured in follow-ups as well, either because they could have a time-varying interaction with the intervention or because the most current information is needed for records retrieval (e.g. National Death Index). Exploratory moderator variables include demographic characteristics (i.e., sex and ethnicity); trauma/interpersonal violence, measured using the PTSD checklist for DSM-5, description of worst event; pain levels, measured for the past week using the PEG Pain Monitor;[15] housing status, measured using the Homelessness Screening Clinical Reminder Tool;[16] disability and impairment, measured using the three-item Sheehan Disability Scale;[19] stimulant use frequency, measured using days of stimulant use in the past 30 days from NSDUH[6] items; and number of care coordinator visits, based on registry entries made by care coordinators.

During follow-up assessments, we will measure key care processes thought to mediate the effects of CC-COD/CC-COD+ on patient outcomes. Specifically, we will examine potential changes in (1) clinician communication, (2) ability to quickly access treatment, and (3) satisfaction with treatment, all using AHRQ Consumer Assessment of Healthcare Providers and Systems survey items,[21] as well as (4) patient and Care Coordinator working alliance using a modified Working Alliance Inventory.[22] Potential mediation will be included in exploratory analyses.

Finally, we have several potential exploratory outcomes: drug use frequency, measured as days of use in the past 30 days for five drug categories (prescription opioids, heroin, cocaine/crack, methamphetamine/ other stimulants, and tranquilizers/sedatives) using SAMHSA National Survey on Drug Use and Health (NSDUH)[6] items; stimulant use frequency, measured as days of stimulant use (cocaine/crack, methamphetamine/ other stimulants) in the past 30 days from NSDUH;[6] alcohol use, measured using the 3-item Alcohol Use Disorder Identification Test – Consumption (AUDIT-C)[8] for the previous 3 months; opioid overdose risk behaviors, measured with the Opioid Overdose Risk Assessment;[9] and opioid use severity, measured using the 7-item Patient-Reported Outcomes Measurement Information System (PROMIS) Substance Use Short Form[7] for the previous 30 days.

SAFETY ASSESSMENTS

Safety data will be gathered from both solicited (baseline and follow-up interviews) and unsolicited reports (e.g. Self-reported by participant or provider) and reported at those specific time intervals as described below in Section 8.3.3.1. We will not report on safety during the screening assessment. All screened participants will be provided a referral as requested and if the information is reportable, the appropriate agencies will be notified.

The safety assessments are identical for the parent study and the CR.

ADVERSE EVENTS AND SERIOUS ADVERSE EVENTS

8..1 DEFINITION OF ADVERSE EVENTS

An AE is any untoward medical or social occurrence in a clinical investigation subject who has received a study intervention and that does not necessarily have to have a causal relationship with the study intervention. An AE can, therefore, be any unfavorable and unintended sign (including an abnormal laboratory finding, for example), symptom, or disease temporally associated with study activities, whether or not considered related to the study intervention.

8..2 DEFINITION OF SERIOUS ADVERSE EVENTS

A Serious Adverse Event (SAE) is an adverse event or suspected adverse reaction that is considered "serious" if, in the view of either the investigator or sponsor, it results in any of the following outcomes:

- Death
- A life-threatening adverse event (i.e., suicide attempt that resulted in admission to hospital)*,
- Inpatient hospitalization or prolongation of existing hospitalization,
- A persistent or significant incapacity or substantial disruption of the ability to conduct normal life functions, or
- A congenital anomaly/birth defect.
- Important medical events that may not result in death, be life-threatening, or require hospitalizations may be considered serious when, based upon appropriate medical judgment, they may jeopardize the subject and may require medical or surgical intervention to prevent one of the outcomes listed in this definition. Examples of such medical events include allergic bronchospasm requiring intensive treatment in an emergency room or at home, blood dyscrasias or convulsions that do not result in subject hospitalization, or the development of drug dependency or drug abuse.

** Life-threatening adverse event. An adverse event is considered "life-threatening" if, in the view of either the investigator or sponsor, its occurrence places the subject or subject at immediate risk of death. It does not include an adverse event, had it occurred in a more severe form, might have caused death.*

The research team will monitor for SAEs from data collected at research visits, from data collected by DataStat, and other potential sources. Any SAEs will be reported according to the IRB requirements of the RAND Human Subjects Protections Committee (HSPC), the University of New Mexico IRB, and the requirements of the NIMH DSMB, as well as each of the partnering clinics that the participant is enrolled through. The initial SAE report will be followed by a submission of a completed SAE report to RAND's HSPC and the NIMH DSMB. In the event that a research subject either withdraws from the study or the investigator decides to discontinue a patient due to an SAE, the patient will be monitored by the investigator via ongoing status assessment until: (1) a resolution is reached, (2) the SAE is determined to be clearly unrelated to the study intervention, or (3) the SAE results in death. Outcomes of SAEs will be periodically reported to RAND HSPC and the NIMH DSMB. A summary of the SAEs that occurred during the previous year will be included in the annual progress report to NIMH. For each SAE, Dr. Miriam Komaromy will serve as the clinical monitor, to determine if the SAE was related to the study. A second opinion will be obtained from an independent monitor if needed.

8..3 CLASSIFICATION OF AN ADVERSE EVENT

8.2.3.1 SEVERITY OF EVENT

Event severity will be graded according to parameters shown below. This process will be started by a research assistant (employed by the RAND/UNM research team), care coordinator or other research staff member and subsequently confirmed or amended by one of the study investigators with clinical

experience. The Project Director and Clinical Investigators (including the study PIs and clinical champions) will be notified immediately if any symptoms are graded severe or higher:

1 = Mild (awareness of a symptom but the symptom is easily tolerated; the symptom requires minimal or no treatment)

2 = Moderate (discomfort enough to cause interference with usual activity)

3 = Severe (incapacitating; unable to perform usual activities; requires absenteeism/bed rest)

4 = Life-threatening

5 = Death

<u>Event Reporting</u>

We will report the following AEs:

01. Self-injury that results in ED Visit
02. Other_____________________

We will report the following SAEs:

01 Suicide attempt resulting in hospitalization
02. Suicide resulting in death
03. Death (not suicide or unsure if suicide)
04. Non-suicidal self-injury that resulted in Hospitalization
05. Hospitalization (not due to suicide attempt or non-suicidal self -injury)
06. Non-lethal Overdose (resulting in ED visit)
07. Other: _____________

8.2.3.2 RELATIONSHIP TO STUDY INTERVENTION

All adverse events (AEs) will have their relationship to the study procedures, including the intervention, assessed by a designated clinical monitor. Dr. Miriam Komaromy will serve as the clinical monitor, to determine if the AE or SAE was related to the study. A second opinion will be obtained from an independent monitor if needed.

The determination of seriousness, severity, and causality will be made by one of the study investigators who is qualified and licensed to diagnose adverse event information, provide a medical evaluation of adverse events, and classify adverse events based upon medical judgement.

Relationship to Study: The clinician's assessment of an AE's relationship to the study is part of the documentation process, but it is not a factor in determining what is or is not reported in the study. All AEs must have their relationship to the study assessed using the terms: related or not related. In a clinical trial, the study intervention must always be suspect. The degree of certainty about the causality will be graded using the following categories below:

Related – There is a reasonable possibility that the study intervention and/or study caused the adverse event. Reasonable possibility means that there is evidence to suggest a causal relationship between the study intervention and/or study and the adverse event.

Not Related – There is not a reasonable possibility that the study intervention and/or study caused the event.

8..3.3 EXPECTEDNESS

We will use a binary classification of the extent to which an adverse event is expected or unexpected. Because the study population includes patients with co-occurring disorders that place them at increased risk for suicidal ideation, asking repeatedly about depressive symptoms, including suicidal ideation, is expected to trigger risk in some cases. Our emergency procedures explain how we will document these events as described in the sections below.

8..4 TIME PERIOD AND FREQUENCY FOR EVENT ASSESSMENT AND FOLLOW-UP

The occurrence of an adverse event (AE) or serious adverse event (SAE) may come to the attention of study personnel during study visits and interviews of a study participant presenting for medical care, or upon review by a study monitor.

As listed in our Incident Report form, all AEs, not otherwise precluded per the protocol, will be captured on that form. Information to be collected includes event description, time of onset, which providers were notified, type of event, and corrective action taken. Assessment of severity and assessment of association will also be included and documented on the Incident Report Form. All AEs occurring while on study will be documented appropriately regardless of relationship. All AEs will be followed to adequate resolution.

Changes in the severity of an AE will be documented to allow an assessment of the duration of the event at each level of severity to be performed. Documentation of onset and duration of each episode will be maintained for AEs characterized as intermittent.

Data collection staff will record events with start dates occurring any time after informed consent is obtained until 7 (for non-serious AEs) or 30 days (for SAEs) after the last day of study participation. At each study visit, the investigator will inquire about the occurrence of AE/SAEs since the last visit. Events will be followed for outcome information until resolution or stabilization.

8.2.5 ADVERSE EVENT REPORTING

For each AE, the interviewer will fill out an Incident Report within 24 hours and email it to their supervisor and the Project Director. If any pertinent information is missing from the email or Incident Report Form, or, it is not clear how a situation was resolved, the supervisor will follow up with the interviewer as appropriate. AEs will be reviewed by the study PIs within 72 hours of being reported. A monthly summary report will be provided to the Principal Investigators, Dr. Katherine Watkins and Dr. Miriam Komaromy, to review all events and ensure reporting and documentation have been conducted in a timely manner. In addition, they will review the log to ensure that each event would NOT have been considered a serious adverse event. They will provide an annual report summarizing the adverse events to UNM's IRB, RAND's Human Subjects Protection Committee (HSPC) and the NIMH Data Safety Monitoring Board (DSMB).

All adverse events and serious adverse events will be tracked in an "Adverse Events" table or form in the study database.

8.2.6 SERIOUS ADVERSE EVENT REPORTING

For SAEs**,** the Incident Report form will be forwarded to the MPIs within 72 hours. If the patient has reported child or elder abuse to clinic staff, the clinic must report the event to the Division of Health Improvement (DHI), Incident Management Bureau (IMB) using the DHI incident report form, available here: *http://dhi.health.state.nm.us/imb/imb_irform.php*. The next step(s) depend(s) on the type of event:

- A PI will report all SAEs to their respective IRBs following IRB protocols for reportable events. For SAEs that are deemed expected and/or unrelated to the study, a PI will include them in the triannual report to the NIMH DSMB. For unexpected SAEs related to the study, a PI will report it to the NIMH Program Officer and DSMB liaison within 10 business days.
- If a patient has died (from any cause), while enrolled in the study, a PI will report it to NIMH PO and DSMB liaison within 5 business days.
- If the patient makes a credible threat to harm another person, Dr. Katherine Watkins and Dr. Valerie Carrejo will consult legal counsel at their respective institutions to determine whether the incident should be reported to authorities within 24 hours.
- If the patient has reported child abuse to the interviewer:
  - For NM sites, the UNM PI will report it, since New Mexico law requires reporting child abuse to local law enforcement or the state's Children, Youth and Families Department (855-333-7233) within 48 hours. If the incident involves "any Indian child residing in Indian country," tribal law enforcement or a social services agency must be notified.
  - For CA sites, the local PI will report it, since California law requires reporting child abuse to local law enforcement or the state's Child Protection Hotline and file mandatory online report (800-540-4000; https://mandreptla.org/) within 36 hours.
- If the patient has reported elder abuse to the interviewer:

  - For NM sites, the UNM PI will report it within 24 hours, since New Mexico law requires reporting elder abuse to Adult Protective Services, 866-654-3219 or 505-476-4912.
  - For CA sites, the local PI will report it as soon as possible or within 2 business days, since California law requires reporting elder abuse to Adult Protective Services, 877-477-3646 or 888-202-4248.
- If the interviewer is injured:
  - For NM sites, the UNM PI will notify UNM Employee Health or other appropriate body, and the interviewer's supervisor will complete any forms required by the organization. If the is harm by a participant, the police will be notified, and that participant will be discontinued from the study.
  - For CA sites, follow local clinic guidelines.

Study staff will complete an incident report form for all Serious Adverse Events. Like AEs, SAEs will be tracked in an "Adverse Events" table or form in the study database. When a SAE is entered into the system, an email will be sent to the PI and relevant research team members for their review. Additionally, a weekly summary report listing the SAEs and their details will be provided to the PI and research study team. If a clinical staff member becomes aware of a SAE they will report it to the care coordinator. Care coordinators are trained on how to document SAEs and report them to the study team.

The following table outlines the NIMH's reportable events policy for AEs, SAEs, death, unanticipated problems, protocol deviations, non-compliance, suspensions and terminations.

**Reportable Event**	**When is Event Reported to the NIMH**	**Reported By**
IRB/ISM/DSMB/OHRP/FDA Suspensions or Terminations	Any suspension or termination of approval must include a statement of the reason(s) for the action and must be reported promptly to the NIMH PO within **3 business days of receipt.**	Regulatory or Monitoring Entity and Investigator
Deaths related to study participation	Deaths must be reported immediately (no later than within **5 business days)** of the principal investigator first learning of the death.	Investigator
Unexpected *Serious Adverse Events* related to study participation	Reported to the NIMH PO within **10 business days** of the study team becoming aware of the SAE.	Investigator
Unanticipated Problems Involving Risks to Subjects or Others	Reported to the NIMH PO within **10 business days** of the investigator learning of the event.	Investigator
Serious or Continuing Noncompliance	Reported to the NIMH PO within **10 business days** of IRB determination.	Institution
Adverse Event	For all AEs and SAEs that are deemed expected and/or unrelated to the study, a summary should be submitted to the NIMH PO with the **annual progress**	Investigator

	**report**.	
Protocol Deviations	With the **annual progress report**.	Investigator

8.2.7 REPORTING OF PREGNANCY

Although not an AE, pregnancies are reportable events. The study team will attempt to obtain data and report on pregnancy and birth outcomes for any pregnancies that occur during the study period in women who have received any study intervention will be reported.

8.3 UNANTICIPATED PROBLEMS

8.3.1 DEFINITION OF UNANTICIPATED PROBLEMS

This protocol uses the definition of Unanticipated Problems as defined by the Office for Human Research Protections (OHRP). OHRP considers unanticipated problems involving risks to participants or others to include, in general, any incident, experience, or outcome that meets **all** of the following criteria:

- Unexpected in terms of nature, severity, or frequency given (a) the research procedures that are described in the protocol-related documents, such as the Institutional Review Board (IRB)-approved research protocol and informed consent document; and (b) the characteristics of the participant population being studied;
- Related or possibly related to participation in the research ("possibly related" means there is a reasonable possibility that the incident, experience, or outcome may have been caused by the procedures involved in the research); and
- Suggests that the research places participants or others at a greater risk of harm (including physical, psychological, economic, or social harm) than was previously known or recognized.

8.3.2 UNANTICIPATED PROBLEMS REPORTING

The investigator will report unanticipated problems (UPs) to the reviewing Institutional Review Board (IRB) and to the UNM principal investigator (PI, Valerie Carrejo). The UP report will include the following information:

- Protocol identifying information: protocol title and number, PI's name, and the IRB project number
- A detailed description of the event, incident, experience, or outcome

- An explanation of the basis for determining that the event, incident, experience, or outcome represents an UP
- A description of any changes to the protocol or other corrective actions that have been taken or are proposed in response to the UP
- To satisfy the requirement for prompt reporting, UPs will be reported using the following timeline:
- UPs that are serious adverse events (SAEs) will be reported to the IRB and to the NIMH Program Officer and DSMB Liaison within 10 business days of the investigator becoming aware of the event.
- Any other UP will be reported to the IRB and to the NIMH Program Officer and DSMB Liaison within 10 business days of the investigator becoming aware of the problem.
- All UPs will be reported to appropriate institutional officials (as required by an institution's written reporting procedures), the supporting agency head (or designee), and the Office for Human Research Protections (OHRP) within 10 business days of the IRB's receipt of the report of the problem from the investigator.

8.3.3 REPORTING UNANTICIPATED PROBLEMS TO PARTICIPANTS

We do not anticipate the need to report unanticipated problems to participants since this study only involves the use of FDA-approved medications and standard clinical treatments. However, in the rare event that there are unanticipated problems (e.g., breaches in privacy or confidentiality), we will report them to participants.

9 STATISTICAL CONSIDERATIONS

9.1 STATISTICAL HYPOTHESES

The Statistical Analysis Plan (SAP) includes all statistical considerations for this study. This protocol provides a summary of these. See the SAP for details.

There are four primary endpoints that will be evaluated. The testable null and alternative hypotheses for the primary outcomes are as follows:

1. We hypothesize that patients with a new episode of OUD care (i.e., no care for at least 30 days prior) who are randomized to CC-COD/CC-COD+ will receive buprenorphine within fewer days after that care episode begins than new OUD patients who are randomized to enhanced usual

care. Alternatively, the null hypothesis is that there will be no difference in the number of days until receipt of buprenorphine with a new OUD episode of care.

2. We hypothesize that patients who are randomized to CC-COD/CC-COD+ will have more days of buprenorphine treatment within 180 days of study enrollment than patients who are randomized to enhanced usual care. Alternatively, the null hypothesis is that there will be no difference in the number of days of cumulative buprenorphine treatment within 180 days of study enrollment.
3. We hypothesize that, among patients with probable depression at study enrollment, those who are randomized to CC-COD/CC-COD+ will have a greater reduction in depression symptom severity 6 months after study enrollment compared to those who are randomized to enhanced usual care. Alternatively, the null hypothesis is that there will be no difference in depression symptom severity 6 months after study enrollment among those with probable depression at enrollment.
4. We hypothesize that, among patients with probable PTSD at study enrollment, those who are randomized to CC-COD/CC-COD+ will have a greater reduction in PTSD symptom severity 6 months after study enrollment compared to those who are randomized to enhanced usual care. Alternatively, the null hypothesis is that there will be no difference in PTSD symptom severity 6 months after study enrollment among those with probable PTSD at enrollment.

- Secondary Endpoint(s): See 9.4.3.

9.2 SAMPLE SIZE DETERMINATION

The four primary hypotheses described in the Section 9.1 were used for the power calculations. All calculations are for 80% power at a Type I error rate of 1.25%, which accounts for the multiple primary outcomes using a Bonferroni correction to control the family-wise error rate at 5% (0.05/4 = 0.0125). All calculations assume 900 total study participants and, if relevant, 20% loss to follow up. Initial assumptions for the power calculations were:

1. At enrollment, 50% of study participants will have a new OUD episode of care, defined as no visits with an OUD diagnosis in the past 60 days.

2. At enrollment, one third of study participants will have probable depression, one third will have probable PTSD, and one third will have both probable depression and PTSD.

3. At enrollment, 25% of patients[110] with a new OUD episode of care who receive enhanced usual care will initiate medication for OUD within the first 30 days of that care episode.

4. Among those who initiate medication for OUD who receive enhanced usual care, the mean number of days of continuous treatment for OUD is 80 with a standard deviation is 65 (personal communication, Asa Wilks).

5. Among those with probable depression at enrollment (PHQ-9 ^{3} 10), assume a mean depression symptoms score (PHQ-9) at 6 months of 12 and standard deviation of 6.

6. Among those with probable PTSD at enrollment (PC-PTSD-5 ^{3} 3), assume a mean PTSD symptoms score (PCL-5) at 6 months of 50 and standard deviation of 11.

Simulation-based power calculations were performed by randomly generating outcomes based on the assumptions described here. A total of 1000 random datasets were generated, and each analyzed using the statistical model described in the SAP. We varied the effect size of CC-COD/CC-COD+ across a range of plausible values to determine the power. We have 80% power to detect:

1. A 15 percentage point increase in initiation of medication for OUD within the first 30 days of that care episode. A previous study of collaborative care for opioid and alcohol use disorders found a 22 percentage point increase over enhanced usual care. [110]

2. 14 additional days of continuous OUD treatment within the first 180 days. A growing body of evidence suggests the mortality is lower during OUD treatment, and that mortality is increased in the first four weeks after treatment cessation.[135]

3. A 2 point reduction in depression symptoms (PHQ-9). This provides power to detect effects below the clinically important difference for individual change of 5 points.[225]

4. A 3.5 point reduction in PTSD symptoms (PCL-5). A previous study of delivering PTSD treatment in primary care setting to active duty military found a reduction in PTSD symptoms of 7 points.[226]

Prior to breaking the study blind but after completion of data collection, the above distributions and power calculations were updated in the SAP to reflect observed data.

Dropout is of minimal concern in this study, as all analyses are intention-to-treat. For the outcomes based on self-reported symptoms, i.e. depression symptoms and PTSD symptoms, we expect a 20% loss to follow up. This loss to follow up has been accounted for in the power calculations.

The sample size provides sufficient power for secondary outcomes. Details are included in the SAP.

Original Sample size considerations for CR:

Figure 4.2 (above in Section 4.2) illustrates the sample sizes in each arm and in each phase of the study, along with a conceptual representation of the revision analyses. A total of 900 individuals will be randomized between CC-COD and EUC (Phase I) and a total of 300 individuals will be randomized between CC-COD+ and EUC (Phase II). Based on data from the INSPIRE study, we assume 80% of patients have SPs who are willing to participate in treatment and that the effect of CC-COD+ for the 20% of patients without SPs willing to participate will be the same as CC-COD. Using the same assumptions for the power calculations as above, we have 80% power to detect an incremental effect of CC-COD+ over CC-COD on the number of days of continuous MOUD care of 14 days. A growing body of evidence

suggests the mortality is lower during OUD treatment, and that mortality is increased in the first four weeks after treatment cessation.[135] Two weeks of additional OUD treatment reflects a clinically significant increase in the duration of treatment given these known mortality patterns after treatment cessation. The sample size for the CR was determined based on this clinically meaningful effect size.

As noted elsewhere, it was later decided to combine the CR and parent study populations for all analyses. This decision was made in consultation with our Research Advisory Board, the NIMH scientific officer and the NIMH project officer due to lower than expected recruitment.

9.3 POPULATIONS FOR ANALYSES

All analyses are intention-to-treat based on randomization. The primary study outcomes have the following analysis populations:

1. Buprenorphine access is limited to randomized participants with a new OUD episode of care at enrollment, defined as no visits with an OUD diagnosis in the past 30 days.
2. Cumulative days of buprenorphine for OUD includes all randomized participants.
3. Depression symptom severity includes all randomized participants with likely depression at enrollment, defined as PHQ-8 [3] ≥ 10.
4. PTSD symptom severity includes all randomized participants with likely PTSD at enrollment, defined as PC-PTSD-5 [3] ≥ 3.

9.4 STATISTICAL ANALYSES

9.4.1 GENERAL APPROACH

This protocol provides a summary of the statistical analyses, and the SAP provides additional details.

- Descriptive statistics will be reported by study arm. Categorical data will be presented as percentages, and continuous data will be summarized as means and standard deviations.
- Buprenorphine access will be compared between arms by fitting a logistic regression model. Specification of the model is available in the SAP.
- Cumulative days of buprenorphine will be compared between arms by fitting a linear regression model. Specification of the model is available in the SAP.
- Depression symptom severity and PTSD symptom severity will be analyzed using non-response weighted linear regression models. Additional model details and specification of covariates are provided in the SAP.

9.4.2 ANALYSIS OF THE PRIMARY ENDPOINT(S)

This protocol provides a summary of the statistical analyses, and the SAP provides additional details.

- Buprenorphine access is a time-to-event outcome and will be compared between arms by fitting a Cox proportional hazard regression model. Specification of the model is available in the SAP.
- Days of cumulative buprenorphine treatment will be compared between arms by fitting a linear regression model. Specification of the model is available in the SAP.
- Depression symptom severity and PTSD symptom severity are ordinal scales and will be analyzed using non-response weighted linear regression models. Additional model details and specification of covariates are provided in the SAP.

The primary analyses will be performed for the intention-to-treat population, which consists of all randomized subjects. Procedures for handling missing data are provided in the SAP.

A union-intersection test will be used to test the overall success of the study that combines the four primary outcomes into a single composite hypothesis. In particular, the null hypothesis is the intersection of the four primary outcome null hypotheses (i.e., all null hypotheses are true), and the alternative is the union of the alternative hypotheses (i.e., at least one alternative hypothesis is true). A Bonferroni or Benjamini-Hochberg correction on the individual hypotheses will be used to control the error rate of this composite test. The four primary hypotheses will be analyzed with a Type I error of 1.25%, which accounts for the multiple primary outcomes using a Bonferroni correction to control the family-wise error rate at 5% (0.05/4 = 0.0125).

9.4.3 ANALYSIS OF THE SECONDARY ENDPOINT(S)

Secondary endpoints will be grouped into domains of conceptually related endpoints, and each will be analyzed adjusting for multiple comparison within domain. Preliminary domains are as follows:

Mental Health

- Access to MDD and/or PTSD treatment
- Quality of care for MDD
- Quality of care for PTSD
- MDD remission
- MDD response
- PTSD remission
- PTSD response
- Active suicidal ideation

Substance Use

- Opioid use frequency
- Opioid overdose events

Overall Health

- Physical health functioning
- Mental health functioning

Secondary endpoints will be analyzed using similar approaches as described for the primary endpoints. Additional model details are provided in the SAP. Other statistical issues, e.g. loss to follow up and missing data, will be accounted for using the same techniques as with the primary outcomes.

9.4.4 BASELINE DESCRIPTIVE STATISTICS

Baseline descriptive statistics will be reported by study arm. Categorical data will be presented as percentages, and continuous data will be summarized as means and standard deviations. Two-sample comparisons of study arms will be reported using appropriate statistical tests (e.g. two sample t-tests and chi-squared tests). The SAP provides additional details and a list of baseline characteristics to be summarized.

Baseline Descriptive Statistics for CR:

The CR will use the same baseline descriptive statistics as the primary study.

9.4.5 SUB-GROUP ANALYSES

Analyses of moderators of the effectiveness of CC-COD on study outcomes will be conducted. The sub-groups of interest for these analyses are documented in Section 3. Similar models to those for the primary and secondary analyses will be used, but the models will include the necessary interaction between the randomization assignment and the sub-group. The SAP provides additional details.

Sub-Group Analyses for CR:

N/A

9.4.6 EXPLORATORY ANALYSES

All planned exploratory analyses will be described in the SAP.

10 SUPPORTING DOCUMENTATION AND OPERATIONAL CONSIDERATIONS

10.1 REGULATORY, ETHICAL, AND STUDY OVERSIGHT CONSIDERATIONS

10.1.1 INFORMED CONSENT PROCESS

10.1.1.1 CONSENT/ASSENT AND OTHER INFORMATIONAL DOCUMENTS PROVIDED TO PARTICIPANTS

The consent form used in this study complies with all required regulatory elements. The consent form describes in detail the study intervention, study procedures and the risks involved. The participant will need to give explicit permission prior to participating in the study. This permission will be documented in the data collection form and a copy of the informed consent form will be given to every participant for their records. The informed consent will be available in English and Spanish. The correct form will be selected based on the participant's preference and status of language proficiency. A copy of both consent forms is included with this protocol.

Because SPs will not be participating in any research activities, they will not be enrolled and consented in the CR trial. As part of the consent process for participation in the CR, participants will be told that if they are randomized to CC-COD+, the care coordinator will ask if they have an SP who might be willing to meet with the care coordinator to learn about ways to support the participants recovery. However having an SP is not an inclusion criterion.

10.1.1.2 CONSENT PROCEDURES AND DOCUMENTATION

Informed consent will be administered by a research assistant (employed by the RAND/UNM research team) after screening. If administering the informed consent in person poses a risk of viral infection for the participant or members of the research team, we will administer the informed consent via an IRB approved electronic or virtual method. There will not be any waivers for obtaining an informed consent. If the patient refuses to complete the informed consent form they will not be enrolled in the study. The informed consent form will be available in English and Spanish. Our research team will be inclusive of both English and Spanish speakers in order to be able to administer the informed consent in both languages. If the client speaks only a language other than English or Spanish they will not qualify to participate in the study. Competency and comprehension of the above mentioned procedures will be determined by the research team. The research team will participate in various trainings during their orientation to learn how to make these assessments. If the participant is deemed to lack competency or an appropriate level of comprehension to conduct the study activities, they will not be enrolled into the study. Prospective participants who need surrogate consent will not be asked to participate in the study due to diminished autonomy. Children and individuals younger than 18 years of age will also be excluded from this study. If consented in person, a copy of the signed and dated consent form will be provided to the patient. If consented remotely, a copy of the signed and dated consent will be either sent by mail, email, or may be picked up in-person.

10..2 STUDY DISCONTINUATION AND CLOSURE

This study may be temporarily suspended or prematurely terminated if there is sufficient reasonable cause. Written notification, documenting the reason for study suspension or termination, will be provided by the suspending or terminating party to study participants, investigator, NIMH, and regulatory authorities. If the study is prematurely terminated or suspended, the Principal Investigators

(PIs) will promptly inform study participants, the Institutional Review Board (IRB), and NIMH Program Officer and DSMB Liaison and will provide the reason(s) for the termination or suspension within three business days upon receipt. Study participants will be contacted, as applicable, and be informed of changes to study visit schedule.

Circumstances that may warrant termination or suspension include, but are not limited to:

- Determination of unexpected, significant, or unacceptable risk to participants
- Demonstration of efficacy that would warrant stopping
- Insufficient compliance to protocol requirements
- Data that are not sufficiently complete and/or evaluable
- Determination that the primary endpoint has been met

The study may resume once concerns about safety, protocol compliance, and data quality are addressed, and satisfy the NIMH DSMB and the RAND, and UNM IRBs.

10.1.3 CONFIDENTIALITY AND PRIVACY

The Patient Informed Consent Form will assure potential participants that we will keep all of their responses and information completely confidential, that we will not share or tell their name or anything that may identify them to anyone outside of the research staff, except for the information we told you we would share with the Care Coordinator. Their name will never appear in any project reports or presentations. Information in these reports or presentations will be grouped together with all of the people in the project so no person can be identified.

Information about their participation in the project and any treatment they receive will be documented in their medical record as part of routine clinical care. This information is covered by confidentiality regulations and state and federal laws.

We will also tell them that their information is also protected by a Certificate of Confidentiality (CoC) from the National Institute of Mental Health, which protects researchers from being forced, even by court order or subpoena, to identify them. We explain that a CoC does not prevent them or a member of their support system from voluntarily releasing information about them or their involvement in this research. Additionally, if at any time during the project, they share that they plan to harm themselves or others or we hear about or suspect child abuse, or we hear about or suspect elder/dependent adult abuse, the Certificate does not protect that information from being released to the proper authorities.

General Data Safeguarding Principals

The study PIs will have overall responsibility for ensuring data confidentiality, privacy, and safeguarding and will be responsible for serving as local data safeguarding officers, assuring adherence to the study data safeguarding and transfer plan (DSTP) and training in human subjects protections. A DSTP will be shared with all members of the study team who will need to handle data. NIMH has provided a Certificate of Confidentiality to further protect study patients. All key study staff have completed Confidentiality Agreements and CITI certificates have been assembled by the PIs, who are tasked with

overseeing compliance with procedures for human subjects' protections as well as data and safety monitoring.

The study team will monitor the safety of participants and the validity and integrity of the data in accordance with the Data Safety and Monitoring Plan (DSMP). The level and frequency of monitoring will be commensurate with the risks, nature, and complexity of the clinical trial. Data safeguarding procedures will adhere to standards established by applicable regulations including those by the NIH, the Department of Health and Human Services, recent HIPAA regulations, and standards set by the RAND Corporation Institutional Review Board. The UNM SDCC/RAND SRG is responsible for data collection and monitoring of data quality and completeness and ensuring the integrity of data provided to RAND for analyses. All of these investigators have extensive experience in handling sensitive information.

Standard procedures will include storing data on secure institutional password-protected servers, using password-protection and encryption when transmitting any data (including within a hospital), limiting access to patient identifiers to the smallest number of individuals possible, ensuring that datasets do not have sensitive information in them unless necessary, assigning patients study ID numbers at random to enable removal of other direct HIPAA identifiers from datasets, and ensuring that all individuals who handle study data and study staff are trained in human subjects protections, HIPAA, and study procedures. Individuals who handle data on potential or actual study subjects will be required to avoid any unplanned disclosures of information beyond the study team, and will be required to report any unplanned disclosures. Appropriate data use agreements will be implemented before receipt of any data from the clinic sites, DataStat, the NIMH NDA, the University of Washington, and state vital records offices or before any data is sent to DataStat, RAND, NIMH NDA, or University of Washington.

To monitor data and safety issues, this team will meet regularly throughout the study project, enabling any concerns to be addressed rapidly. Monthly quality control reports will be sent to the data entry staff that will identify missing, out-of-range, and discrepant data and require corrections or justifications.

In addition to safety monitoring by HSPC, this cooperative agreement is also assigned to an NIMH-constituted DSMB (NIMH DSMB) for monitoring of data integrity and participant safety. The NIMH DSMB will be responsible for ensuring that appropriate monitoring systems are in place, that the quality of the monitoring activity is adequate, and that the IRB, PDs/PIs, and NIMH staff are informed of recommendations emanating from monitoring activities overseen by the NIMH DSMB.

Data Storage

All study data collected before 8/15/22 will be stored in a REDCAP database behind UNM Health Science Center's (HSC) firewall on a secured and encrypted password protected server following UNM HSC data security policies. Any identifiable information (such as contact information and medical record number) will be stored in a REDCap database ("project") in an encrypted format behind a firewall on UNM's secured servers and is accessible only to designated personnel on a case-by-case basis. The UNM data center is staffed with onsite security personnel 24x7x365 and provides multilevel physical and logical

security protection including: monitoring, video surveillance, biometric and access card and man-trap access to datacenter floor, encrypted and password protected servers, and restricted logical access, with a dedicated and encrypted data drive behind a firewall. Access to the data is provided only to authorized users through an encrypted transmission channel with a password-protected application interface. UNM will store all data on their secured servers for up to 10 years following completion of the CLARO study, per UNM policies, and will be destroyed at that time or according to NIH guidelines.

All study data collected after 8/15/22 will be stored in a HIPAA-compliant REDCap Cloud database. All participant contact information will also be stored in a Record Management System (RMS) on SRG's secure network segment. Computers on the secure network segment are isolated from the rest of the RAND network (e.g. no Internet access, e-mail or file sharing between these computers and the unclassified network) minimizing the possibility of infection by malicious software and unintentional exposure of sensitive data. The computers on the segment will also employ standard password protection along with file and folder permissions limiting access to appropriate project staff. All servers on the secure network segment are located in the RAND Data Center and all client computers are located in offices with limited OmniLock access. Within the segment the RMS database containing the sample data uses a separate password to restrict access. All data entry, scanning, and RMS functions will be conducted using computers on SRG's secure network segment.

Data Sharing

Section 10.1.11 describes the study's data publication and sharing policy. Data from this study will comply with the NIH requirement to submit to the NIMH National Data Archive (NDA) using standardized formatting. Any requests for anonymized data from this study can be obtained via the NDA.

Data Transmittal

Contact information of participants will be entered into a study database in a separate form from the main study data and will include the participant study ID numbers but not their medical record number (MRN). Designated study staff will create a login and profile with DataStat for their Data Transfer Web Portal to which this contact data will be uploaded for the 3- and 6-month follow-up surveys they will conduct. Similarly, the study team will download data from DataStat via the same portal on a regular schedule (e.g., monthly). This data will be saved on CLARO's secured servers, as described above.

Electronic Health Record (EHR) data for study-related diagnoses, prescribed medications, and behavioral health treatment will be obtained during the patients' enrollment in the study covering the period 6 months before enrollment to their final study visit. Prior to 8/15/22, the clinic staff will send this data to the UNM Statistics and Data Coordinating Center (SDCC) as requested via UNM's Secure File Transfer Protocol (SFTP) for storage on the UNM secured servers and merged with other study data. After 8/15/22, the clinic staff will send this data to RAND SRG as requested via Kiteworks for storage on the HIPAA-compliant REDCap Cloud or the SRG secure segment.

Participant identifiers will also be entered by the Care Coordinators into the patient registry, including name, date of birth, and MRN. This data will only be accessible by pre-authorized clinic and research personnel (Research Assistants, Care Coordinators, Care Coordinator Supervisors, and Clinic Administrators) and CLARO's Behavioral Health Consultants. At study completion, the clinics will send this data to RAND SRG via Kiteworks for storage on the SRG secured segment and merged with other study data.

Identifying information will be used to obtain data on participant mortality from state vital records offices and state prescription monitoring programs (the New Mexico Prescription Monitoring Program and the Controlled Substance Utilization Review and Evaluation System). PMP data will be sent in batches with identifiers to UNM for requests made prior to 8/15/22 and RAND SRG for requests made on or after 8/15/22. The study team will link the PMP data with the CLARO study data using identifiers and then de-identify the PMP data before providing to the the analytic team. These procedures will be reviewed by the IRB and implemented once approved.

Deidentified study data will be uploaded two times per year to the NIMH National Data Archive. A universal subject ID (Global Unique Identifier, "GUID") will be generated for each participant and will be linked to the participants' unique study ID assigned in REDCap. This key will be stored on CLARO secured servers and will not be accessible to anyone outside of the UNM SDCC/RAND SRG staff. All raw data will be uploaded every six months to the NIMH NDA per their policies and in accordance with UNM's information security policies. Additionally, all analysis data sets used for manuscripts must be uploaded to the NIMH NDA, identified by GUID.

The SDCC/RAND SRG will provide deidentified analysis data sets for the final analyses. Participant-level data will be enumerated with their study IDs and no MRNs or other personal identifying information will be shared with them. Data will be transmitted via RAND's secured data transfer protocol, Kiteworks.

10.1.4 FUTURE USE OF STORED SPECIMENS AND DATA

UNM will store all data on their secured servers for up to seven years following completion of the CLARO study, per UNM and federal policies, and will be destroyed at that time or according to NIH guidelines.

RAND will store all data on their secured servers for up to 7 years following completion of the CLARO study, per RAND and federal policies, and will be destroyed at that time or according to NIH guidelines.

10.1.5 KEY ROLES AND STUDY GOVERNANCE

**Principal Investigator**	**Principal Investigator**	**Medical Monitor**
Katherine E. Watkins, MD, MSHS, Senior Natural Scientist	*Miriam Komaromy, MD*	*Miriam Komaromy, MD*

RAND Corporation	*Grayken Center for Addiction, Boston Medical Center*	*Grayken Center for Addiction, Boston Medical Center*
1776 Main Street, Santa Monica, CA 90401	*801 Massachusetts Ave, 1st floor, #1039, Boston MA 02118*	*801 Massachusetts Ave, 1st floor, #1039, Boston MA 02118*
310-393-0411 x6509	*505-715-0394*	*505-715-0394*
kwatkins@rand.org	*Miriam.Komaromy@bmc.org*	*Miriam.Komaromy@bmc.org*

The study includes an independent Research Advisory Board (RAB) comprised of experts in addiction medicine and mental health. The committee meets on an ad hoc basis to advise on the research protocol, study progress and results. CLARO is running the meetings via teleconference by providing structured updates on the project and inviting discussion. The study team will also reach out to selected RAB members on an ad hoc basis to get advice on specific topics. Dr. Komaromy chairs the RAB. Board members will be blind to the randomization of patients to study conditions. This board complements (and does not conflict) with the NIMH DSMB because the focus is on the research content rather than on the monitoring of patient safety.

Dr. Watkins is responsible for the oversight and coordination of the project management for the trial working closely with Drs. Komaromy, Meredith, Carrejo, and Osilla. She will also have responsibilities in guiding the analyses.

Dr. Komaromy has oversight and coordination of the project for local intervention adaptation and implementation working closely with Drs. Watkins, Osilla, and Dopp. She will play a crucial role as liaison with the sites in New Mexico for maximizing the fit of the intervention to the local community. Dr. Komaromy will also work closely with the data collection team to ensure that recruitment goals are met on time and data are of high quality.

Dr. Carrejo is the lead for the UNM team.

Ms. Becker is the Director of the RAND Survey Research Group (SRG) who is working closely with the UNM SDCC team to collect the study data. Ms. Becker will lead all RAND SRG data collection.

Dr. Beth Ann Griffin is the RAND statistician leading the RCT design, sampling plan, power calculations, and analysis of the RCT data.

Dr. Dopp is leading the intervention implementation team and measurement development.

Dr. Evans is leading the organizational readiness team working closely with Drs. Ober and Meredith as well as Mr. Bharadwaj.

Dr. Meredith is contributing expertise about collaborative care and mental health throughout the project including planning and implementation of the RCT design, intervention adaptation, and organizational readiness teams.

Dr. Murray-Krezan, at the University of Pittsburgh (formerly at UNM), is the biostatistician responsible for oversight of all data management activities including reporting and analysis of recruitment, screening, enrollment, and safety data for data collected prior to 8/15/22.

Dr. Ober is providing her expertise from SUMMIT on organizational readiness.

Dr. Osilla is leading the intervention adaptation team working closely with Drs. Watkins, Komaromy, Dopp, and Meredith at RAND.

10.1.6 SAFETY OVERSIGHT

Safety oversight will be under the direction of a Data and Safety Monitoring Board (DSMB) convened by NIMH. The DSMB includes experts in scientific disciplines needed to monitor the data and ensure patient safety during the conduct of this study, including addiction and mental health experts, clinical trial experts, biostatisticians, and bioethicists. DSMB members have no association with the project investigators, and no conflicts of interest with study outcomes. The DSMB will meet at least three times a year to assess safety and data of the study. The DMSB will operate under the rules of an approved charter. The DSMB will provide its input to NIMH staff.

DSMB procedures conform with usual standards, including reviewing emerging trial data and maintaining confidentiality. The main responsibilities of the NIMH DSMB include, but are not limited to the following: (1) reviewing the research protocol, consent form(s) and plans for data and safety monitoring prior to the initiation of the study; (2) monitoring of the progress of the study, including data quality, timeliness, recruitment and retention of study participants, adverse events, serious adverse events (SAEs), reasons for participant withdrawal, adherence to the timeline of the study, protocol deviations, performance across study sites, and factors that may affect the risks and benefits of the study such as emerging literature; and (3) making directives about the continuation, modification, or termination of the study, based on the balance of adverse events and beneficial outcomes. Throughout the study, notification of SAEs as well as any proposed investigator-initiated changes in the protocol will be submitted to the NIMH DSMB. Based on its review of the protocol, the NIMH DSMB will identify the data parameters and format of the information to be regularly reported. The NIMH DSMB may at any time request additional information from the Principal Investigators.

All SAEs and adverse events (AEs will only be reported to the NIMH DSMB annually) will be tabulated and submitted to the NIMH DSMB in the triannual DSMB data reports. Based on review of safety data, the NIMH DSMB will issue directives concerning the conduct of the study. Recommendation/directives made by the DSMB may include amending safety monitoring procedures, modifying the protocol or consent, terminating the study, or continuing the study as designed.

10.1.7 CLINICAL MONITORING

10.1.8 CLINICAL MONITORING WILL BE PERFORMED BY THE INDEPENDENT NIMH DATA SAFETY AND MONITORING BOARD (DSMB) AND THE RAND SITE. QUALITY ASSURANCE AND QUALITY CONTROL

Data Collection Training. All research assistants (RAs) will receive comprehensive in-person or online training for accessing and using REDCap for data collection. The UNM Statistics and Data Coordinating Center (SDCC) will assist the RAs in obtaining individual and secured access to REDCap for data collected prior to 8/15/22. RAND SRG will assist the RAs in obtaining individual and secured access to REDCap for data collected on or after 8/15/22. SDCC and SRG will ensure RAs receive help troubleshooting any problems that may arise during the course of the study. Access to the CLARO REDCap database will be limited to data entry only; users will not be able to download any data. Should there be any staff turnover, the departed staff's access to REDCap will be removed. The SDCC manages all access and permissions for UNM REDCap access; SRG manages all access and permissions for REDCap Cloud access.

Quality control of data collected. All study data will be entered in real time to the REDCap data capture system. Range and required field checks will be automatically implemented upon data entry. The SDCC will create monthly standardized quality control (QC) reports with queries for the RAs to review and correct and/or confirm. SRG will continue to implement all QC procedures.

Source documents and the electronic data. Data will be captured electronically in the REDCap database and only in the event of internet instability or power failure will data be captured on paper source documents and entered into REDCap as soon as possible. See **Section 10.1.9, Data Handling and Record Keeping**. If a paper data collection form (source document) is used, then to ensure accuracy research staff will compare the source data against the database. Paper documents will be stored in a locked file cabinet and will be destroyed seven years following study completion.

Protocol Deviations. Protocol deviations will be entered into the "Protocol Deviations" data capture form. The study team will review a weekly protocol deviations report and will implement corrective actions when the quantity or nature of deviations are deemed to be at a level of concern.

10.1.9 DATA HANDLING AND RECORD KEEPING

10.1.9.1 DATA COLLECTION AND MANAGEMENT RESPONSIBILITIES

Data collection will be the responsibility of the clinical trial staff (RA) under the supervision of the Clinical Research Manager and SRG Survey Coordinators. The investigator will be responsible for ensuring the accuracy, completeness, legibility, and timeliness of the data reported.

Any source documents will be completed in a neat, legible manner to ensure accurate interpretation of data.

Hardcopies of the study visit instruments will be provided for use as source document for recording data for participants consented/enrolled in the study, only in the event of loss of internet or power such that REDCap cannot be used. Data recorded in the electronic case report form (eCRF) derived from source documents will checked for consistency with the data recorded on the source documents.

All administered instruments and adverse events (AEs) prior to 8/15/22, will be entered into the UNM REDCap system, a 21 CFR Part 11-compliant data capture system provided by the University of New

Mexico Data Coordinating Center. The data system includes password protection and internal quality checks, such as automatic range checks, to identify data that appear inconsistent, incomplete, or inaccurate. After 8/15/22 all administered instruments will be entered into the HIPAA-compliant RAND REDCap Cloud. See section 10.1.3 for additional information on data storage.

10.1.9.2 STUDY RECORDS RETENTION

Study documents including data stored electronically will be retained for a minimum of seven years following completion of the study. No records will be destroyed without the written consent of the NIMH, if applicable. It is the responsibility of the sponsor/funding agency to inform the investigator when these documents no longer need to be retained.

10.1.10 PROTOCOL DEVIATIONS

The term “protocol deviation” is not defined by either DHHS human subjects regulations (45 CFR 46) or FDA human subjects regulations (*21 CFR 50*). A protocol deviation is a departure from the approved protocol’s procedures made with or without prior IRB approval. Such departures may be major or minor/administrative in nature and result from different sources.

Examples of protocol deviations include:

- Inadequate or delinquent informed consent.
- Inclusion/exclusion criteria not met.
- Unreported serious adverse events.

It will be the responsibility of the Co-PIs (Drs. Watkins, Komaromy, & Carrejo) to use continuous vigilance to identify and report deviations within 10 working days of identification of the protocol deviation, or within 10 working days of the scheduled protocol-required activity. All deviations will be addressed in study source documents and reported to the PIs. Protocol deviations will be sent to the reviewing Institutional Review Board (IRB) and the NIMH per their policies. The site investigator will be responsible for knowing and adhering to the reviewing IRB requirements. Further details about the handling of protocol deviations will be included in the MOP. Protocol deviations will be entered into CLARO database in the “Protocol Deviations” form and a regularly scheduled report of all protocol deviations will be sent to the PIs.

For California sites all protocol deviations will be reported to the PIs.

10.1.11 PUBLICATION AND DATA SHARING POLICY

National Institutes of Health (NIH) Public Access Policy, which ensures that the public has access to the published results of NIH funded research. It requires scientists to submit final peer-reviewed journal manuscripts that arise from NIH funds to the digital archive PubMed Central upon acceptance for publication.

This study will comply with the NIH Data Sharing Policy and Policy on the Dissemination of NIH-Funded Clinical Trial Information and the Clinical Trials Registration and Results Information Submission rule. As such, this trial will be registered at ClinicalTrials.gov, and results information from this trial will be submitted to ClinicalTrials.gov. In addition, every attempt will be made to publish results in peer-reviewed journals. Data from this study will comply with the NIH requirement to submit to the NIMH National Data Archive (NDA) using standardized formatting. Any requests for anonymized data from this study can be obtained via the NDA.

10.1.12 CONFLICT OF INTEREST POLICY

The independence of this study from any actual or perceived influence is critical. Therefore, any actual conflict of interest of persons who have a role in the design, conduct, analysis, publication, or any aspect of this trial will be disclosed and managed. Furthermore, persons who have a perceived conflict of interest will be required to have such conflicts managed in a way that is appropriate to their participation in the design and conduct of this trial. The study leadership (MPIs Katherine Watkins and Miriam Komaromy) in conjunction with the National Institute of Mental Health has established policies and procedures for all study group members to disclose all conflicts of interest and will establish a mechanism for the management of all reported dualities of interest.

10.2 ABBREVIATIONS AND SPECIAL TERMS

AE	adverse event
AHRQ CAHPS	Agency for Healthcare Research and Quality, Consumer Assessment of Healthcare Providers and Systems
AUDIT-C	3-item Alcohol Use Disorder Identification Test – Consumption
BHC	behavioral health consultant
BHP	behavioral health psychotherapist
CC	collaborative care
CC-COD	collaborative care for COD
CFR	Code of Federal Regulations
CA	California
CMP	clinical monitoring plan
CLARO	Collaboration Leading to Addiction Treatment and Recovery from Other Stresses
COD	co-occurring disorder
CRF	case report form
DSMB	Data Safety Monitoring Board

DSMP	Data Safety Monitoring Plan
EHR	electronic health record
EUC	enhanced usual care
FCCH	First Choice Community Healthcare
FDA	Food and Drug Administration
FQHC	Federally qualified health centers
GCP	good clinical practice
HCA	Hepatitis C virus
HH	Hubert Humphrey
HIPAA	Health Insurance Portability and Accountability Act
HMS	Hidalgo Medical Services
HPSA	health professional shortage area
ICH	International Council on Harmonization
LACDHS	Los Angeles County Department of Health Services
IRB	Institutional Review Board
MDD	major depressive disorder
MH	mental health
MI	Motivational interviewing
MV	Mid Valley Comprehensive Health Center
MOP	Manual of Procedures
MOUD	medications for opioid use disorder
NCT	National Clinical Trial
NIH	National Institutes of Health
NIMH	National Institute of Mental Health
NM	New Mexico
NSDUH	National Survey on Drug Use and Health
OHRP	Office for Human Research Protections
OUD	opioid use disorder
OV-ERI	Olive View Education and Research Institute
PC-PTSD-5	Primary Care PTSD 5-item checklist
PCL-5	Posttraumatic stress disorder checklist for DSM-5
PCP	primary care provider
PHQ-9	Patient Health Questionnaire for Depression – 9 item
PI	Principal Investigator
PROMIS	Patient-Reported Outcomes Measurement Information System
Providence	Providence Health & Services – Southern California
PST	problem solving therapy
PTSD	posttraumatic stress disorder
RA	Research Assistant
RAB	Research Advisory Board
RCT	randomized clinical trial
SAE	serious adverse event
SAP	Statistical Analysis Plan
SDCC	Statistics and Data Coordinating Center
SFC	San Fernando Health Center

SRG	Survey Research Group
SRMC	Sandoval Regional Medical Center
SUMMIT	Substance Use Motivation and Medication Integrated Treatment
UNM	University of New Mexico
UP	unanticipated problem
ViStA	Violence and Stress Assessment
VR-12	Veterans RAND 12-item Health Survey
WET	Written Exposure Therapy

10.3 PROTOCOL AMENDMENT HISTORY

The table below is intended to capture changes of IRB-approved versions of the protocol, including a description of the change and rationale. A ***Summary of Changes*** *table for the current amendment is located in the* ***Protocol Title Page****.*

Version	Date	Description of Change	Brief Rationale
2.0	10/12/20	We amended the Protocol in major ways to include the activities which will occur as part of our newly funded Competitive Revision (CR). The amended protocol additionally includes some minor changes to the parent study. We include the CR in the amended protocol under guidance from our project officer, Dr. Michael Freed and because the CR and the parent study are operationally tied together. Thus, for NIMH DSMB review, we have provided one unified protocol that includes all components.	There are 3 main reasons for these changes: (1) to add information about the CR, (2) to insure compatibility with changes requested from RAND's Institutional Review Board, and (3) because additional discussion among our team led to greater clarity in describing procedures.
3.0	2/3/21	We responded to queries about circumstances warranting termination (#4 in memo), stopping rules (#6 in memo), and protocol deviations/violations (#9 in memo). We also added a paragraph explaining how the PHQ works across the eligibility screener and the baseline interview.	To be responsive to the board and to describe a change in screening that was approved by Dr. Freed.
4.0	3/15/21	We made changes to the Statistical Considerations section (section 9) in accordance with DSMB queries about statistical power and other points of analysis. We clarified language around reporting of AEs, SAEs, protocol deviations, and unanticipated problems. We added additional detail about Care Coordinator training and supervision, updated the eligibility criteria to indicate that participants cannot be receiving both MOUD and psychotropic medication from an external provider prior to study enrollment, and updated the	Most changes in this amendment were made in response to DMSB queries or to increase internal consistency and clarity of procedures described in the protocol. Eligibility criteria were updated to ensure that participating patients are in a position to receive COD care coordination at their primary care clinic. We changed the PHQ-9 to the PHQ-8 at eligibility screening to avoid asking for information that could require us to break confidentiality before a

		eligibility screener to include the PHQ-8 rather than PHQ-9. Finally, we made some minor wording changes to maintain internal consistency within the protocol.	participant has completed the informed consent process.
5.0	6/24/21	We added First Choice Alameda to tables 5.1 and 5.3 of clinic-level characteristics/data. Please note that at this time we are still waiting for our clinic partners to provide us with some of the specific site characteristics, so some fields for Alameda are blank. We changed the number of clinics from 13 to 14.	The study added a new site, First Choice Alamedia to the trial.
6.0	12/9/21	We updated throughout to indicate that we have two more clinics in a new health system, CSV: Baker Street Village and Kern River. We increased the number of clinics from 14 to 16 throughout. We removed MDD and PTSD remission/response, opioid overdose risk behaviors, and general health functioning outcomes as secondary outcomes.	To boost recruitment, we have added a new health system in central California – Clinica Sierra Vista. We may add more CSV sites after we onboard the first two. We revised the outcomes to boost power.
6.1	1/26/22	We changed the number of clinics from 14 to 16 and added a new health system, Clinica Sierra Vista.	We did this to boost recruitment.
6.2	3/22/22	We removed Clinica Sierra Vista and added two new health systems in Los Angeles County, California (Providence & HH).<br><br>We doubled the patient incentive amount for the baseline survey and added a $25 bonus incentive to encourage same day or call-back later completion.	These new health systems were recently engaged to replace the systems we lost.<br><br>To boost patient enrollment.
6.3	5/27/22	We updated the study timeline.<br><br>We added information explaining the addition of the California clinics.<br><br>We added information about the process for reportable events at	To account for recruitment challenges and addition of new clinical sites.<br><br>To provide a rationale for our selected study sites.<br><br>Changes made per the request of partnering California clinics.

		participating California clinics, and we made other minor changes to clarify information for the new California sites.<br><br>We updated Dr. Cristina Murray-Krezan's institutional affiliation to be the University of Pittsburgh.	 <br><br><br>Dr. Murray-Krezan moved from UNM to the University of Pittsburgh.
7.0	7/29/22	We added information detailing RAND taking over data collection from UNM.<br><br>The parent and supplement (CLARO+) consents (dated 7/21/2022) were updated with new contact information to reflect the SRG transition and allow the SRG to receive patient contact information, as well as added language clarifying which study incentives may be in the form of cash and which may be in the form of a merchandise card. Additionally, a sentence specifying that the study may collect mortality data from the state vital records office was added, which will allow us to collect mortality data from California. The protocol contained all of these changes, as well as a few minor staffing changes and a sentence on the patient demographics at the Providence clinics.	To provide details on the transition from UNM to RAND in anticipation of the change in data collection activities
8.0	10/5/22	Added details on the transition from parent to supplement at various clinics	To account for updates in recruitment status and provide a realistic explanation of transitioning from CLARO to CLARO+
9.0	10/11/22	Added Mid Valley Comprehensive Health Center and San Fernando Health Center to the study, clarified the parent system for these new clinics, and rectified some mislabeling of clinic numbers from previous iterations of the protocol	To boost recruitment
10.0	01/13/23	Changed name of PSJ to Providence Health & Services– Southern California	To include a wider net of PSJ clinics and hopefully boost recruitment.

11.0	05/26/23	End of recruitment at PSJ was noted. We also noted that some patients who are receiving primary care at UNM may be referred to the study from UNM providers outside of the three UNM clinics.<br>Changed duration of study recruitment period to reflect extended recruitment period.	To transition study resources to a site that will likely be more productive for recruitment and enrollment, and to increase potential for study recruitment.<br>To provide additional time to meet the target sample size.
12.0	06/14/23	JWCH Institute Inc. was added as a participating healthcare system, and end of recruitment at PSJ was noted.	To transition study resources to a site that will likely be more productive for recruitment and enrollment, and to increase potential for study recruitment.
13.0	6/27/23	Added Sandoval Regional Medical Center (SRMC) as an additional recruitment site in New Mexico. SRMC is part of the UNM system.	To increase potential for study recruitment.

2153

11 REFERENCES

1. Adam A, Schwartz RP, Wu L-T, et al. Electronic self-administered screening for substance use in adult primary care patients: Feasibility and acceptability of the tobacco, alcohol, prescription medication, and other substance use (myTAPS) screening tool. *Addiction Science & Clinical Practice*. 2019;14(1):39.

2. Spitzer RL, Kroenke K, Williams JB. Validation and utility of a self-report version of PRIME-MD: The PHQ primary care study. Primary care evaluation of mental disorders. Patient health questionnaire. *Jama*. Nov 10 1999;282(18):1737-44.

3. Prins A, Bovin MJ, Smolenski DJ, et al. The primary care PTSD screen for DSM-5 (PC-PTSD-5): Development and evaluation within a veteran primary care sample. *Journal of General Internal Medicine*. 2016;31(10):1206-1211.

4. Centers for Medicare & Medicaid Services MIT. Continuity of pharmacotherapy for opioid use disorder (OUD). Accessed June 9, 2020, https://cmit.cms.gov/CMIT_public/ViewMeasure?MeasureId=5881

5. Thomas CP, Garnick DW, Horgan CM, Miller K, Harris AH, Rosen MM. Establishing the feasibility of measuring performance in use of addiction pharmacotherapy. *J Subst Abuse Treat*. Jul 2013;45(1):11-8. doi:10.1016/j.jsat.2013.01.004

6. Substance Abuse and Mental Health Services Administration. *National Survey on Drug Use and Health (NSDUH), 2019 (Final CAI Specifications for Programming, English version)*. 2019.

7. Pilkonis PA, Yu L, Dodds NE, et al. Item banks for substance use from the Patient-Reported Outcomes Measurement Information System (PROMIS(®)): Severity of use and positive appeal of use. *Drug and Alcohol Dependence*. 2015;156:184-192.

8. Babor TF, Higgins-Biddle JC, Robaina K. *USAUDIT: The Alcohol Use Disorders Identification Test, Adapted for Use in the United States: A Guide for Primary Care Practitioners*. 2016. Accessed June 9, 2020. https://www.dshs.wa.gov/sites/default/files/BHSIA/dbh/wasbirt/USAUDIT-Guide_2016.pdf

9. Bohnert AS, Bonar EE, Cunningham R, et al. A pilot randomized clinical trial of an intervention to reduce overdose risk behaviors among emergency department patients at risk for prescription opioid overdose. *Drug and alcohol dependence*. 2016;163:40-47.

10. Open Society Foundations. Overdose baseline questionnaire, Naloxone info. Accessed March 18, 2019, http://www.naloxoneinfo.org/sites/default/files/OD%20Baseline_Questionnaire%20ENG.pdf

11. Posner K, Brown GK, Stanley B, et al. The Columbia–Suicide Severity Rating Scale: Initial validity and internal consistency findings from three multisite studies with adolescents and adults. *American Journal of Psychiatry*. 2011;168(12):1266-1277.

12. National Committee for Quality Assurance (NCQA). Initiation and engagement of alcohol and other drug abuse or dependence treatment (IET). Accessed June 9, 2020, https://www.ncqa.org/hedis/measures/initiation-and-engagement-of-alcohol-and-other-drug-abuse-or-dependence-treatment/

13. Hepner KA, Roth CP, Sloss EM, Paddock SM, Iyiewuare PI, Timmer M, Pincus HA. *Quality of care for PTSD and depression in the Military Health System: Final report (RR-1542-OSD)*. 2017.

14. Selim AJ, Rogers W, Fleishman JA, Qian SX, Fincke BG, Rothendler JA, Kazis LE. Updated U.S. population standard for the Veterans RAND 12-item Health Survey (VR-12). *Qual Life Res*. Feb 2009;18(1):43-52. doi:10.1007/s11136-008-9418-2

15. Krebs EE, Lorenz KA, Bair MJ, et al. Development and initial validation of the PEG, a three-item scale assessing pain intensity and interference. *Journal of General Internal Medicine*. 2009;24(6):733-738.

16. Montgomery AE, Fargo JD, Byrne TH, Kane VR, Culhane DP. Universal screening for homelessness and risk for homelessness in the Veterans Health Administration. *American Journal of Public Health*. 2013;103(S2):S210-S211.

17. Substance Abuse and Mental Health Services Administration. Government performance and results act (GPRA). Client outcome measures for discretionary programs. Question-by-question instruction guide, version 9.6. Accessed June 9, 2020, https://www.samhsa.gov/sites/default/files/GPRA/SAIS_GPRA_Services_Tool_QxQ_final.pdf

18. Leonhard C, Mulvey K, Gastfriend DR, Shwartz M. The Addiction Severity Index: A field study of internal consistency and validity. *Journal of Substance Abuse Treatment*. 2000;18(2):129-135.

19. Leon AC, Olfson M, Portera L, Farber L, Sheehan DV. Assessing psychiatric impairment in primary care with the Sheehan Disability Scale. *The International Journal of Psychiatry in Medicine*. 1997;27(2):93-105.

20. United States Department of Agriculture. Rural-urban commuting area codes. Accessed June 9, 2020, https://www.ers.usda.gov/data-products/rural-urban-commuting-area-codes/

21. Agency for Healthcare Research and Quality. CAHPS ECHO survey measures. Accessed March 18, 2019, http://www.ahrq.gov/cahps/surveys-guidance/echo/about/survey-measures.html

22. Sturgiss EA, Rieger E, Haesler E, Ridd MJ, Douglas K, Galvin SL. Adaption and validation of the Working Alliance Inventory for General Practice: Qualitative review and cross-sectional surveys. *Family Practice*. 2019;36(4):516-522.

23. Olfson M, Rossen LM, Wall MM, Houry D, Blanco C. Trends in intentional and unintentional opioid overdose deaths in the United States, 2000-2017. *Jama*. 2019;322(23):2340-2342.

24. Bohnert AS, Ilgen MA. Understanding links among opioid use, overdose, and suicide. *New England journal of medicine*. 2019;380(1):71-79.

25. Bohnert KM, Ilgen MA, Louzon S, McCarthy JF, Katz IR. Substance use disorders and the risk of suicide mortality among men and women in the US Veterans Health Administration. *Addiction*. 2017;112(7):1193-1201.

26. Centers for Disease Control and Prevention. CDC WONDER. Accessed March 13, 2020, https://wonder.cdc.gov/

27. Darke S, Ross J, Lynskey M, Teesson M. Attempted suicide among entrants to three treatment modalities for heroin dependence in the Australian Treatment Outcome Study (ATOS): Prevalence and risk factors. *Drug Alcohol Depend*. Jan 7 2004;73(1):1-10.

28. Oquendo MA, Volkow ND. Suicide: A silent contributor to opioid-overdose deaths. *New England Journal of Medicine*. 2018;378(17):1567-1569.

29. Warner-Smith M, Darke S, Lynskey M, Hall W. Heroin overdose: Causes and consequences. *Addiction*. 2001;96(8):1113-1125.

30. Wilcox HC, Conner KR, Caine ED. Association of alcohol and drug use disorders and completed suicide: An empirical review of cohort studies. *Drug and Alcohol Dependence*. 2004;76:S11-S19.

31. Ilgen MA, Bohnert AS, Ignacio RV, McCarthy JF, Valenstein MM, Kim HM, Blow FC. Psychiatric diagnoses and risk of suicide in veterans. *Archives of general psychiatry*. 2010;67(11):1152-1158.

32. Mościcki E, O'Carroll P, Rae D, Locke B, Roy A, Regier D. Suicide attempts in the Epidemiologic Catchment Area study. *The Yale Journal of Biology and Medicine*. 1988;61(3):259-268.

33. Fendrich M, Becker J, Hernandez-Meier J. Psychiatric symptoms and recent overdose among people who use heroin or other opioids: Results from a secondary analysis of an intervention study. *Addictive Behaviors Reports*. 2019;10:100212.

34. Jones CM, McCance-Katz EF. Co-occurring substance use and mental disorders among adults with opioid use disorder. *Drug Alcohol Depend*. Feb 14 2019;197:78-82. doi:10.1016/j.drugalcdep.2018.12.030

35. Darke S, Ross J, Marel C, Mills KL, Slade T, Burns L, Teesson M. Patterns and correlates of attempted suicide amongst heroin users: 11-year follow-up of the Australian treatment outcome study cohort. *Psychiatry research*. 2015;227(2-3):166-170.

36. Barry DT, Cutter CJ, Beitel M, Kerns RD, Liong C, Schottenfeld RS. Psychiatric disorders among patients seeking treatment for co-occurring chronic pain and opioid use disorder. *The Journal of clinical psychiatry*. Oct 2016;77(10):1413-1419. doi:10.4088/JCP.15m09963

37. Compton WM, Thomas YF, Stinson FS, Grant BF. Prevalence, correlates, disability, and comorbidity of DSM-IV drug abuse and dependence in the United States: Results from the national epidemiologic survey on alcohol and related conditions. *Arch Gen Psychiatry*. May 2007;64(5):566-76. doi:doi: 10.1001/archpsyc.64.5.566

38. Dore G, Mills K, Murray R, Teesson M, Farrugia P. Post-traumatic stress disorder, depression and suicidality in inpatients with substance use disorders. *Drug and Alcohol Review*. 2012;31(3):294-302.

39. Grant BF, Stinson FS, Dawson DA, et al. Prevalence and co-occurrence of substance use disorders and independent mood and anxiety disorders: Results from the National Epidemiologic Survey on Alcohol and Related Conditions. *Archives of General Psychiatry*. 2004;61(8):807-816. doi:10.1001/archpsyc.61.8.807

40. Han B, Compton WM, Blanco C, Colpe LJ. Prevalence, treatment, and unmet treatment needs of US adults with mental health and substance use disorders. *Health affairs (Project Hope)*. Oct 1 2017;36(10):1739-1747. doi:10.1377/hlthaff.2017.0584

41. Meier A, Lambert-Harris C, McGovern MP, Xie H, An M, McLeman B. Co-occurring prescription opioid use problems and posttraumatic stress disorder symptom severity. *The American Journal of Drug and Alcohol Abuse*. 2014/07/01 2014;40(4):304-311. doi:10.3109/00952990.2014.910519

42. Mills KL, Teesson M, Ross J, Peters L. Trauma, PTSD, and substance use disorders: Findings from the Australian national survey of mental health and well-being. *American Journal of Psychiatry*. 2006;163(4):652-658.

43. Ahmadi J, Jahromi MS, Ehsaei Z. The effectiveness of different singly administered high doses of buprenorphine in reducing suicidal ideation in acutely depressed people with co-morbid opiate dependence: A randomized, double-blind, clinical trial. *Trials*. 2018;19(1):462.

44. Ahmadi J, Sarani EM, Jahromi MS. Rapid effect of a single-dose buprenorphine on reduction of opioid craving and suicidal ideation: A randomized, double blind, placebo-controlled study. *Ci Ji Yi Xue Za Zhi*. Jan-Mar 2020;32(1):58-64. doi:10.4103/tcmj.tcmj_220_18

45. Gordon AJ, Lo-Ciganic W-H, Cochran G, Gellad WF, Cathers T, Kelley D, Donohue JM. Patterns and quality of buprenorphine opioid agonist treatment in a large Medicaid program. *Journal of addiction medicine*. 2015;9(6):470-477.

46. Morgan JR, Schackman BR, Leff JA, Linas BP, Walley AY. Injectable naltrexone, oral naltrexone, and buprenorphine utilization and discontinuation among individuals treated for opioid use disorder in a United States commercially insured population. *J Subst Abuse Treat*. Feb 2018;85:90-96. doi:10.1016/j.jsat.2017.07.001

47. Samples H, Williams AR, Olfson M, Crystal S. Risk factors for discontinuation of buprenorphine treatment for opioid use disorders in a multi-state sample of Medicaid enrollees. *J Subst Abuse Treat*. Dec 2018;95:9-17. doi:10.1016/j.jsat.2018.09.001

48. Williams AR, Samples H, Crystal S, Olfson M. Acute care, prescription opioid use, and overdose following discontinuation of long-term buprenorphine treatment for opioid use disorder. *American Journal of Psychiatry*. 2020;177(2):117-124.

49. Harris KM, Edlund MJ. Use of mental health care and substance abuse treatment among adults with co-occurring disorders. *Psychiatric Services*. 2005;56(8):954-959.

50. Priester MA, Browne T, Iachini A, Clone S, DeHart D, Seay KD. Treatment access barriers and disparities among individuals with co-occurring mental health and substance use disorders: An integrative literature review. *Journal of Substance Abuse Treatment*. 2016;61:47-59. doi:10.1016/j.jsat.2015.09.006

51. U.S. Department of Health and Human Services. *Report to Congress on the prevention and treatment of co-occurring substance abuse disorders and mental disorders*. 2002.

52. Kirby KC, Versek B, Kerwin ME, et al. Developing Community Reinforcement and Family Training (CRAFT) for parents of treatment-resistant adolescents. *Journal of Child & Adolescent Substance Abuse*. 2015;24(3):155-165.

53. Manuel JK, Austin JL, Miller WR, et al. Community Reinforcement and Family Training: A pilot comparison of group and self-directed delivery. *J Subst Abuse Treat*. 2012;43(1):129-136.

54. Meyers RJ, Miller WR, Smith JE, Tonigan JS. A randomized trial of two methods for engaging treatment-refusing drug users through concerned significant others. *Journal of Consulting and Clinical Psychology*. 2002;70(5):1182-1185.

55. Miller WR, Meyers RJ, Tonigan JS. Engaging the unmotivated in treatment for alcohol problems: A comparison of three strategies for intervention through family members. *Journal of Consulting and Clinical Psychology*. 1999;67(5):688-697.

56. Osilla KC, Trail TE, Pedersen ER, Gore KL, Tolpadi A, Rodriguez LM. Efficacy of a web-based intervention for concerned spouses of service members and veterans with alcohol misuse. *Journal of Marital and Family Therapy*. 2017;

57. Rodriguez LM, Neighbors C, Osilla KC, Trail TE. The longitudinal effects of military spouses' concern and behaviors over partner drinking on relationship functioning. *Alcohol*. May 2019;76:29-36. doi:10.1016/j.alcohol.2018.07.004

58. Roozen HG, de Waart R, van der Kroft P. Community reinforcement and family training: An effective option to engage treatment-resistant substance-abusing individuals in treatment. *Addiction*. 2010;105(10):1729-1738.

59. Trail TE, Osilla KC, Rodriguez LM, Pedersen ER, Gore KL. Exploring the association between changes in partner behaviors, perceived service member drinking, and relationship quality: Secondary analysis of a web-based intervention for military partners. *J Subst Abuse Treat*. Mar 2019;98:66-72. doi:10.1016/j.jsat.2019.01.002

60. Waldron HB, Kern-Jones S, Turner CW, Peterson TR, Ozechowski TJ. Engaging resistant adolescents in drug abuse treatment. *J Subst Abuse Treat*. 2007;32(2):133-142.

61. Brigham GS, Slesnick N, Winhusen TM, Lewis DF, Guo X, Somoza E. A randomized pilot clinical trial to evaluate the efficacy of Community Reinforcement and Family Training for Treatment Retention (CRAFT-T) for improving outcomes for patients completing opioid detoxification. *Drug Alcohol Depend*. 2014;138:240-243.

62. Novak P, Feder KA, Ali MM, Chen J. Behavioral health treatment utilization among individuals with co-occurring opioid use disorder and mental illness: Evidence from a national survey. *J Subst Abuse Treat*. Mar 2019;98:47-52. doi:10.1016/j.jsat.2018.12.006

63. Brown PJ, Recupero PR, Stout R. PTSD substance abuse comorbidity and treatment utilization. *Addictive behaviors*. Mar-Apr 1995;20(2):251-4.

64. Kessler RC, Sonnega A, Bromet E, Hughes M, Nelson CB. Posttraumatic stress disorder in the National Comorbidity Survey. *Archives of general psychiatry*. 1995;52(12):1048-1060.

65. Mills KL, Teesson M, Ross J, Darke S, Shanahan M. The costs and outcomes of treatment for opioid dependence associated with posttraumatic stress disorder. *Psychiatric services (Washington, DC)*. Aug 2005;56(8):940-5. doi:10.1176/appi.ps.56.8.940

66. Nunes EV, Sullivan MA, Levin FR. Treatment of depression in patients with opiate dependence. *Biological psychiatry*. Nov 15 2004;56(10):793-802. doi:10.1016/j.biopsych.2004.06.037

67. Ouimette PC, Ahrens C, Moos RH, Finney JW. Posttraumatic stress disorder in substance abuse patients: Relationship to 1-year posttreatment outcomes. *Psychology of Addictive Behaviors*. 1997;11(1):34.

68. Ouimette PC, Brown PJ, Najavits LM. Course and treatment of patients with both substance use and posttraumatic stress disorders. *Addictive behaviors*. 1998;23(6):785-795.

69. Centers for Disease Control. Drug overdose deaths. Accessed March 4, 2019, *https://www.cdc.gov/drugoverdose/data/statedeaths.html*

70. National Institute of Mental Health. U.S. leading categories of diseases/disorders. Accessed March 19, 2019, *https://www.nimh.nih.gov/health/statistics/disability/us-leading-categories-of-diseases-disorders.shtml*

71. Fudala PJ, Bridge TP, Herbert S, et al. Office-based treatment of opiate addiction with a sublingual-tablet formulation of buprenorphine and naloxone. *New England Journal of Medicine*. 2003;349(10):949-958. doi:doi:10.1056/NEJMoa022164

72. Knudsen HK, Abraham AJ, Oser CB. Barriers to the implementation of medication-assisted treatment for substance use disorders: the importance of funding policies and medical infrastructure. *Eval Program Plann*. Nov 34(4):375-81. doi:S0149-7189(11)00016-4 [pii]

10.1016/j.evalprogplan.2011.02.004 [doi]

73. Saitz R, Daaleman TP. Now is the time to address substance use disorders in primary care. *Annals of family medicine*. Jul 2017;15(4):306-308. doi:10.1370/afm.2111

74. Appel PW, Ellison AA, Jansky HK, Oldak R. Barriers to enrollment in drug abuse treatment and suggestions for reducing them: Opinions of drug injecting street outreach clients and other system stakeholders. *The American Journal of Drug and Alcohol Abuse*. 2004;30(1):129-153.

75. Cunningham JA, Sobell LC, Sobell MB, Agrawal S, Toneatto T. Barriers to treatment: Why alcohol and drug abusers delay or never seek treatment. *Addictive behaviors*. 1993;18(3):347-353.

76. Beronio K, Po R, Skopec L. Affordable Care Act will expand mental health and substance use disorder benefits and parity protections for 62 million Americans. ASPE Research Brief. Department of Health and Human Services. Accessed February 20, 2013. http://aspe.hhs.gov/health/reports/2013/mental/rb_mental.cfm

77. Buck JA. The looming expansion and transformation of public substance abuse treatment under the Affordable Care Act. *Health affairs (Project Hope)*. Aug 2011;30(8):1402-10. doi:10.1377/hlthaff.2011.0480

78. Cherpitel CJ, Ye Y. Trends in alcohol- and drug-related emergency department and primary care visits: Data from four U.S. national surveys (1995-2010). *Journal of Studies on Alcohol and Drugs*. 2012;73(3):454-458.

79. Pilowsky DJ, Wu LT. Screening for alcohol and drug use disorders among adults in primary care: A review. *Journal of Substance Abuse and Rehabilitation*. 2012;3(1):25-34.

80. Alexander CL, Arnkoff DB, Glass CR. Bringing psychotherapy to primary care: Innovations and challenges. *Clinical Psychology: Science and Practice*. 2010;17(3):191-214.

81. Rosenbaum S, Paradise J, Markus A, Sharac J, Tran C, Reynolds D, Shin P. *Issue brief: Community health centers: Recent growth and the role of the ACA*. 2017.

82. Trust for America's Health. *Pain in the nation: The drug, alcohol and suicide crises and the need for a national resilience strategy*. 2017. http://www.paininthenation.org/assets/pdfs/TFAH-2017-PainNationRpt.pdf

83. New Mexico Department of Health. How New Mexico compares, 2018. Accessed March 19, 2019, https://nmhealth.org/publication/view/general/4471/

84. United States Census Bureau. American community survey (ACS). Accessed March 4, 2019, https://www.census.gov/programs-surveys/acs/about.html

85. Henry J. Kaiser Family Foundation. Primary care health professional shortage areas (HPSAs). Accessed March 19, 2019, https://www.kff.org/other/state-indicator/primary-care-health-professional-shortage-areas-hpsas/?currentTimeframe=0&sortModel=%7B%22colId%22:%22Location%22,%22sort%22:%22asc%22%7D

86. Rural Health Information Hub. New Mexico chart gallery. Accessed March 21, 2019, https://www.ruralhealthinfo.org/charts/5?state=NM

87. New Mexico Department of Health. New Mexico substance abuse epidemiology profile. Accessed March 19, 2019, https://nmhealth.org/data/view/substance/2067/

88. New Mexico’s Indicator Based Information System (NM-IBIS). Complete health indicator report. Accessed March 19, 2019, https://ibis.health.state.nm.us/indicator/complete_profile/DrugOverdoseDth.html

89. New Mexico Health and Human Services Department. Mental health parity report for Medicaid managed care in NM. Accessed March 19, 2019, http://www.hsd.state.nm.us/LookingForInformation/medicaid-eligibility.aspx

90. Substance Abuse and Mental Health Services Administration. Buprenephrine treatment practitioner locator. Accessed March 19, 2019, https://www.samhsa.gov/medication-assisted-treatment/physician-program-data/treatment-physician-locator

91. Heinzerling KG, Ober AJ, Lamp K, De Vries D, Watkins KE. SUMMIT: Procedures for medication-assisted treatment of alcohol or opioid dependence in primary care (TL-148-NIDA). RAND Corporation. Accessed June 16, 2016. http://www.rand.org/pubs/tools/TL148.html

92. Osilla KC, D'Amico EJ, Lind M, Ober AJ, Watkins KE. Brief treatment for substance use disorders: A guide for behavioral health providers (TL-147-NIDA). RAND Corporation. Accessed June 16, 2016. http://www.rand.org/pubs/tools/TL147.html

93. Schackman BR, Leff JA, Polsky D, Moore BA, Fiellin DA. Cost-effectiveness of long-term outpatient buprenorphine-naloxone treatment for opioid dependence in primary care. *Journal of General Internal Medicine*. 2012;27(6):669-676.

94. Substance Abuse and Mental Health Services Administration. *National Survey of Substance Abuse Treatment Services (N-SSATS): 2011. Data on substance abuse treatment facilities*. 2012.

95. Center for Behavioral Health Statistics and Quality. Key substance use and mental health indicators in the United States: Results from the 2015 national survey on drug use and health. HHS publication no. SMA 16-4984, NSDUH series H-51. Substance Abuse and Mental Health Services Administration. Accessed February 1, 2019, https://www.samhsa.gov/data/sites/default/files/NSDUH-FFR1-2015/NSDUH-FFR1-2015/NSDUH-FFR1-2015.pdf

96. American Academy of Otolaryngology - Head and Neck Surgery. Quality ID #468 (NQF 3175): Continuity of pharmacotherapy for opioid use disorder (OUD). Accessed March 19, 2019, https://www.entnet.org/sites/default/files/uploads/PracticeManagement/Resources/_files/2019_measure_468_mipscqm.pdf

97. Bentzley BS, Barth KS, Back SE, Book SW. Discontinuation of buprenorphine maintenance therapy: Perspectives and outcomes. *J Subst Abuse Treat*. May 2015;52:48-57. doi:10.1016/j.jsat.2014.12.011

98. Archer J, Bower P, Gilbody S, et al. Collaborative care for depression and anxiety problems. *The Cochrane database of systematic reviews*. Oct 17 2012;10:Cd006525. doi:10.1002/14651858.CD006525.pub2

99. Huang Y, Wei X, Wu T, Chen R, Guo A. Collaborative care for patients with depression and diabetes mellitus: A systematic review and meta-analysis. *BMC psychiatry*. Oct 14 2013;13:260. doi:10.1186/1471-244x-13-260

100. Huffman JC, Niazi SK, Rundell JR, Sharpe M, Katon WJ. Essential articles on collaborative care models for the treatment of psychiatric disorders in medical settings: A publication by the Academy of Psychosomatic Medicine Research and Evidence-Based Practice Committee. *Psychosomatics*. Mar-Apr 2014;55(2):109-22. doi:10.1016/j.psym.2013.09.002

101. Wakeman SE, Kanter GP, Donelan K. Institutional substance use disorder intervention improves general internist preparedness, attitudes, and clinical practice. *J Addict Med*. Jul/Aug 2017;11(4):308-314. doi:10.1097/adm.0000000000000314

102. Tully PJ, Baumeister H. Collaborative care for comorbid depression and coronary heart disease: A systematic review and meta-analysis of randomised controlled trials. *BMJ open*. Dec 21 2015;5(12):e009128. doi:10.1136/bmjopen-2015-009128

103. Arean PA, Ayalon L, Hunkeler E, et al. Improving depression care for older, minority patients in primary care. *Medical care*. Apr 2005;43(4):381-90.

104. Ell K, Aranda MP, Xie B, Lee PJ, Chou CP. Collaborative depression treatment in older and younger adults with physical illness: Pooled comparative analysis of three randomized clinical trials. *The American journal of geriatric psychiatry : official journal of the American Association for Geriatric Psychiatry*. Jun 2010;18(6):520-30. doi:10.1097/JGP.0b013e3181cc0350

105. Ell K, Katon W, Cabassa LJ, Xie B, Lee PJ, Kapetanovic S, Guterman J. Depression and diabetes among low-income Hispanics: Design elements of a socioculturally adapted collaborative care model randomized controlled trial. *International journal of psychiatry in medicine*. 2009;39(2):113-32. doi:10.2190/PM.39.2.a

106. Ell K, Katon W, Xie B, Lee PJ, Kapetanovic S, Guterman J, Chou CP. Collaborative care management of major depression among low-income, predominantly Hispanic subjects with diabetes: A randomized controlled trial. *Diabetes care*. Apr 2010;33(4):706-13. doi:10.2337/dc09-1711

107. Miranda J, Duan N, Sherbourne C, Schoenbaum M, Lagomasino I, Jackson-Triche M, Wells KB. Improving care for minorities: Can quality improvement interventions improve care and outcomes for depressed minorities? Results of a randomized, controlled trial. *Health services research*. Apr 2003;38(2):613-30.

108. Miranda J, Schoenbaum M, Sherbourne C, Duan N, Wells K. Effects of primary care depression treatment on minority patients' clinical status and employment. *Arch Gen Psychiatry*. Aug 2004;61(8):827-34. doi:10.1001/archpsyc.61.8.827

109. Watkins KE, Ober AJ, Lamp K, et al. Implementing the chronic care model for opioid and alcohol use disorders in primary care. *Progress in Community Health Partnerships: Research, Education, and Action*. 2017;11(4):397-407.

110. Watkins KE, Ober AJ, Lamp K, et al. Collaborative care for opioid and alcohol use disorders in primary care: The SUMMIT randomized clinical trial. *JAMA internal medicine*. Oct 1 2017;177(10):1480-1488. doi:10.1001/jamainternmed.2017.3947

111. Hunt P, Ober AJ, Watkins KE. *The costs of implementing collaborative care for opioid and alcohol use disorders in primary care (RR-2049-NIDA)*. 2017. https://www.rand.org/pubs/research_reports/RR2049.html

112. Hunter SB, Ober AJ, McCullough CM, Storholm ED, Iyiewuare PO, Pham C, Watkins KE. Sustaining alcohol and opioid use disorder treatment in primary care: A mixed methods study. *Implementation Science*. 2018;13:83.

113. Ober AJ, Watkins KE, Hunter SB, et al. Assessing and improving organizational readiness to implement substance use disorder treatment in primary care: Findings from the SUMMIT study. *BMC family practice*. Dec 21 2017;18(1):107. doi:10.1186/s12875-017-0673-6

114. Wells KB, Sherbourne C, Schoenbaum M, et al. Impact of disseminating quality improvement programs for depression in managed primary care: A randomized controlled trial. *Jama*. 2000;283(2):212-220.

115. Meredith LS, Jackson-Triche M, Duan N, Rubenstein LV, Camp P, Wells KB. Quality improvement for depression enhances long-term treatment knowledge for primary care clinicians. *Journal of General Internal Medicine*. 2000;15(12):868-877.

116. Rost KM, Duan N, Rubenstein LV, Ford DE, Sherbourne CD, Meredith LS, Wells KB. The Quality Improvement for Depression collaboration: general analytic strategies for a coordinated study of quality improvement in depression care. *General hospital psychiatry*. Sep-Oct 2001;23(5):239-53.

117. Rubenstein LV, Meredith LS, Parker LE, Gordon NP, Hickey SC, Oken C, Lee ML. Impacts of evidence-based quality improvement on depression in primary care: A randomized experiment. *J Gen Intern Med*. Oct 2006;21(10):1027-35. doi:10.1111/j.1525-1497.2006.00549.x

118. Watkins KE, Paddock SM, Zhang L, Wells KB. Improving care for depression in patients with comorbid substance misuse. *The American journal of psychiatry*. Jan 2006;163(1):125-32. doi:10.1176/appi.ajp.163.1.125

119. Morton I, Hurley B, Castillo E, et al. Outcomes of two quality improvement depression care interventions in individuals with substance use problems. *The American Journal of Drug and Alcohol Abuse*. 2020;46(2):251-261.

120. Meredith LS, Eisenman DP, Han B, et al. Impact of collaborative care for underserved patients with PTSD in primary care: A randomized controlled trial. *Journal of General Internal Medicine*. 2016;31:509-517.

121. Iyiewuare PO, McCullough C, Ober A, Becker K, Osilla K, Watkins KE. Demographic and mental health characteristics of individuals who present to community health clinics with substance misuse. *Health services research and managerial epidemiology*. Jan-Dec 2017;4:2333392817734523. doi:10.1177/2333392817734523

122. Storholm ED, Ober AJ, Hunter SB, Becker KM, Iyiewuare PO, Pham C, Watkins KE. Barriers to integrating the continuum of care for opioid and alcohol use disorders in primary care: A qualitative longitudinal study. *J Subst Abuse Treat*. Dec 2017;83:45-54. doi:10.1016/j.jsat.2017.09.015

123. Sturgiss EA, Sargent GM, Haesler E, Rieger E, Douglas K. Therapeutic alliance and obesity management in primary care - a cross-sectional pilot using the Working Alliance Inventory. *Clinical obesity*. Dec 2016;6(6):376-379. doi:10.1111/cob.12167

124. Ferreira PH, Ferreira ML, Maher CG, Refshauge KM, Latimer J, Adams RD. The therapeutic alliance between clinicians and patients predicts outcome in chronic low back pain. *Physical therapy*. Apr 2013;93(4):470-8. doi:10.2522/ptj.20120137

125. Stewart M. Reflections on the doctor-patient relationship: From evidence and experience. *The British journal of general practice : the journal of the Royal College of General Practitioners*. Oct 2005;55(519):793-801.

126. Coventry PA, Hudson JL, Kontopantelis E, et al. Characteristics of effective collaborative care for treatment of depression: A systematic review and meta-regression of 74 randomised controlled trials. *PloS one*. 2014;9(9):e108114. doi:10.1371/journal.pone.0108114

127. Gilbody S, Bower P, Whitty P. Costs and consequences of enhanced primary care for depression: Systematic review of randomised economic evaluations. *The British journal of psychiatry : the journal of mental science*. Oct 2006;189:297-308. doi:10.1192/bjp.bp.105.016006

128. Anhang Price R, Elliott MN, Zaslavsky AM, et al. Examining the role of patient experience surveys in measuring health care quality. *Medical care research and review : MCRR*. Oct 2014;71(5):522-54. doi:10.1177/1077558714541480

129. Davenport S, Matthews K. *Milliman White Paper: Opioid use disorder in the United States: Diagnosed prevalence by payer, age, sex, and state*. 2018. Accessed July 7, 2018. http://www.milliman.com/uploadedFiles/insight/2018/Opioid_Use_Disorder_Prevalence.pdf

130. Center for Behavioral Health Statistics and Quality (CBHSQ). Results from the 2015 National Survey on Drug Use and Health: detailed tables. Accessed July 6, 2018, http://www.samhsa.gov/data/sites/default/files/NSDUH-DetTabs-2015/NSDUH-DetTabs-2015/NSDUH-DetTabs-2015.pdf

131. Wu L-T, Zhu H, Swartz MS. Treatment utilization among persons with opioid use disorder in the United States. *Drug and alcohol dependence*. 10/19 2016;169:117-127. doi:10.1016/j.drugalcdep.2016.10.015

132. Tremeau F, Darreye A, Staner L, Correa H, Weibel H, Khidichian F, Macher JP. Suicidality in opioid-dependent subjects. *The American journal on addictions*. May-Jun 2008;17(3):187-94. doi:10.1080/10550490802020160

133. Turner BJ, Liang Y. Drug overdose in a retrospective cohort with non-cancer pain treated with opioids, antidepressants, and/or sedative-hypnotics: interactions with mental health disorders. *Journal of general internal medicine*. 2015;30(8):1081-1096.

134. Bell J, Trinh L, Butler B, Randall D, Rubin G. Comparing retention in treatment and mortality in people after initial entry to methadone and buprenorphine treatment. *Addiction*. 2009;104(7):1193-1200.

135. Sordo L, Barrio G, Bravo MJ, et al. Mortality risk during and after opioid substitution treatment: Systematic review and meta-analysis of cohort studies. *bmj*. 2017;357:j1550.

136. Cornish R, Macleod J, Strang J, Vickerman P, Hickman M. Risk of death during and after opiate substitution treatment in primary care: Prospective observational study in UK General Practice Research Database. *Bmj*. Oct 26 2010;341:c5475. doi:10.1136/bmj.c5475

137. Degenhardt L, Randall D, Hall W, Law M, Butler T, Burns L. Mortality among clients of a state-wide opioid pharmacotherapy program over 20 years: Risk factors and lives saved. *Drug Alcohol Depend*. Nov 1 2009;105(1-2):9-15. doi:10.1016/j.drugalcdep.2009.05.021

138. Larochelle MR, Stopka TJ, Xuan Z, Liebschutz JM, Walley AY. Medication for opioid use disorder after nonfatal opioid overdose and mortality. *Ann Intern Med*. Mar 19 2019;170(6):430-431. doi:10.7326/l18-0685

139. Pierce M, Bird SM, Hickman M, Marsden J, Dunn G, Jones A, Millar T. Impact of treatment for opioid dependence on fatal drug-related poisoning: A national cohort study in England. *Addiction*. Feb 2016;111(2):298-308. doi:10.1111/add.13193

140. Alexopoulos GS, Reynolds III M, Charles F, Bruce ML, et al. Reducing suicidal ideation and depression in older primary care patients: 24-month outcomes of the PROSPECT study. *American Journal of Psychiatry*. 2009;166(8):882-890.

141. Bruce ML, Ten Have TR, Reynolds III CF, et al. Reducing suicidal ideation and depressive symptoms in depressed older primary care patients: A randomized controlled trial. *Jama*. 2004;291(9):1081-1091.

142. Gallo JJ, Hwang S, Joo JH, Bogner HR, Morales KH, Bruce ML, Reynolds CF. Multimorbidity, depression, and mortality in primary care: randomized clinical trial of an evidence-based depression care management program on mortality risk. *Journal of general internal medicine*. 2016;31(4):380-386.

143. Kulesza M, Watkins KE, Ober AJ, Osilla KC, Ewing B. Internalized stigma as an independent risk factor for substance use problems among primary care patients: Rationale and preliminary support. *Drug and Alcohol Dependence*. 2017;180:52-55.

144. Ober AJ, Watkins KE, Hunter SB, Lamp K, Lind M, Setodji CM. An organizational readiness intervention and randomized controlled trial to test strategies for implementing substance use disorder treatment into primary care: SUMMIT study protocol. *Implementation Science*. 2015;10:66.

145. Ober AJ, Watkins KE, Lamp K, et al. *SUMMIT study protocol: Step-by-step procedures for providing screening, brief intervention and treatment services to primary care patients with alcohol or opioid use disorders, TL-219-NIDA*. 2017. Accessed March 20, 2019. https://www.rand.org/pubs/tools/TL219.html

146. Ober AJ, Watkins KE, McCullough CM, Setodji CM, Osilla K, Hunter SB. Patient predictors of substance use disorder treatment initiation in primary care. *J Subst Abuse Treat*. Jul 2018;90:64-72. doi:10.1016/j.jsat.2018.04.004

147. Osilla KC, Watkins KE, D'Amico EJ, McCullough CM, Ober AJ. Effects of motivational interviewing fidelity on substance use treatment engagement in primary care. *Journal of Substance Abuse Treatment*. 2018;87:64-69.

148. Setodji CM, Watkins KE, Hunter SB, McCullough C, Stein BD, Osilla KC, Ober AJ. Initiation and engagement as mechanisms for change caused by collaborative care in opioid and alcohol use disorders. *Drug Alcohol Depend*. Nov 1 2018;192:67-73. doi:10.1016/j.drugalcdep.2018.07.027

149. Watkins KE, Ober AJ, McCullough CM, et al. Predictors of treatment initiation for alcohol use disorders in primary care. *Drug and Alcohol Dependence*. 2018;191:56-62.

150. Rando J, Broering D, Olson JE, Marco C, Evans SB. Intranasal naloxone administration by police first responders is associated with decreased opioid overdose deaths. *The American Journal of Emergency Medicine*. 2015;33(9):1201-1204.

151. Wakeman SE, Bowman SE, McKenzie M, Jeronimo A, Rich JD. Preventing death among the recently incarcerated: An argument for naloxone prescription before release. *Journal of Addictive Diseases*. 2009;28(2):124-129.

152. Meyers RJ, Wolfe BL. *Get your loved one sober: Alternatives to nagging, pleading and threatening*. Hazelden; 2004.

153. Osilla KC, Pedersen ER, Tolpadi A, Howard SS, Phillips JL, Gore KL. The feasibility of a web-intervention for military veteran spouses concerned about their partner's alcohol misuse. *Journal of Behavioral Health Services and Research*. 2016:1-17.

154. Smith JE, Meyers RJ. *Motivating substance abusers to enter treatment: Working with family members*. 1st ed. The Guilford Press; 2007.

155. Halford WK, Osgarby SM. Alcohol abuse clients presenting with marital problems. *Journal of Family Psychology*. 1993;6:245-254.

156. Thomas EJ, Agar RD. Unilateral family therapy with the spouses of uncooperative alcohol abusers. In: O'Farrell TJ, ed. *Treating alcohol problems: Marital and family interventions*. Guilford Press; 1993:3-33.

157. Copello A, Orford J. Addiction and the family: Is it time for services to take notice of the evidence? *Addiction*. 2002;97(11):1361-1363.

158. National Institute on Drug Abuse. Medications to treat opioid use disorder. Accessed March 25, 2020, https://d14rmgtrwzf5a.cloudfront.net/sites/default/files/21349-medications-to-treat-opioid-use-disorder.pdf

159. Bagley SM, Peterson J, Cheng DM, Jose C, Quinn E, O'Connor PG, Walley AY. Overdose education and naloxone rescue kits for family members of individuals who use opioids: Characteristics, motivations, and naloxone use. *Substance abuse*. 2015;36(2):149-154.

160. Davis C, Webb D, Burris S. Changing law from barrier to facilitator of opioid overdose prevention. *The Journal of Law, Medicine & Ethics*. 2013;41(1_suppl):33-36.

161. Strang J, Manning V, Mayet S, Titherington E, Offor L, Semmler C, Williams A. Family carers and the prevention of heroin overdose deaths: Unmet training need and overlooked intervention opportunity of resuscitation training and supply of naloxone. *Drugs: Education, Prevention and Policy*. 2008;15(2):211-218.

162. Gryczynski J, Mitchell SG, Jaffe JH, O'Grady KE, Olsen YK, Schwartz RP. Leaving buprenorphine treatment: Patients' reasons for cessation of care. *J Subst Abuse Treat*. Mar 2014;46(3):356-61. doi:10.1016/j.jsat.2013.10.004

163. Patient-Centered Outcomes Research Institute (PCORI). Comparative effectiveness of significant other-enhanced OBOT and OBOT in primary care. Accessed March 23, 2020, https://www.pcori.org/research-results/2019/comparative-effectiveness-significant-other-enhanced-obot-and-obot-primary

164. Carteret M. Cultural values of Latino patients and families. Accessed April 3, 2020, https://www.dimensionsofculture.com/2011/03/cultural-values-of-latino-patients-and-families/

165. Katzman JG, Takeda MY, Greenberg N, et al. Association of take-home naloxone and opioid overdose reversals performed by patients in an opioid treatment program. *JAMA Network Open*. 2020;3(2):e200117-e200117. doi:10.1001/jamanetworkopen.2020.0117

166. Mueller SR, Walley AY, Calcaterra SL, Glanz JM, Binswanger IA. A review of opioid overdose prevention and naloxone prescribing: Implications for translating community programming into clinical practice. *Subst Abus*. 2015;36(2):240-53. doi:10.1080/08897077.2015.1010032

167. Wheeler E, Jones TS, Gilbert MK, Davidson PJ, Centers for Disease C, Prevention. Opioid overdose prevention programs providing naloxone to laypersons - United States, 2014. *MMWR Morb Mortal Wkly Rep*. 2015;64(23):631-635.

168. Williams AV, Marsden J, Strang J. Training family members to manage heroin overdose and administer naloxone: Randomized trial of effects on knowledge and attitudes. *Addiction*. 2014;109(2):250-259.

169. Giglio RE, Li G, DiMaggio CJ. Effectiveness of bystander naloxone administration and overdose education programs: A meta-analysis. *Injury Epidemiology*. 2015;2(1):10.

170. Seal KH, Thawley R, Gee L, et al. Naloxone distribution and cardiopulmonary resuscitation training for injection drug users to prevent heroin overdose death: A pilot intervention study. *Journal of Urban Health*. 2005;82(2):303-311.

171. Walley AY, Xuan Z, Hackman HH, et al. Opioid overdose rates and implementation of overdose education and nasal naloxone distribution in Massachusetts: Interrupted time series analysis. *Bmj*. 2013;346:f174.

172. Carter GL, Clover K, Whyte IM, Dawson AH, D'Este C. Postcards from the EDge: 24-month outcomes of a randomised controlled trial for hospital-treated self-poisoning. *The British Journal of Psychiatry*. 2007;191(6):548-553.

173. Hassanian-Moghaddam H, Sarjami S, Kolahi A-A, Carter GL. Postcards in Persia: Randomised controlled trial to reduce suicidal behaviours 12 months after hospital-treated self-poisoning. *The British Journal of Psychiatry*. 2011;198(4):309-316.

174. Luxton DD, June JD, Comtois KA. Can postdischarge follow-up contacts prevent suicide and suicidal behavior? *Crisis*. 2013;

175. Motto JA, Bostrom AG. A randomized controlled trial of postcrisis suicide prevention. *Psychiatric services*. 2001;52(6):828-833.

176. Landes SJ, Kirchner JE, Areno JP, et al. Adapting and implementing Caring Contacts in a Department of Veterans Affairs emergency department: A pilot study protocol. *Pilot and Feasibility Studies*. 2019;5(1):115.

177. Luxton DD, Thomas EK, Chipps J, et al. Caring letters for suicide prevention: Implementation of a multi-site randomized clinical trial in the US military and veteran affairs healthcare systems. *Contemporary Clinical Trials*. 2014;37(2):252-260.

178. Comtois KA, Kerbrat AH, DeCou CR, Atkins DC, Majeres JJ, Baker JC, Ries RK. Effect of augmenting standard care for military personnel with brief caring text messages for suicide prevention: A randomized clinical trial. *JAMA Psychiatry*. May 1 2019;76(5):474-483. doi:10.1001/jamapsychiatry.2018.4530

179. Blevins CA, Weathers FW, Davis MT, Witte TK, Domino JL. The Posttraumatic Stress Disorder Checklist for DSM-5 (PCL-5): Development and initial psychometric evaluation. *Journal of traumatic stress*. Dec 2015;28(6):489-98. doi:10.1002/jts.22059

180. Weiner BJ. A theory of organizational readiness for change. *Implementation Science*. Oct 19 2009;4:67. doi:10.1186/1748-5908-4-67

181. Curran GM, Bauer M, Mittman B, Pyne JM, Stetler C. Effectiveness-implementation hybrid designs: Combining elements of clinical effectiveness and implementation research to enhance public health impact. *Medical care*. Mar 2012;50(3):217-26. doi:10.1097/MLR.0b013e3182408812

182. Thorpe KE, Zwarenstein M, Oxman AD, et al. A Pragmatic-Explanatory Continuum Indicator Summary (PRECIS): A tool to help trial designers. *Journal of Clinical Epidemiology*. 2009;62(5):464-475.

183. Tunis SR, Stryer DB, Clancy CM. Practical clinical trials: Increasing the value of clinical research for decision making in clinical and health policy. *Jama*. Sep 24 2003;290(12):1624-32. doi:10.1001/jama.290.12.1624

184. NIH Collaboratory. Introduction to pragmatic clinical trials. Accessed June 25, 2018, https://www.nihcollaboratory.org/Products/Introduction%20to%20pragmatic%20clinical%20trials.pdf

185. NIH Collaboratory. Rethinking clinical trials: A living textbook of pragmatic clinical trials. Accessed June 25, 2018, http://www.rethinkingclinicaltrials.org/

186. Torgerson DJ. Contamination in trials: Is cluster randomisation the answer? *BMJ*. 2001;322(7282):355-357.

187. New Mexico Department of Health. Health status of New Mexico 2018, slides 2, 3, and 249. Accessed March 25, 2019, https://nmhealth.org/data/view/general/2230/

188. University of Washington, Psychiatry & Behavioral Sciences, Division of Population Health, AIMS Center. Patient-centered integrated behavioral health care: Principles & tasks checklist. Accessed June 3, 2020, https://aims.uw.edu/sites/default/files/CollaborativeCarePrinciplesAndComponents_2014-12-23.pdf

189. Coleman K, Austin BT, Brach C, Wagner EH. Evidence on the chronic care model in the new millennium. *Health affairs*. 2009;28(1):75-85.

190. Wagner EH, Austin BT, Von Korff M. Organizing care for patients with chronic illness. *The Milbank quarterly*. 1996;74(4):511-44.

191. Unutzer J, Katon W, Callahan CM, et al. Collaborative care management of late-life depression in the primary care setting: A randomized controlled trial. *Jama*. Dec 11 2002;288(22):2836-45.

192. Leshner AI, Mancher, M (Eds.). *Medications for Opioid Use Disorder Save Lives*. 2019.

193. Volkow ND, Frieden TR, Hyde PS, Cha SS. Medication-assisted therapies--tackling the opioid-overdose epidemic. *The New England journal of medicine*. May 29 2014;370(22):2063-6. doi:10.1056/NEJMp1402780

194. Von Korff M, Gruman J, Schaefer J, Curry SJ, Wagner EH. Collaborative management of chronic illness. *Ann Intern Med*. Dec 15 1997;127(12):1097-102.

195. University of Washington, Psychiatry & Behavioral Sciences, Division of Population Health, AIMS Center. Behavioral health integration and collaborative care: Registry strategies in medical settings. Accessed June 3, 2020, https://aims.uw.edu/sites/default/files/Collaborative%20Care%20Registry%20Requirements%20Guide_2019.pdf

196. Mynors-Wallis LM, Gath DH, Day A, Baker F. Randomised controlled trial of problem solving treatment, antidepressant medication, and combined treatment for major depression in primary care. *BMJ*. 2000;320(7226):26-30.

197. Zhang A, Park S, Sullivan JE, Jing S. The effectiveness of problem-solving therapy for primary care patients' depressive and/or anxiety disorders: A systematic review and meta-analysis. *The Journal of the American Board of Family Medicine*. 2018;31(1):139-150.

198. Sloan DM, Marx BP, Bovin MJ, Feinstein BA, Gallagher MW. Written exposure as an intervention for PTSD: A randomized clinical trial with motor vehicle accident survivors. *Behav Res Ther*. 2012;50(10):627-635. doi:10.1016/j.brat.2012.07.001

199. Sloan DM, Marx BP, Resick PA, et al. Study design comparing written exposure therapy to cognitive processing therapy for PTSD among military service members: A noninferiority trial. *Contemporary Clinical Trials Communications*. 2019;17:100507.

200. Sloan DM, Marx BP, Lee DJ, Resick PA. A brief exposure-based treatment vs cognitive processing therapy for posttraumatic stress disorder: A randomized noninferiority clinical trial. *JAMA Psychiatry*. 2018;75(3):233-239.

201. Sale E, Hendricks M, Weil V, Miller C, Perkins S, McCudden S. Counseling on access to lethal means (CALM): An evaluation of a suicide prevention means restriction training program for mental health providers. *Community Mental Health Journal*. 2018/04/01 2018;54(3):293-301. doi:10.1007/s10597-017-0190-z

202. Suicide Prevention Resource Center. CALM: Counseling on Access to Lethal Means. Accessed March 27, 2020, https://www.sprc.org/resources-programs/calm-counseling-access-lethal-means

203. U.S. Department of Veterans Affairs. Lethal means safety & suicide prevention. Accessed March 27, 2020, https://www.mirecc.va.gov/lethalmeanssafety/

204. Substance Abuse and Mental Health Services Administration. *SAMHSA opioid overdose prevention toolkit: Five essential steps for first responders. HHS publication no. (-SMA) 13-4742.* 2013. https://www.integration.samhsa.gov/opioid_toolkit_firstresponders.pdf

205. National Institute on Drug Abuse. Opioid overdose reversal with naloxone (narcan, evzio). Accessed March 17, 2020, https://www.drugabuse.gov/related-topics/opioid-overdose-reversal-naloxone-narcan-evzio

206. Reger MA, Luxton DD, Tucker RP, et al. Implementation methods for the Caring Contacts suicide prevention intervention. *Professional Psychology: Research and Practice*. 2017;48(5):369-377.

207. Moyers TB, Manuel JK, Ernst D. Motivational interviewing treatment integrity coding manual 4.2.1. Unpublished manual. Accessed June 3, 2020, https://casaa.unm.edu/download/MITI4_2.pdf

208. Belsher BE, Evatt DP, Liu X, Freed MC, Engel CC, Beech EH, Jaycox LH. Collaborative care for depression and posttraumatic stress disorder: Evaluation of collaborative care fidelity on symptom trajectories and outcomes. *Journal of General Internal Medicine*. 2018;33(7):1124-1130.

209. Kroenke K, Strine TW, Spitzer RL, Williams JB, Berry JT, Mokdad AH. The PHQ-8 as a measure of current depression in the general population. *J Affect Disord*. 2009;114(1-3):163-173.

210. Brown E, Schutze M, Taylor A, et al. Use of medications for treatment of opioid use disorder among US Medicaid enrollees in 11 states, 2014-2018. *Jama*. 2021;326(2):154-164.

211. Burns M, Tang L, Chang CCH, Kim JY, Ahrens K, Allen L, Donohue J. Duration of medication treatment for opioid-use disorder and risk of overdose among Medicaid enrollees in 11 states: a retrospective cohort study. *Addiction*. 2022;117(12):3079-3088.

212. O'Connor AM, Cousins G, Durand L, Barry J, Boland F. Retention of patients in opioid substitution treatment: a systematic review. *PloS one*. 2020;15(5):e0232086.

213. American Medical Association. 8 reasons patients don't take their medications. Accessed September 29, 2023, https://www.ama-assn.org/delivering-care/patient-support-advocacy/8-reasons-patients-dont-take-their-medications#:~:text=Medication%20nonadherence%E2%80%94when%20patients%20don,for%20patients%20with%20chronic%20disease

214. Benjamin RM. Medication adherence: helping patients take their medicines as directed. *Public health reports*. 2012;127(1):2-3.

215. Pizzicato LN, Hom JK, Sun M, Johnson CC, Viner KM. Adherence to buprenorphine: an analysis of prescription drug monitoring program data. *Drug and Alcohol Dependence*. 2020;216:108317.

216. Ronquest NA, Willson TM, Montejano LB, Nadipelli VR, Wollschlaeger BA. Relationship between buprenorphine adherence and relapse, health care utilization and costs in privately and publicly insured patients with opioid use disorder. *Substance Abuse and Rehabilitation*. 2018;9:59-78.

217. Martin SA, Chiodo LM, Bosse JD, Wilson A. The next stage of buprenorphine care for opioid use disorder. *Annals of Internal Medicine*. 2018;169(9):628-635.

218. National Quality Forum. *Behavioral health 2016–2017: technical report*. 2017.

219. Button D, Cook R, King C, et al. Correlates of days of medication for opioid use disorder exposure among people living with HIV in Northern Vietnam. *International Journal of Drug Policy*. 2022;100:103503.

220. Fishman M, Vo HT, Burgower R, Ruggiero M, Rotrosen J, Lee J, Nunes E. Treatment trajectories during and following a medication trial for opioid use disorder: Moving from research as usual to treatment as usual. *Journal of addiction medicine*. 2020;14(4):331.

221. Chan KS, Fowles JB, Weiner JP. Electronic health records and the reliability and validity of quality measures: a review of the literature. *Medical Care Research and Review*. 2010;67(5):503-527.

222. Gianfrancesco MA, Goldstein ND. A narrative review on the validity of electronic health record-based research in epidemiology. *BMC medical research methodology*. 2021;21(1):1-10.

223. Parsons A, McCullough C, Wang J, Shih S. Validity of electronic health record-derived quality measurement for performance monitoring. *Journal of the American Medical Informatics Association*. 2012;19(4):604-609. doi:10.1136/amiajnl-2011-000557

224. Posner K, Oquendo MA, Gould M, Stanley B, Davies M. Columbia Classification Algorithm of Suicide Assessment (C-CASA): Classification of suicidal events in the FDA's pediatric suicidal risk analysis of antidepressants. *American Journal of Psychiatry*. 2007;164(7):1035-1043.

225. Löwe B, Unützer J, Callahan CM, Perkins AJ, Kroenke K. Monitoring depression treatment outcomes with the patient health questionnaire-9. *Medical care*. 2004;42(12):1194-1201.

226. Cigrang JA, Rauch SA, Mintz J, et al. Moving effective treatment for posttraumatic stress disorder to primary care: A randomized controlled trial with active duty military. *Families, Systems, & Health*. 2017;35(4):450-462.
