# Supplementary material for: Collaborative Care for Opioid Use Disorder and Mental Illness: The CLARO Randomized Clinical Trial
Source: JAMA Intern Med. 2025 Dec 29;186(2):168–80. doi: 10.1001/jamainternmed.2025.7036 (PMC12750335; doi:10.1001/jamainternmed.2025.7036)
Supplement: Supplement 2. — Statistical Analysis Plan [file jamainternmed-e257036-s002.pdf]

CLARO Statistical Analysis Plan
November 15, 2024

Table of Contents

1 Administrative Information *3*
1.1 SAP Details 3
1.2 SAP Revisions 3
2 Introduction *4*
2.1 Overview 4
2.1.1 Primary Objectives 4
2.1.2 Secondary Objectives 4
2.1.3 Patient population 5
2.1.4 Inclusion/exclusion criteria 5
2.2 Blinding 5
2.3 Definitions of study arms 5
2.4 Competitive Revision 6
2.5 Definitions of outcomes 6
2.5.1 Primary outcomes 6
2.5.2 Secondary outcomes 9
2.5.3 Exploratory outcomes 13
2.5.4 Other measures 15
2.6 Study design 15
2.7 Randomization 16
2.8 Interim analyses 16
3 Statistical Analyses *16*
3.1 Consort Diagram 16
3.2 Combining CC-COD+ and CC-COD 17
3.3 Baseline Characteristics 18
3.4 Statistical models 20
3.4.1 Notation 20
3.4.2 Overview and General Principles 20
3.4.3 Binary outcomes 21
3.4.4 Continuous outcomes 22
3.4.5 Survival outcome 22
3.4.6 Moderation analyses 22
3.4.7 Mediation analyses 23
3.5 Statistical power 23
3.5.1 Primary outcomes 23
3.5.2 Secondary outcomes 24
3.4.3 Exploratory outcomes 27

3.5 Primary outcome results 27

- Administrative Information

SAP Details

Statistical analysis plan (SAP) for Randomized Controlled Trial Comparing Collaborative Care and Enhanced Usual Care for Patients with Co-occurring Opioid Use Disorders and Depression and/or Post-traumatic Stress Disorder (PTSD). The study is referred to as CLARO (Collaboration Leading to Addiction Treatment and Recovery from Other Stresses) and is also referred to as the parent study.

On 8/31/2022 a Competitive Revision (CR—CLARO+) was awarded to enroll additional patients into an additional study arm. CLARO+ adds additional clinical interventions used by the care coordinator to the Collaborative Care Model being tested by CLARO.

Because the CR did not enroll sufficient individuals to allow for separate analyses, in consultation with our Research Advisory Board, the NIMH scientific officer and the NIMH project officer, it was decided to combine patients enrolled into the parent study intervention arm, with patients enrolled into the CLARO+ study intervention arm for all analyses. The justification for this was that the objective of the trial was to test the Collaborative Care Model for individuals with co-occurring disorders, and the model was identical across both the CLARO and CLARO+ trials.

This SAP describes analyses for patients enrolled into both CLARO and CLARO+.

National Clinical Trial Identified Number: NCT04559893 (CLARO).
National Clinical Trial Identified Number: NCT04634279 (CLARO+)

Protocol Version Number: 13.1

SAP Revisions

SAP revision history is summarized below, with a summary of changes from previous versions listed first.

SAP Date	Summary of Revisions Made	Rationale
11/15/24	• Revised secondary and exploratory outcomes including:<br>• cut MOUD initiation and engagement measures<br>• modified continuity of care and MOUD access to be buprenorphine-specific<br>• modified definitions of access to treatment and quality of care secondary outcomes<br>• modified drug use frequency secondary outcome to be opioid use frequency<br>• created alcohol use, drug use frequency, opioid use severity, and opioid overdose risk behaviors exploratory outcomes<br>• added stimulant use frequency as an exploratory outcome<br>• Cut all-cause mortality analysis	• Tailor analyses based on time/budget constraints<br>• Ensure all design and analysis descriptions reflect latest knowledge and assumptions (e.g., actual sample size, best practices, data limitations, resource constraints, etc.) prior to unblinding

	• Revised baseline characteristic specifications (Table 5)<br>• Added plans to combine parent study and competitive revision (CR) samples, and revised text describing CR accordingly<br>• Added details on model covariates (prior MOUD and site variable) specifications to account for potential small sample sizes<br>• Revised power calculations with final enrollment numbers, parent study and CR combined samples, and modified outcomes<br>• Updated moderators and mediators<br>• Modified methods for handling missing data in the baseline and follow-up surveys.<br>• Added detail on non-response weight calculations, including criteria for determining if non-response weights are necessary	
06/25/23	• Edited specifications of two primary outcomes (MOUD access and continuity of care)	• Original, planned, MOUD outcomes were deemed not possible to calculate due to limitations of the EMR data
02/16/21	• Addition of detailed description of the statistical models to be used, including approach for handling missing follow-up data<br>• Modification of primary and secondary analyses and removal of analyses that leveraged repeated measures<br>• Updated power calculations to utilize a simulation-based calculation<br>• Addition of a description of the composite statistical hypothesis	• Address queries from 09/14/20 DSMB meeting

• Introduction

○ Overview

▪ Primary Objectives

To evaluate the effectiveness of collaborative care for opioid use disorder (OUD) and co-occurring depression or PTSD (CC-COD) on patient initiation of medications for OUD (MOUD), quality of care for OUD, depression symptoms, and PTSD symptoms relative to enhanced usual care (EUC).

▪ Secondary Objectives

To test mediators (patient experiences with care and working alliance with the Care Coordinator) of treatment quality and patient-reported outcomes and, in exploratory analyses, test moderators of access, quality, and outcomes compared with patients assigned to EUC.

- Patient population

The study population is patients ≥18 years of age attending one of 18 primary care clinics in five large healthcare organizations (First Choice Community Healthcare, Providence St. John's Primary Care, Hidalgo Medical Services, University of New Mexico and Los Angeles County Department of Healthcare Services) in New Mexico and California who have probable OUD and either depression or PTSD. Both patients enrolled in the parent study (CLARO) and the CR (CLARO+) are included.

- Inclusion/exclusion criteria

Patients will be eligible to participate in the study if they meet the following inclusion criteria:

- Consider this clinic to be their usual source of care
- Age 18 or older
- Probable OUD diagnosis
- Probable PTSD or major depression diagnosis
- Speak and understand English or Spanish
- Have capacity to give informed consent
- Provide a signed and dated informed consent form.

Pregnant women will not be excluded.

Patient participants will not be eligible to participate in the study if they meet the following exclusion criterion:

- The patient requires immediate medical (emergency procedure needed) or psychiatric intervention (i.e., self-injured, active psychosis).

- Blinding

The research staff will be blind to the treatment assignment and participant outcomes until database lock, except for research assistants at the point of randomization. All aspects of this analysis plan were finalized prior to unblinding. Clinic staff and participants will not be blind to the treatment assignment, as it is not possible to deliver the intervention with blinding.

- Definitions of study arms

CC-COD intervention. The intervention is based on a service delivery approach that uses multi-faceted interventions to improve access and quality of care. It is based on Wagner's Chronic Care Model and subsequent modifications. Care coordinators use Motivational Interviewing and meet with patients individually for at least 13 visits over six months. In the first two months, the Care Coordinator meets weekly with the patient. In month three, meetings are biweekly. In months four through six, the Care Coordinator and the patient meet once a month. Meetings can be in-person or by phone, and ideally occur in-person prior to an appointment with the patients' primary care provider (PCP) so that the Care Coordinator can relay information (e.g., symptoms, insights regarding barriers to care) to the PCP before the appointment with the patient. Meetings can also be conducted more frequently or for longer than six months should the care team decide it is best for the patient. The first meeting between the Care Coordinator and the patient occurs in a care initiation visit, where the Care Coordinator develops patient trust and engagement, assesses various patient domains, discusses the conditions to target, links the patient to care, and coordinates linkages with the PCP and BHP. Assessment includes asking the patient about substance use severity using the PROMIS-7, depression symptoms using the PHQ-9, PTSD symptoms using the PCL-5, along with the WellRX to assess social needs, PEG Pain Monitor, and social support measures. The Care Coordinator then

collaborates with the patient using MI to assess treatment experiences and barriers to care, provides information about treatment options, and then coordinates next steps with the patient. After the care initiation visit, the Care Coordinators meets with the patient in monitoring visits for the remainder of the six-month intervention period. Care monitoring visits focus on engagement, assessment, and linkages. Five opioid use questions are asked at each visit, whereas the PROMIS-7, PCL-5, and PHQ-9 are administered monthly. In this care model, the Care Coordinator is supported by a behavioral health consultant (BHC) and a Care Coordinator supervisor with expertise in supervising community health workers. The Care Coordinator, BHC, and Care Coordinator supervisor meet weekly to discuss the Care Coordinator's caseload. Throughout the study period, the Care Coordinator enters patient information into a clinical registry, a patient caseload tool with four main purposes: (1) track population-level outcomes and engagement, (2) prompt the Care Coordinator with reminders and alerts to ensure accountable outreach when a patient has an upcoming appointment or needs a higher-level of care, (3) prompt treatment-to-target by showing trends in patient symptom severity scores and flags the Care Coordinator when to consult with the BHC, and (4) facilitate caseload review between the Care Coordinator, BHC, and Care Coordinator supervisor through caseload-level reports that display patient-level ID numbers of those who should be discussed.

Enhanced Usual Care (EUC) intervention. EUC includes both the evidenced-based psychotherapy (i.e., Problem Solving Therapy for depression and Written Exposure Therapy for PTSD) and pharmacotherapy (i.e., MOUD and psychotropic medications for MDD/PTSD) provided in CC-COD, but the primary difference is the absence of a care management team and a clinical registry to coordinate care with the patients in EUC.

- Competitive Revision

On 6/6/2022, approximately 5 months after we started data collection, we submitted a competitive revision (CR) which was awarded 8/31/2022. The CR uses the same patient population and inclusion/exclusion criteria as the original study and introduced a new study arm, where patients received up to 3 additional clinical components: support person education, Naloxone training, and Caring Contacts. We refer to this modified intervention as CC-COD+ and the modified study as CLARO+.

- Definitions of outcomes

  - Primary outcomes

Table 1 defines the primary outcomes of this study. The hypotheses associated with these outcomes are as follows:

1. We hypothesize that patients with a new OUD episode of care who are randomized to CC-COD/CC-COD+ will receive buprenorphine within fewer days after that care episode than new OUD patients who are randomized to enhanced usual care. Alternatively, the null hypothesis is that there will be no difference in the number of days until receipt of buprenorphine with a new OUD episode of care.
2. We hypothesize that patients who are randomized to CC-COD/CC-COD+ will have more days of buprenorphine treatment within 180 days of study enrollment than patients who are randomized to enhanced usual care. Alternatively, the null hypothesis is that there will be no difference in the number of days of cumulative buprenorphine treatment within 180 days of study enrollment.
3. We hypothesize that, among patients with probable depression at study enrollment, those who are randomized to CC-COD/CC-COD+ will have a greater reduction in depression symptoms 6 months after study enrollment compared to those who are randomized to enhanced usual care. Alternatively, the null hypothesis is that there will be no difference in depression symptoms 6 months after study enrollment among those with probable depression at enrollment.
4. We hypothesize that, among patients with probable PTSD at study enrollment, those who are randomized to CC-COD/CC-COD+ will have a greater reduction in PTSD symptoms 6 months after study enrollment compared to those who are randomized to enhanced usual care. Alternatively, the null hypothesis is that there will be no difference in PTSD symptoms 6 months after study enrollment among those with probable PTSD at enrollment.

A union-intersection test will be used to test the overall success of the study that combines the four primary outcomes into a single composite hypothesis. In particular, the null hypothesis is the intersection of the four primary outcome null hypotheses (i.e., all null hypotheses are true), and the alternative is the union of the alternative hypotheses (i.e., at least one alternative hypothesis is true). As described in subsequent sections, either a Bonferroni or Benjamini-Hochberg correction on the individual hypotheses will be used to control the error rate of this composite test.

Table 1: Definition of primary outcomes

Outcome	Population[a]	Definition	Measure Scale	Source
Buprenorphine access	Study participants with a new episode of OUD care (no care for at least 30 days prior)	Number of days until first buprenorphine prescription after study enrollment	Time-to-event	Survey, PMP
Buprenorphine continuity of care	Study participants not on methadone at baseline	The cumulative number of days the patient receives buprenorphine during the 180 days after study enrollment.	Continuous	Survey, PMP
MDD symptom severity	Study participants with probable MDD at baseline (PHQ-8 ≥ 10)	PHQ-9 at 6 months	Sum of items (0-27)	Survey
PTSD symptom severity	Study participants with probable PTSD at baseline (PC-PTSD-5 ≥ 3)	PCL-5 at 6 months	Sum of items (0-80)	Survey

[a] Study participants means all study participants who consented and were randomized to the intervention or to EUC.

- Secondary outcomes

There are 13 pre-specified secondary outcomes, which are described in

Table *2*. After drafting of the initial SAP, but prior to analyzing outcomes and breaking the blind, some secondary outcomes were modified, cut, or reclassified as exploratory, as detailed in the revision table in section 1.2.

Table 2: Definition of secondary outcomes

Outcome	Population[b]	Definition	Measure Scale	Source
**Mental Health**				
Access to MDD and/or PTSD treatment	Study participants who did not have any visits (behavioral health treatment or medication) for MDD and/or PTSD in 30 days prior to study enrollment	Receipt of medication and/or behavioral treatment associated with an MDD or PTSD diagnosis within 30 days of study enrollment (initial) or within 180 days of study enrollment (any)	Binary	EMR, Survey
Quality of care for MDD	Study participants with probable MDD at baseline (PHQ-8 ≥ 10) and a new episode of MDD care (no MDD care for at least 30 days prior to enrollment)	Four psychotherapy visits in the first six months or an adequate (60 day) medication trial for new episodes of MDD care (completed within 6 months)	Binary	EMR, Survey
Quality of care for PTSD	Study participants with probable PTSD at baseline (PC-PTSD-5 ≥ 3). and a new episode of PTSD care (no PTSD care for at least 30 days prior to enrollment)	Four psychotherapy visits in the first six months or an adequate (60 days) medication trial for new episodes of PTSD care (completed within 6 months)	Binary	EMR, Survey
MDD remission	Study participants with probable MDD at baseline (PHQ-8 ≥ 10)	PHQ-9 < 5 at 6 months	Binary, based on sum of items (0-27)	Survey
MDD response	Study participants with probable MDD at baseline (PHQ-8 ≥ 10)	PHQ-9 score at 6 months less than 50% of baseline score	Binary, based on sum of items (0-27)	Survey
PTSD remission	Study participants with probable PTSD at baseline (PC-PTSD-5 ≥ 3)	PCL-5 < 34 at 6 months	Binary, based on sum of items (0-80)	Survey
PTSD response	Study participants with probable PTSD at baseline (PC-PTSD-5 ≥ 3)	PCL-5 score at 6 months less than 50% of baseline score	Binary, based on sum of items (0-80)	Survey
Active suicidal ideation	All study participants	Dichotomized Columbia Suicide Severity Rating Scale at 6 months: answer YES to Question 3, 4, and/or 5 and/or YES to Question 7	Binary	Survey
**Substance Use**				
Opioid use frequency	All study participants	Days of opioid use in the past 30 days from NSDUH	Total days of opioids use (0-30)	Survey
Opioid overdose events	All study participants	Opioid overdose events in the previous three months	Binary, at least 1 event	Survey

**Overall Health**				
Physical health functioning	All study participants	Veterans RAND 12-item Health Survey (VR-12) – physical health subscale	MEPS[1] standard	Survey
Mental health functioning	All study participants	Veterans RAND 12-item Health Survey (VR-12) – mental health subscale	MEPS standard	Survey

[b] Study participants means all study participants who consented and were randomized to the intervention or to EUC.

- Exploratory outcomes

There are five potential exploratory outcomes, which are described in Table 3.

Table 3: Definition of Exploratory Outcomes

Outcome	Population[c]	Definition	Measure Scale	Source
Drug use frequency	All study participants	Maximum days of use in the past 30 days for five drug categories using items from NSDUH (prescription opioids, heroin, cocaine/crack, methamphetamine/ other stimulants, and tranquilizers/sedatives)	Total days of use of most frequent drug (0-30)	Survey
Stimulant use frequency	All study participants	Days of stimulant use (cocaine/crack, methamphetamine/ other stimulants) in the past 30 days from NSDUH	Total days of stimulant use (0-30)	Survey
Alcohol use	All study participants	Alcohol Use Disorder Identification Test – Consumption (AUDIT-C) for the previous 3 months	Sum of items (0-12)	Survey
Opioid overdose risk behaviors	All study participants	Opioid Overdose Risk Assessment	Sum of items (0-32)	Survey
Opioid use severity	All study participants	Patient-Reported Outcomes Measurement Information System (PROMIS) Substance Use Short Form for the previous 30 days	Sum of items (7-35)	Survey

- Other measures

Table 4 provides the definition of additional measures that will be used as potential mediators and moderators in exploratory analyses described below.

Table 4: Definition of additional potential mediation and moderation variables

Measure	Instrument	Measure Scale	Source
**Potential Moderators**			
Trauma/Interpersonal Violence	PTSD Checklist for DSM-5, description of worst event	Categorical*	Survey
Sex	Sex on current birth certificate	Categorical**	Survey
Ethnicity	Patient-reported ethnicity	Binary (Hispanic/ non-Hispanic)	Survey
Pain	PEG Pain Monitor, for past week	Mean of 3 items (0-10)	Survey
Disability and impairment	Sheehan Disability Scale	Sum of 3 items (0-30)	Survey
Stimulant use frequency	Days of stimulant use (cocaine/crack, methamphetamine/ other stimulants) in the past 30 days from NSDUH	Total days of use (0-30)	Survey
Housing status	Homelessness Screening Clinical Reminder	Categorical***	Survey
Number of care coordinator visits	Care coordinator entry of visits with patient	Continuous	Registry
**Potential Mediators**			
Clinician communication	CAHPS ECHO	0-100	Survey
Ability to quickly access treatment	CAHPS ECHO	0-100	Survey
Overall rating of treatment	CAHPS ECHO	0-100	Survey
Patient and care coordinator working alliance	Working Alliance Inventory	Mean of 12 items (min=1, max=5)	Survey

*no trauma, trauma w/o IPV, trauma w/ IPV; **male, female, gender neutral; ***stably housed, unstably housed, unhoused

- Study design

This study is a stratified randomized trial testing CC-COD versus EUC, with strata defined by prior MOUD exposure and primary care clinic.

As part of the CR, we initially planned to compare CC-COD+ to CC-COD, with MOUD continuity of care as the primary outcome. However, despite sustained and targeted efforts, recruitment was lower than expected, and we did not reach our targeted enrollment goals for either CC-COD or CC-COD+. Based on input from our research advisory board and NIH project officer, we now plan to combine the CC-COD and CC-COD+ samples when analyzing the data for additional information about the potential effectiveness of the intervention. Since the Collaborative Care Model is identical across both trials, and the only difference between the arms is in the additional clinical modules supported by CC-COD+, combining the active CC-COD and CC-COD+ arms for an exploratory analysis should provide additional precision around the estimated treatment effect for CC-COD/CC-COD+.

The study's original target sample size was 900, with an additional 300 patients targeted for the CR, for a combined target of 1200. Our final sample size was 797 (729 following the original protocol and 68 following the protocol from the CR). This analysis plan has been modified to include actual final power calculations calculated prior to breaking the blind but after all data collection ended.

- Randomization

After a baseline assessment, study participants will be randomized 1:1 into CC-COD/CC-COD+ or EUC. A stratified randomization design will be used, with the strata determined by primary care clinic and prior MOUD exposure. A randomization list will be generated for each stratum and include randomly permuted block sizes of 2 and 4. We will stratify on prior MOUD to mitigate confounding, as patients who have used MOUD previously are more likely to initiate MOUD subsequently. Research staff will access the randomization module in REDCap. Staff will enter which prior MOUD exposure stratum and clinic the patient is in, and the intervention arm assignment will be generated.

We extended the randomized trial from the original study to implement CC-COD+. All participating clinics started the study with enrolled patients being randomized between CC-COD and EUC. Three health systems then transitioned to randomizing patients between CC-COD+ and EUC. The remaining health system did not transition to CC-COD+ because they did not enroll sufficient patients to justify transitioning to CC-COD+. Table 5 provides a timeline of when health systems transitioned to CC-COD+.

Table 5: Competitive Revision Recruitment Timeline

**Site Groups**	Date of CC-COD+ Transition
Health System 1 (includes 7 clinics)	08/29/2022
Health System 3 (includes 3 clinics)	07/13/2023
Health System 4 (includes 3 clinics)	08/28/2023

- Interim analyses
  - None

  - Statistical Analyses

- Consort Diagram

The consort diagram for the study is shown below, in Figure 1. This combines original study and CR populations and was generated prior to unblinding.

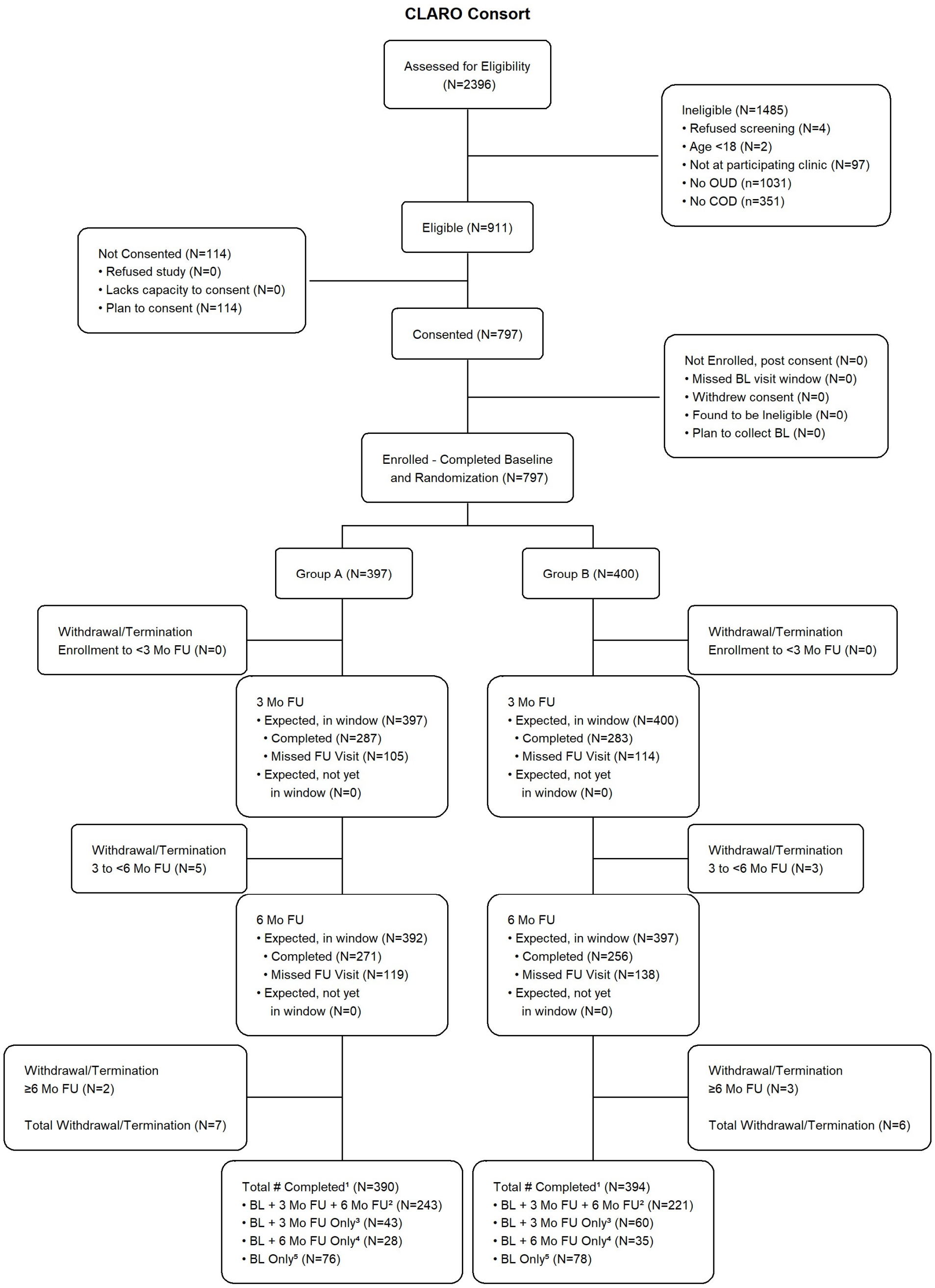

Figure 1. Consort Diagram

○ Combining CC-COD+ and CC-COD

All analyses described below were initially planned for the original study but will be conducted for the combined original study and CR population. We will also conduct sensitivity analyses with just the CC-COD population.

o Baseline Characteristics

We will conduct descriptive analyses comparing CC-COD/CC-COD+ and EUC characteristics at baseline using a Chi-squared test for categorical variables and a two sample t-test otherwise. Table 6 provides the demographic characteristics of the study participants at baseline. Table 7 provides all outcomes at baseline and at the 6-month follow-up.

Table 6: Sociodemographics and other covariates at baseline

		**Treatment Assignment**	
	**Overall**	**CC-COD**	**EUC**
**Age, n (%)**			
18-30 years			
31-40 years			
41-50 years			
51 years and older			
**Sex, n (%)**			
Male			
Female			
Gender-neutral sex designation (X)			
**Race/Ethnicity, n (%)**			
White, Non-Hispanic			
Hispanic			
Other race, Non-Hispanic			
**Education, n (%)**			
Less than high school diploma			
High school graduate/GED			
Any college/higher-level education			
**Marital Status, n (%)**			
Never Married			
Married/Partnered			
Divorced/Separated/ Widowed			
**Prior MOUD, n (%)**			
**Health system, n (%)**			

Health system 1			
…			
Health system 4			

Table 7: Outcomes at baseline and 6-months

	Baseline			6-Months		
	**Overall**	**CC-COD**	**EUC**	**Overall**	**CC-COD**	**EUC**
**Primary outcomes**						
Buprenorphine access, mean (SD)[1]						
Buprenorphine continuity of care, mean (SD)						
PTSD, n (%)						
PTSD Symptoms, mean (SD)						
MDD, n (%)						
MDD Symptoms, mean (SD)						
**Secondary outcomes**						
Access to MDD and/or PTSD treatment, n (%)						
Quality of care for MDD, n (%)						
Quality of care for PTSD, n (%)						
MDD remission, n (%)						
MDD response, n (%)						
PTSD remission, n (%)						
PTSD response, n (%)						
Suicidality, n (%)						
Opioid Use Frequency, mean (SD)						
Opioid Overdose Events, n (%)						
Physical Health Functioning, mean (SD)						
Mental Health Functioning, mean (SD)						
**Exploratory outcomes**						
Drug use frequency, mean (SD)						
Stimulant use frequency, mean (SD)						
Alcohol use, mean (SD)						
Opioid Overdose Risk Behaviors, mean (SD)						
Opioid use severity, mean (SD)						

NOTES: [1]Gray cells indicate outcomes not measured at baseline

- Statistical models

  - Notation

Table 8 provides notation used to describe the statistical models. We note that the sample size per primary care clinic where not large enough to support the use of fixed effects by clinics in our regression models. Instead, we utilize fixed effects for health systems. When the sample sizes for the smallest health systems prove too small (e.g., less than 30 for continuous outcomes), we will collapse the smallest health systems together for the purposes of our regression models.

Table 8: Notation used to describe the statistical models.

Notation	Description
$\boldsymbol{X}_{ij}$	The baseline characteristics of participant *i* at health system *j*, including both demographics and other baseline characteristics.
P_{ij}	An indicator of prior MOUD treatment for participant *i* at health system *j*.
T_{ij}	The random treatment assignment of participant *i* at health system *j*.
y_{ij}	An outcome measure for participant *i* at health system *j*.
R_{ij}	An indicator denoting whether participant *i* at health system *j* responded to the 6-month follow-up survey.
M_{ij}	A mediator of the outcome-treatment relationship for participant *i* at health system *j*.

  - Overview and General Principles

The primary analyses will be performed for the intention-to-treat population, which consists of all randomized subjects. All individual statistical hypotheses will be tested using two-sided tests, with adjustment for multiple testing using a Bonferroni or Benjamini-Hochberg correction. Primary outcomes will be analyzed at a Type I error rate of 1.25% to control the family-wise error rate at 5% (e.g., so 0.05/4=0.0125 when using a Bonferroni correction or the first p-value threshold for significance with Benjamini-Hochberg). Similarly, secondary and exploratory outcomes will be analyzed at Type I error rates to control family-wise error rate at 5% within domain. See Table 2 and 3 for definition of secondary outcome domains and our exploratory outcomes.

Outcomes derived from the follow-up surveys are subject to nonresponse, and statistical hypotheses based on these outcomes will be tested using nonresponse weighted models, if necessary. Traditionally, a logistic regression model can be specified predicting response using the demographic characteristics listed in Table , the baseline characteristics listed in Table , and the randomized treatment assignment. The model is given by:

$$\log\left(\frac{\Pr(R_{ij}=1)}{1-\Pr(R_{ij}=1)}\right) = \eta_j + \boldsymbol{X}_{ij}\boldsymbol{\theta} + \gamma T_{ij} + \rho P_{ij}$$

where η_j represent health system fixed effects. The nonresponse weight for participant i in health system j is given by $w_{ij} = \frac{1}{p_{ij}}$, where $p_{ij} = \Pr(R_{ij} = 1)$. However, we will utilize a nonparametric machine learner to estimate our nonresponse weights to allow for more complex relationships between the predictors variables and the odds of being a responder, thereby producing more high-quality nonresponse weights if they are deemed necessary.

To assess whether there is a need for use of nonresponse weights in our final analyses, we will assess how representative our sample of responders is for each outcome using effect sizes (ES) differences. If most of the ES differences are small (e.g., less than 0.1 or 0.2), we will feel good about the representativeness of our sample of responders to the original baseline sample and not utilize nonresponse weights in our final analyses.

In addition, there is potential for item-level missingness among those who completed the baseline and follow-up surveys. We will perform a descriptive analysis that summarizes this type of missing data. Based on prior studies, we expect such missingness to be isolated to very few subjects, and plan to use a single imputation. For the baseline survey, we will use the following criteria for cleaning up missing data:

- For missing items that are part of a composite measure,
    a. if the scoring algorithm of the composite measure allows for missingness, we will score the composite using that algorithm
    b. if the scoring algorithm includes a sum of items, and fewer than 50% of component variables are missing, we will score the composite as a sum of the observed items scaled to account for the missing item(s)
    c. for all others, we will impute the score using a model that includes the treatment assignment, the health system, and prior MOUD exposure as predictors
- For all other missing items, we will use mean imputation within treatment assignment and health system

For the follow-up survey data cleaning, we plan to perform logical imputation solely for missing items that are part of a composite measure, and we will use a single imputation for composite measures using criteria a. and b. described above.

If the item-level missingness is determined to be more prevalent than anticipated, we will implement a multiple imputation strategy. Five multiply imputed datasets will be generated, and standard rules for combining multiply imputed datasets with nonresponse weighting will be applied.[2] Imputations will be generated with sequential predictive mean matching using the R package *mice*.[3]

- Binary outcomes

Binary outcomes will be analyzed using a logistic regression model including health system, prior MOUD exposure, and treatment assignment as predictors. The logistic regression model is given by:

$$\log\left(\frac{\Pr(y_{ij} = 1)}{1 - \Pr(y_{ij} = 1)}\right) = \alpha_j + \nu P_{ij} + \beta T_{ij}$$

where α_j are health system-level fixed effects and e^{β} is the odds ratio for CC-COD/CC-COD+ versus EUC. The statistical hypotheses from the logistic regressions will be of the form:

$H_0: \beta = 0$ vs
$H_1: \beta \neq 0.$

The estimated effects from these models will be converted to risk differences using the recycled prediction approach to estimate marginal effects.[4] Both odds ratios and risk differences may be reported to improve interpretation.

If necessary, outcomes that require elements from the 6-month follow-up survey may be weighted to account for nonresponse as previously described. Additionally, if we do not have sufficient sample size to support modeling P_{ij} (prior MOUD treatment), then it will be removed from a given model. We will assess this by examining a cross-frequency of P_{ij} with out outcome y_{ij} and ensure that we have at least 20 individuals per cell.

- Continuous outcomes

Continuous outcomes will be analyzed using a linear regression model including health system, prior MOUD exposure, and treatment assignment as predictors. The linear regression model is given by:

$$\mathrm{y_{ij}} = \alpha_j + \nu P_{ij} + \beta T_{ij} + e_{ij}$$

where α_j are clinic-level fixed effects, β is the effect of CC-COD over EUC, and e_{ij} is an independent and identically distributed mean zero error term. The statistical hypotheses from the linear regressions will be of the form:

$H_0: \beta = 0$ vs
$H_1: \beta \neq 0.$

If necessary, outcomes that require elements from the 6-month follow-up survey may be weighted to account for nonresponse as previously described. Again, if we do not have sufficient sample size to support modeling P_{ij} (prior MOUD treatment), then it will be removed from a given model. We will assess this for each continuous outcome by examining if we have at least 30 individuals with and without prior MOUD treatment in our analytic sample.

- Survival outcome

Buprenorphine access, the survival outcome that is measured in the PMP and survey will be analyzed using a Cox proportional hazard regression model including health-system, prior MOUD exposure, and treatment assignment as predictors. The Cox proportional regression model is given by:

$$\mathrm{h(t)} = h_0(t) * \exp\left(\alpha_j + \nu P_{ij} + \beta T_{ij}\right)$$

where α_j are health system-level fixed effects, and e^{β} is the hazard ratio effect of CC-COD over EUC. The statistical hypotheses from the Cox proportional hazard regression will be of the form:

$H_0: \beta = 0$ vs
$H_1: \beta \neq 0.$

Outcomes that are measured as part of the 6-month follow-up survey may be weighted to account for nonresponse as previously described. Again, if we do not have sufficient sample size to support modeling P_{ij} (prior MOUD treatment), then it will be removed from a given model. We will assess this for each continuous outcome by examining if we have at least 30 individuals with and without prior MOUD treatment in our analytic sample. We will right censor all observations for those individuals who did not ever receive buprenorphine during the course of follow-up for these analyses.

- Moderation analyses

All analyses of subgroups are considered exploratory. We will explore the data to understand if any of the following baseline factors moderate the effect of the intervention:

- Sex
- Ethnicity
- Stimulant use (methamphetamine or cocaine use)
- Housing status
- History of trauma/interpersonal violence
- Pain (PEG)
- Disability and impairment (SDS)
- Number of care coordinator visits

Definitions for these moderation variables are described in Table 4. Additional moderators may be explored as warranted. Statistical hypotheses testing whether the effect of the intervention varies by these factors will be tested by the inclusion of an interaction between the moderating factor and the treatment assignment into the previously described models. We will also utilize propensity score weighting if deemed necessary here to correct for potential imbalances between the treatment groups within levels of the moderators.[5]

- Mediation analyses

We will assess several potential mediators in this study, including whether patient experiences of care and working alliance with the care coordinator at 3 months mediate the impact of CC-COD on patient. Specifically, we consider the following mediators:

1. clinician communication,
2. ability to quickly access treatment,
3. overall rating of treatment, and
4. patient Care Coordinator working alliance using a modified Working Alliance Inventory.

To ensure a proper temporal ordering of the treatment, mediators, and outcomes, all outcomes for the mediation analyses will be measured at 6-months, while the mediators will be measured at the 3-month follow-up.

All mediation analyses will follow the approach described in Imai, et al. (2010),[6] including the technical assumptions necessary to identify causal mediation effects. This methodology requires the specification of a model predicting the mediator using only baseline information, and a model predicting the outcome using both baseline information and the mediator. For a continuous mediator and outcome, the models have the form:

$$\mathrm{M_{ij}} = \eta_j + \varphi T_{ij} + \boldsymbol{X}_{ij}\boldsymbol{\omega} + \epsilon_{ij}$$
$$\mathrm{y_{ij}} = \alpha_j + \beta T_{ij} + \delta M_{ij} + \boldsymbol{X}_{ij}\boldsymbol{\tau} + e_{ij},$$

where $\varphi * \delta$ represents the causal mediation effect under necessary technical assumptions. For binary outcomes or mediators, the models will be specified as logistic regression models. We will use the *mediation* package for the R computing environment to conduct these analyses.[7]

- Statistical power

All calculations are for 80% power at a Type I error rate of 1.25%, which accounts for the multiple primary outcomes using a Bonferroni correction to control the family-wise error rate at 5% (0.05/4 = 0.0125). Actual calculations use the observed loss to follow up rate in the final sample.

- Primary outcomes

We used the observed distributions for our continuous variables including the following: for the planned power calculations were:

1. We observed a mean number of days until accessing Buprenorphine of 26.0 with a standard deviation of 31.9. Our total sample size for this analysis was 137 individuals with 6-month follow-up data.
2. Among those who initiate medication for OUD, the mean number of days of continuous treatment for OUD is 133.0 with a standard deviation is 54.1. The total sample size for this analysis was 392 individuals with 6-month follow-up data.
3. Among those with probable depression at enrollment (PHQ-8 ≥ 10), the mean depression symptoms score (PHQ-8) at 6 months of 11.5 with a standard deviation of 6.6. The total sample size for this analysis was 405 individuals with 6-month follow-up data.
4. Among those with probable PTSD at enrollment (PC-PTSD-5 ≥ 3), the mean PTSD symptoms score (PCL-5) at 6 months of 32.2 with a standard deviation of 17.8. The total sample size for this analysis was 368 individuals with 6-month follow-up data.

Under these observed outcome distributions as well as the final number of individuals with observed outcome values in our data, we have 80% power to detect:

1. A hazard ratio of at least 0.45 if we find a protective effect and see fewer days until accessing Buprenorphine. This corresponds to at least a hazard ratio of 2.25 if the direction were reversed or a 27-percentage point difference if the outcome was modeled as a binary measure. Notably, a previous study of Collaborative Care for opioid and alcohol use disorders found a 22-percentage point increase over enhanced usual care so if the effect is large in CLARO, we may be able to detect it.[8]
2. 18.3 additional days of continuous OUD treatment within the first 180 days. A growing body of evidence suggests the mortality is lower during OUD treatment, and that mortality is increased in the first four weeks after treatment cessation.[9]
3. A 2.2 point reduction in depression symptoms (PHQ-8). This provides power to detect effects below the clinically important difference for individual change of 5 points.[10]
4. A 6.23 point reduction in PTSD symptoms (PCL-5). A previous study of delivering PTSD treatment in primary care setting to active duty military found a reduction in PTSD symptoms of 7 points.[11]

- Secondary outcomes

Secondary endpoints are grouped into domains of conceptually related endpoints, and the outcomes will be analyzed adjusting for multiple comparison within domain using a Bonferroni correction. Table 8 provides the grouping of the secondary outcomes into domains, along with the observed outcome distributions, the minimum detectible effect size at 80% power and a family-wise error rate of 5%, and a reference effect size (if available). The vast majority of secondary outcomes are sufficiently powered to detect clinically meaningful effects or effects found in similar interventions.

Table 9: Minimum detectible effect size for secondary outcomes

Outcome	Observed distributions	Minimum detectible effect size	Reference effect size
**Mental Health**			
Access to MDD and/or PTSD treatment	• N=259<br>• 65.5% access treatment	19.2 percentage points	None
Quality of care for MDD	• N=210<br>• 8.8% with quality care	18.9 percentage point	16 percentage points for medication adherence[12] at 3 months
Quality of care for PTSD	• N=300<br>• 9% with quality care	15.3 percentage point	Expect similar to MDD
MDD remission	• N=405<br>• 16.0% remission	15.0 percentage points	15 percentage points[13]
MDD response	• N=405<br>• 26.2% response	15.8 percentage points	20 percentage points from CALM[13]<br>or<br>23 percentage points from STEPS-UP[11]
PTSD remission	• N=368<br>• 56.2% remission	17.8 percentage points	Similar to MDD remission
PTSD response	• N=365<br>• 21.6% response	17.7 percentage points	18 percentage points[11]
Active suicidal ideation	• N=523<br>• 24.2% at 6-months	14.5 percentage point	Any reduction
**Substance Use**			
Opioid use frequency	• N=523<br>• Mean (sd) = 4.3 (9.6)	0.27 standard deviations; 2.6 raw scale	None

Opioid overdose events	• N=525<br>• 2.1% at 6-months	5.9 pct point reduction	None
**Overall Health**			
Physical health functioning	• N=525<br>• Mean (sd) = 36.8 (12.0)	3.2 points	Similar to mental health functioning
Mental health functioning	• N=525<br>• Mean (sd) = 37.3 (13.0)	3.5 points	2.3 points[11]

3.4.3 Exploratory outcomes

The exploratory endpoints will be analyzed adjusting for multiple comparison using a Bonferroni or Benjamini-Hochberg correction. Table 10 provides the outcomes, along with the key observed outcome distributions in the sample for the power calculation, the minimum detectible effect size at 80% power and a family-wise error rate of 5%, and a reference effect size (if available). All exploratory outcomes are sufficiently powered to detect clinically meaningful effects or effects found in similar interventions.

Table 10: Minimum detectible effect size for exploratory outcomes

Outcome	Assumptions	Minimum detectible effect size	Reference effect size
Drug use frequency	• N=525<br>• Mean (sd) = 6.1 (10.7)	0.3 standard deviations;<br>3.2 raw scale	None
Stimulant use frequency	• N=525<br>• Mean (sd) = 3.9 (8.7)	0.3 standard deviations;<br>2.6 raw scale	None
Alcohol use	• N=522<br>• Mean (sd) = 1.6 (2.8)	0.31 standard deviations;<br>0.87 raw scale	2 points between mild, moderate, severe.[14]
Opioid overdose risk behaviors	• N=525<br>• Mean (sd) = 12.1 (5.5)	0.3 standard deviations;<br>1.7 raw scale	25% reduction.[15]
Opioid use severity	• N=525<br>• Mean (sd) = 50.1 (7.9)	0.3 standard deviations;<br>2.4 raw scale	None

3.5 Primary outcome results

Table 11: Estimated effect of CC-COD over EUC for primary outcomes, including descriptive statistics by treatment arm. Descriptive statistics include counts and percentages for binary outcomes and means and standard deviations for all others.

Outcome	CC-COD	EUC	Effect Estimate[1]
Buprenorphine Access			
Buprenorphine Continuity of Care			
MDD Symptoms			
PTSD Symptoms			

NOTE: [1] The effect estimate for all outcomes is reported as the difference between CC-COD and EUC from models that include fixed effects for clinic, prior MOUD exposure, and treatment assignment. Binary outcomes are modeled using logistic regression and converted to a marginal risk difference using recycled predictions.

References

1. Selim AJ, Rogers W, Fleishman JA, Qian SX, Fincke BG, Rothendler JA, Kazis LE. Updated U.S. population standard for the Veterans RAND 12-item Health Survey (VR-12). Qual Life Res. 2009;18(1):43-52. Epub 2008/12/04. doi: doi: 10.1007/s11136-008-9418-2. PubMed PMID: 19051059.
2. Seaman SR, White IR, Copas AJ, Li L. Combining multiple imputation and inverse-probability weighting. Biometrics. 2012;68(1):129-37. doi: doi: 10.1111/j.1541-0420.2011.01666.x. PubMed PMID: 22050039.
3. Buuren Sv, Groothuis-Oudshoorn K. mice: Multivariate imputation by chained equations in R. Journal of Statistical Software. 2010:1-68. doi: doi: 10.1111/j.0006-341X.1999.00652.x.
4. Graubard BI, Korn EL. Predictive margins with survey data. Biometrics. 1999;55(2):652-9.
5. Griffin BA, Schuler MS, Cefalu M, Ayer L, Godley M, Greifer N, Coffman DL, McCaffrey DF. A Tutorial for Propensity Score Weighting for Moderation Analysis With Categorical Variables: An Application Examining Smoking Disparities Among Sexual Minority Adults. Med Care. 2023;61(12):836-45. Epub 20231002. doi: 10.1097/mlr.0000000000001922. PubMed PMID: 37782463; PMCID: PMC10840831.
6. Imai K, Keele L, Tingley D. A general approach to causal mediation analysis. Psychological methods. 2010;15(4):309-34. Epub 2010/10/20. doi: doi: 10.1037/a0020761. PubMed PMID: 20954780.
7. Tingley D, Yamamoto T, Hirose K, Keele L, Imai K. Mediation: R package for causal mediation analysis. Journal of Statistical Software. 2014;59(5):1-38.
8. Watkins KE, Ober AJ, Lamp K, Lind M, Setodji C, Osilla KC, Hunter SB, McCullough CM, Becker K, Iyiewuare PO, Diamant A, Heinzerling K, Pincus HA. Collaborative Care for Opioid and Alcohol Use Disorders in Primary Care: The SUMMIT Randomized Clinical Trial. JAMA Intern Med. 2017;177(10):1480-8. doi: 10.1001/jamainternmed.2017.3947. PubMed PMID: 28846769; PMCID: PMC5710213.
9. Sordo L, Barrio G, Bravo MJ, Indave BI, Degenhardt L, Wiessing L, Ferri M, Pastor-Barriuso R. Mortality risk during and after opioid substitution treatment: Systematic review and meta-analysis of cohort studies. bmj. 2017;357:j1550. doi: doi: 10.1136/bmj.j1550.
10. Löwe B, Unützer J, Callahan CM, Perkins AJ, Kroenke K. Monitoring depression treatment outcomes with the patient health questionnaire-9. Medical Care. 2004;42(12):1194-201.
11. Engel CC, Jaycox LH, Freed MC, Bray RM, Brambilla D, Zatzick D, Litz B, Tanielian T, Novak LA, Lane ME. Centrally assisted collaborative telecare for posttraumatic stress disorder and depression among military personnel attending primary care: A randomized clinical trial. JAMA Internal Medicine. 2016;176(7):948-56. doi: doi: 10.1001/jamainternmed.2016.2402.
12. Katon W, Von Korff M, Lin E, Simon G, Walker E, Unützer J, Bush T, Russo J, Ludman E. Stepped collaborative care for primary care patients with persistent symptoms of depression: A randomized trial. Archives of General Psychiatry. 1999;56(12):1109-15. doi: doi: 10.1001/archpsyc.56.12.1109.
13. Roy-Byrne P, Craske MG, Sullivan G, Rose RD, Edlund MJ, Lang AJ, Bystritsky A, Welch SS, Chavira DA, Golinelli D. Delivery of evidence-based treatment for multiple anxiety disorders in primary care: A randomized controlled trial. Jama. 2010;303(19):1921-8. doi: doi: 10.1001/jama.2010.608
14. Williams EC, Rubinsky AD, Chavez LJ, Lapham GT, Rittmueller SE, Achtmeyer CE, Bradley KA. An early evaluation of implementation of brief intervention for unhealthy alcohol use in the US Veterans Health Administration. Addiction. 2014;109(9):1472-81. doi: doi: 10.1111/add.12600.
15. Bohnert AS, Bonar EE, Cunningham R, Greenwald MK, Thomas L, Chermack S, Blow FC, Walton M. A pilot randomized clinical trial of an intervention to reduce overdose risk behaviors among emergency department patients at risk for prescription opioid overdose. Drug and alcohol dependence. 2016;163:40-7. doi: doi: 10.1016/j.drugalcdep.2016.03.018.
