# Supplementary material for: Collaborative Care for Opioid Use Disorder and Mental Illness: The CLARO Randomized Clinical Trial
Source: JAMA Intern Med. 2025 Dec 29;186(2):168–80. doi: 10.1001/jamainternmed.2025.7036 (PMC12750335; doi:10.1001/jamainternmed.2025.7036)
Supplement: Supplement 3. — eTable 1. Baseline Characteristics by Response Status and Weighting for MDD Symptom Severity Outcome eTable 2. Baseline Characteristics by Response Status and Weighting for PTSD Symptom Severity Outcome eTable 3. As-Treated Adjusted Effect Estimates for Primary, Secondary, and Exploratory Outcomes [file jamainternmed-e257036-s003.pdf]

SUPPLEMENTAL ONLINE CONTENT

Watkins KE, Osilla KC, McCullough CM, et al. Collaborative care for opioid use disorder and mental illness: the CLARO randomized clinical trial. *JAMA Intern Med.* Published online December 29, 2025. doi:10.1001/jamainternmed.2025.7036

eTable 1. Baseline Characteristics by Response Status and Weighting for MDD Symptom Severity Outcome

eTable 2. Baseline Characteristics by Response Status and Weighting for PTSD Symptom Severity Outcome

eTable 3. As-Treated Adjusted Effect Estimates for Primary, Secondary, and Exploratory Outcomes

This supplemental material has been provided by the authors to give readers additional information about their work.

© 2025 Watkins KE et al. *JAMA Intern Med*.

eTable 1. Baseline Characteristics by Response Status and Weighting for MDD Symptom Severity Outcome

Response Rate = 65%	Baseline Target Sample (N= 621)	Responders, Unweighted	Responders, Weighted	Nonresponders, Unweighted
Assigned to Collaborative Care, %	0.51	0.49	0.48	0.54**
**Sociodemographic characteristics**				
Age in years, %				
18-30	0.21	0.18	0.19	0.27**
31-40	0.36	0.36	0.36	0.36
41-50	0.21	0.22	0.22	0.20
51+	0.22	0.25	0.24	0.17**
Sex, %				
Male	0.46	0.42	0.44	0.55**
Female	0.54	0.58	0.56	0.45**
Race and Ethnicity, %				
White, non-Hispanic	0.24	0.26	0.26	0.20**
Hispanic/Latino	0.68	0.66	0.66	0.71**
Other/more than one race, non-Hispanic	0.09	0.08	0.08	0.09
Education, %				
Less than high school	0.31	0.28	0.30	0.36*
High school or equivalent	0.30	0.28	0.29	0.33*
Some college or more	0.40	0.44	0.41	0.31*
Marriage status, %				
Never married	0.35	0.34	0.35	0.37
Married/living with partner	0.37	0.37	0.38	0.37
Widowed/divorced/separated	0.28	0.28	0.28	0.27
Living in stable housing (3 months), %	0.86	0.88	0.87	0.80**
Any current legal trouble, %	0.19	0.17	0.18	0.22**
**Clinical characteristics**				
Days with opioid use (30 days), mean	7.58	6.98	7.44	8.72**
PROMIS t-score (30 days)[a], mean	53.67	52.74*	53.29	55.42**
Opioid overdose events (3 months), %				
No opioid overdose	0.95	0.96	0.96	0.91**
Any opioid overdose	0.06	0.04	0.04	0.09**
Days with stimulant use (30 days), mean	6.04	4.75*	5.35	8.46**
Days with any drug use (30 days), mean	10.03	8.83*	9.49	12.28**
AUDIT sum (3 months)[b], mean	4.31	4.26	4.31	4.39
MOUD history (30 days), %				
No MOUD	0.20	0.19	0.20	0.23**
On methadone as prescribed	0.18	0.19	0.19	0.15**

© 2025 Watkins KE et al. *JAMA Intern Med*.

On buprenorphine as prescribed	0.55	0.56	0.54	0.53
**Response Rate = 65%**	**Baseline Target Sample (N= 621)**	**Responders, Unweighted**	**Responders, Weighted**	**Nonresponders, Unweighted**
On MOUD, never/sometimes prescribed	0.07	0.06	0.07	0.09**
PHQ-9 score[c], mean	15.75	15.46	15.66	16.27**
Any history of trauma[d], %				
No trauma	0.10	0.09	0.09	0.11
Trauma, no interpersonal violence	0.53	0.54	0.54	0.51
Trauma, interpersonal violence	0.38	0.37	0.37	0.38
PCL-5 score[e], mean	40.44	39.42	40.03	37.87
PCL-5 score, NA, %	0.09	0.09	0.09	0.11
Suicidal ideation (30 days), %	0.33	0.32	0.33	0.35
VR-12 mental health component score[f], mean	36.13	35.86	35.99	36.65
VR-12 physical health component score[f], mean	31.19	31.34	31.28	30.93
PEG score (avg)[g], mean	5.63	5.69	5.68	5.53
Health System, %				
Health system 1	0.48	0.47	0.48	0.51
Health system 2	0.04	0.04	0.04	0.05
Health system 3	0.40	0.41	0.40	0.37**
Health system 4	0.08	0.08	0.08	0.08

Abbreviations: PROMIS, Patient-Reported Outcomes Measurement Information System; MOUD, medications for opioid use disorder; AUDIT, Alcohol Use Disorders Identification Test; PHQ-9, Patient Health Questionnaire-9; PCL-5, Post-Traumatic Stress Disorder Checklist for Diagnostic and Statistical Manual of Mental Disorders 5; VR-12, Veterans RAND 12 Item Health Survey; PEG, Pain, Enjoyment, General Activity tool.

[a]PROMIS t-score measures severity of opioid use disorder, with range of 0 (best) to 100 (worst), centered at 50 with a standard deviation of 10.

[b]AUDIT sum measures severity of alcohol use, with range of 0 (best) to 40 (worst).

[c]PHQ-9 measures severity of depression symptoms, with range of 0 (best) to 27 (worst).

[d]History of trauma is coded using the worst traumatic event reported as part of the PCL-5 assessment. Events are categorized as trauma with interpersonal violence if they include harm intentionally inflicted on the participant by another person.

[e]PCL-5 measures severity of PTSD symptoms, with range of 0 (best) to 80 (worst).

[f]Mental health and physical health component scores measured with VR-12, with range of 0 (worst) to 100 (best), centered at 50 with a standard deviation of 10.

[g]PEG (avg) measures severity of pain, with a range of 0 (best) to 10 (worst).

© 2025 Watkins KE et al. *JAMA Intern Med*.

eTable 2. Baseline Characteristics by Response Status and Weighting for PTSD Symptom Severity Outcome

Response Rate = 57%	Baseline Target Sample (N= 647)	Responders, Unweighted	Responders, Weighted	Nonresponders, Unweighted
Assigned to Collaborative Care, %	0.49	0.48	0.48	0.51
**Sociodemographic characteristics**				
Age in years, %				
18-30	0.23	0.20	0.21	0.27**
31-40	0.38	0.40	0.39	0.37
41-50	0.21	0.20	0.21	0.22
51+	0.18	0.20	0.19	0.14**
Sex, %				
Male	0.45	0.38*	0.41	0.55**
Female	0.55	0.62*	0.59	0.46**
Race and Ethnicity, %				
White, non-Hispanic	0.23	0.26	0.25	0.19**
Hispanic/Latino	0.69	0.66	0.67	0.73**
Other/more than one race, non-Hispanic	0.08	0.08	0.09	0.08
Education, %				
Less than high school	0.30	0.26	0.29	0.35**
High school or equivalent	0.29	0.27	0.28	0.32**
Some college or more	0.41	0.47*	0.44	0.34**
Marriage status, %				
Never married	0.36	0.33	0.35	0.40**
Married/living with partner	0.36	0.39	0.38	0.32**
Widowed/divorced/separated	0.28	0.28	0.27	0.28
Living in stable housing (3 months), %	0.86	0.89	0.88	0.82**
Any current legal trouble, %	0.21	0.16*	0.19	0.27**
**Clinical characteristics**				
Days with opioid use (30 days), mean	7.05	6.29	6.96	8.06**
PROMIS t-score (30 days)[a], mean	53.39	52.43*	53.02	54.64**
Opioid overdose events, %				
No opioid overdose (3 months)	0.95	0.96	0.95	0.94
Any opioid overdose (3 months)	0.05	0.04	0.05	0.07
Days with stimulant use (30 days), mean	5.68	4.44*	4.93	7.32**
Days with any drug use (30 days), mean	9.39	8.06*	8.83	11.14**
AUDIT sum (3 months)[b], mean	3.87	3.79	3.78	3.96
MOUD history (30 days), %				
No MOUD	0.17	0.16	0.17	0.18
On methadone as prescribed	0.19	0.19	0.20	0.18

© 2025 Watkins KE et al. *JAMA Intern Med*.

eTable 2

On buprenorphine as prescribed	0.56	0.58	0.55	0.55
**Response Rate = 57%**	**Baseline Target Sample (N= 647)**	**Responders, Unweighted**	**Responders, Weighted**	**Nonresponders, Unweighted**
On MOUD, never/sometimes prescribed	0.08	0.07	0.07	0.09**
PHQ-9 score[c], mean	13.81	13.77	13.92	13.88
Any history of trauma, %				
No trauma	0.02	0.01	0.02	0.03**
Trauma, no interpersonal violence	0.56	0.54	0.54	0.60**
Trauma, interpersonal violence	0.41	0.45	0.44	0.37**
PCL-5 score[e], mean	39.82	39.65	40.02	38.89
No traumatic event, %	0.02	0.01	0.01	0.03**
Suicidal ideation (30 days), %[1]	0.32	0.32	0.32	0.31
VR-12 mental health component score[f], mean	37.85	37.65	37.75	38.11
VR-12 mental health component score[f], mean	32.87	32.46	32.54	33.41
PEG score (avg)[g], mean	5.24	5.26	5.29	5.23
Health System, %				
Health system 1	0.48	0.47	0.48	0.50
Health system 2	0.04	0.04	0.04	0.04
Health system 3	0.41	0.43	0.41	0.38
Health system 4	0.07	0.07	0.07	0.08

Abbreviations: PROMIS, Patient-Reported Outcomes Measurement Information System; MOUD, medications for opioid use disorder; AUDIT, Alcohol Use Disorders Identification Test; PHQ-9, Patient Health Questionnaire-9; PCL-5, Post-Traumatic Stress Disorder Checklist for Diagnostic and Statistical Manual of Mental Disorders 5; VR-12, Veterans RAND 12 Item Health Survey; PEG, Pain, Enjoyment, General Activity tool.

[a]PROMIS t-score measures severity of opioid use disorder, with range of 0 (best) to 100 (worst), centered at 50 with a standard deviation of 10.

[b]AUDIT sum measures severity of alcohol use, with range of 0 (best) to 40 (worst).

[c]PHQ-9 measures severity of depression symptoms, with range of 0 (best) to 27 (worst).

[d]History of trauma is coded using the worst traumatic event reported as part of the PCL-5 assessment. Events are categorized as trauma with interpersonal violence if they include harm intentionally inflicted on the participant by another person.

[e]PCL-5 measures severity of PTSD symptoms, with range of 0 (best) to 80 (worst).

[f]Mental health and physical health component scores measured with VR-12, with range of 0 (worst) to 100 (best), centered at 50 with a standard deviation of 10.

[g]PEG (avg) measures severity of pain, with a range of 0 (best) to 10 (worst).

© 2025 Watkins KE et al. *JAMA Intern Med*.

eTable 3. As-Treated Adjusted Effect Estimates for Primary, Secondary, and Exploratory Outcomes

	Any Collaborative Care		Received key elements of collaborative care	
	Adjusted effect estimate (95% CI)	*P* value	Adjusted effect estimate (95% CI)	*P* value
Primary Outcomes				
Buprenorphine access	-10.77 (-28.77 to 7.22)	0.24	-15.62 (-41.73 to 10.50)	0.24
MOUD continuity of care	5.89 (-6.87 to 18.65)	0.37	8.24 (-9.57 to 26.05)	0.37
MDD symptom severity	-1.25 (-2.71 to 0.21)	0.09	-1.58 (-3.42 to 0.26)	0.09
PTSD symptom severity	-0.52 (-4.72 to 3.68)	0.81	-0.69 (-6.23 to 4.86)	0.81
Secondary Outcomes				
MDD remission	0.07 (-0.01 to 0.15)	0.09	0.09 (-0.01 to 0.19)	0.09
MDD response	0.04 (-0.06 to 0.14)	0.39	0.05 (-0.07 to 0.18)	0.39
PTSD remission	-0.02 (-0.13 to 0.10)	0.79	-0.02 (-0.18 to 0.13)	0.79
PTSD response	0.03 (-0.06 to 0.13)	0.51	0.04 (-0.09 to 0.17)	0.51
Suicidality	-0.04 (-0.12 to 0.04)	0.32	-0.05 (-0.15 to 0.05)	0.32
Opioid use frequency	-0.19 (-2.07 to 1.68)	0.84	-0.25 (-2.66 to 2.16)	0.84
Opioid overdose events	0.00 (-0.03 to 0.03)	0.88	0.00 (-0.03 to 0.04)	0.88
Physical health functioning	-1.67 (-3.83 to 0.50)	0.13	-2.14 (-4.93 to 0.64)	0.13
Mental health functioning	1.85 (-0.67 to 4.37)	0.15	2.38 (-0.85 to 5.60)	0.15
Exploratory Outcomes				
Stimulant use frequency	-1.01 (-2.70 to 0.68)	0.24	-1.31 (-3.48 to 0.87)	0.24
Opioid overdose risk behaviors	-1.09 (-2.14 to -0.05)	0.04	-1.41 (-2.74 to -0.08)	0.04
Opioid use disorder severity	-1.76 (-3.26 to -0.25)	0.02	-2.26 (-4.18 to -0.34)	0.02
Alcohol use severity	-0.37 (-0.90 to 0.17)	0.18	-0.47 (-1.16 to 0.22)	0.18

Abbreviations: MDD, Major Depressive Disorder; MOUD, medications for opioid use disorder; PTSD, Post-traumatic Stress Disorder,

© 2025 Watkins KE et al. *JAMA Intern Med*.
