# Supplementary material for: Collaborative Care for Opioid Use Disorder and Mental Illness: The CLARO Randomized Clinical Trial
Source: JAMA Intern Med. 2025 Dec 29;186(2):168–80. doi: 10.1001/jamainternmed.2025.7036 (PMC12750335; doi:10.1001/jamainternmed.2025.7036)
Supplement: Supplement 4. — Data Sharing Statement [file jamainternmed-e257036-s004.pdf]

Data Sharing Statement

Watkins. Collaborative Care for Opioid Use Disorder and Mental Illness. *JAMA Intern Med.* Published December 29, 2025. doi:10.1001/jamainternmed.2025.7036

Data
Additional Information: ClinicalTrials.gov IDs: NCT04559893 and NCT04634279
Data available: Yes
Data types: Deidentified participant data
How to access data: NIMH Heal repository
When available: beginning date: 04-01-2026

Supporting Documents
Document types: None

Additional Information
Who can access the data: researchers whose proposed use of the data has been approved
Types of analyses: For any purpose
Mechanisms of data availability: without investigator support from the NIMH Heal repository and a signed data access agreement
